# Comprehensive biophysical and structural profiling of alpha-actinin-2 variants reveals mechanistic diversity in hypertrophic cardiomyopathy

**DOI:** 10.1038/s41467-026-75392-z

**Published:** 2026-07-21

**Authors:** Maya Noureddine, Halina Mikolajek, Nathan Cowieson, Nikos Pinotsis, Paul Robinson, Alexandre Slater, Charles Redwood, Siobhan Loughna, Chris Denning, Fiyaz Mohammed, Katja Gehmlich

**Affiliations:** 1 Department of Cardiovascular Sciences, University of Birmingham, Birmingham, UK; 2 Diamond Light Source Ltd, Didcot, UK; 3 Institute of Structural and Molecular Biology, Birkbeck College, London, UK; 4 Division of Cardiovascular Medicine, Radcliffe Department of Medicine and British Heart Foundation Centre of Research Excellence Oxford, University of Oxford, Oxford, UK; 5 School of Life Sciences, University of Nottingham, Nottingham, UK; 6 Biodiscovery Institute, University of Nottingham, Nottingham, UK; 7 Department of Immunology and Immunotherapy, University of Birmingham, Birmingham, UK

**Keywords:** SAXS, Structural variation, Structural biology, Biophysics, Biochemistry

## Abstract

Hypertrophic cardiomyopathy (HCM) is a genetic disease associated with sudden cardiac death. Variants in alpha-actinin-2 (ACTN2), a Z-disc protein that anchors actin thin filaments have been implicated in HCM, yet their structural consequences remain poorly defined. Here, we characterise seventeen HCM-associated ACTN2 variants spanning multiple domains using an integrated and tiered workflow combining high-throughput assays, structural modelling and biophysical approaches. All variants display reduced solubility, with actin-binding domain (ABD) substitutions showing pronounced thermal instability by differential scanning fluorimetry. Modelling of nine variants predicts diverse pathogenic mechanisms including compromised actin-binding, impaired ABD regulatory conformations, disrupted dimerisation interfaces, and perturbed domain architecture. Crystal structures of two rod-domain variants reveal intact dimerisation despite modelling predictions. Actin-binding assays for ABD variants confirm altered actin engagement suggesting that binding dynamics may drive pathogenicity. Limited proteolysis indicates reduced structural stability across variants, while size-exclusion chromatography coupled with multi-angle light scattering or small-angle X-ray scattering (SEC-MALS/SAXS) shows a strong propensity for aggregation. Batch-mode SAXS further demonstrates early aggregation onset in selected ABD variants at elevated temperatures. Collectively, these findings establish that HCM-linked ACTN2 variants compromise protein integrity through multiple mechanisms, highlight the ABD as a hotspot of vulnerability and provide a potential framework for interpreting cardiomyopathy-associated variants.

## Abstract (web summary)

Inherited cardiac conditions are linked to genetic misspellings (or variants) in essential heart proteins such as alpha-actinin-2. Here, the authors uncover mechanistic diversity by which distinct genetic variants may drive disease, using a comprehensive range of structural analyses.

## Introduction

Inherited cardiomyopathies are a group of genetic cardiac conditions which significantly impair the structure and function of the heart muscle. The most prevalent of these conditions is hypertrophic cardiomyopathy (HCM), which affects approximately 1 in 500 individuals^1^. HCM is characterised by left ventricular hypertrophy, diastolic dysfunction, and arrhythmias. The latter makes sudden cardiac death a frequent feature of HCM^2^. At the molecular level, HCM involves structural and contractile defects within the cardiac myofilaments, primarily affecting the sarcomere, the fundamental contractile unit of the heart muscle.

A fundamental component of the sarcomere structure is the Z-disc, an intricate multi-protein assembly that defines the lateral boundaries of this essential muscle unit and acts as a signalling hub. Z-disc proteins play crucial roles in regulating contraction^3^. Among these, alpha-actinin-2 (ACTN2) is a primary constituent that anchors and cross-links actin thin filaments, thereby preserving the structural integrity of cardiac muscle tissue. ACTN2 forms an anti-parallel dimer, with each monomer exhibiting a molecular weight of ~103 kDa. ACTN2 consists of several well-defined domains, each contributing both to its structural integrity and function^4^. The N-terminal actin-binding domain (ABD) features two calponin homology domains (CH1, CH2) and is regulated by phospholipids, which modulate its conformational dynamics and binding to actin filaments^5^. A flexible neck region connects the ABD to a central rod domain consisting of four spectrin-like repeats (SRs 1–4) essential for dimer formation through a zipper-like configuration^6^. At the C-terminus, ACTN2 possesses a calmodulin homology domain (CaM) with two EF-hand motifs (EF12, EF34).

Numerous studies have established an association between missense variants in ACTN2 and cardiomyopathy, particularly HCM^7–9^. Most of these investigations have focused on elucidating the functional implications of these genetic variants. For example, one study identified a rare ACTN2 variant, T247M, in a patient with HCM, and further analysis using stem cell-derived cardiomyocytes demonstrated impaired relaxation and increased calcium sensitivity^7^. In another comprehensive study involving 239 patients with HCM, three ACTN2 variants (G111V, T495M, R759T) were identified, with histological analysis revealing cardiomyocyte hypertrophy^8^.

While considerable attention has been devoted to understanding the functional implications of ACTN2 variants, there remains a relative paucity of research focusing on their structural impact on protein integrity. For instance, one study used biophysical assays to assess two ACTN2 variants (A119T and G111V) using the isolated ABD region of ACTN2, revealing reduced actin-binding affinity and decreased thermal stability^10^. Similarly, another study evaluated three additional variants (A119T, M228T, T247M) using ACTN2-ABD constructs, reporting altered thermal stability and disrupted actin-binding^11^.

Although these few studies have explored the functional and structural impact of ACTN2 variants on protein architecture, an in-depth structural assessment employing specialised biophysical techniques remains markedly absent. Moreover, prior research has largely overlooked the analysis of full-length ACTN2 protein in biophysical evaluations, despite its greater physiological relevance compared to truncated constructs.

In this work, we employ a comprehensive framework aimed at assessing the structural and biophysical implications of ACTN2 variants using the full-length protein. This approach seeks to provide mechanistic insights underlying variant pathogenicity in HCM, thereby enhancing our understanding of the molecular underpinnings of this condition. In addition, this research contributes to evaluating the effectiveness of biophysical characterisation techniques in the context of assessing disease-associated genetic variants.

## Results

### Characterisation and mapping of ACTN2 variants

Missense variants in alpha-actinin-2 (ACTN2) linked to hypertrophic cardiomyopathy (HCM) were retrieved from the Human Gene Mutation Database (HGMD)^12^, and their pathogenicity was evaluated using in silico prediction tools^4^. Employing this approach, 17 variants were identified and selected for further analysis.

Interpretations derived from the ClinVar database^13^ showed that five variants were of uncertain clinical significance, and three had no associated classification (Table S1). Reports for other five variants were of conflicting interpretations between benign and pathogenic interpretations, reflecting the complexities in accurately assessing their clinical relevance. Clinical data were also retrieved by reviewing relevant reports from HGMD. Notably, most variants lack detailed clinical symptoms in their case reports (Table S1), further highlighting the necessity for comprehensive evaluation of these variants.

**Fig. 1:**
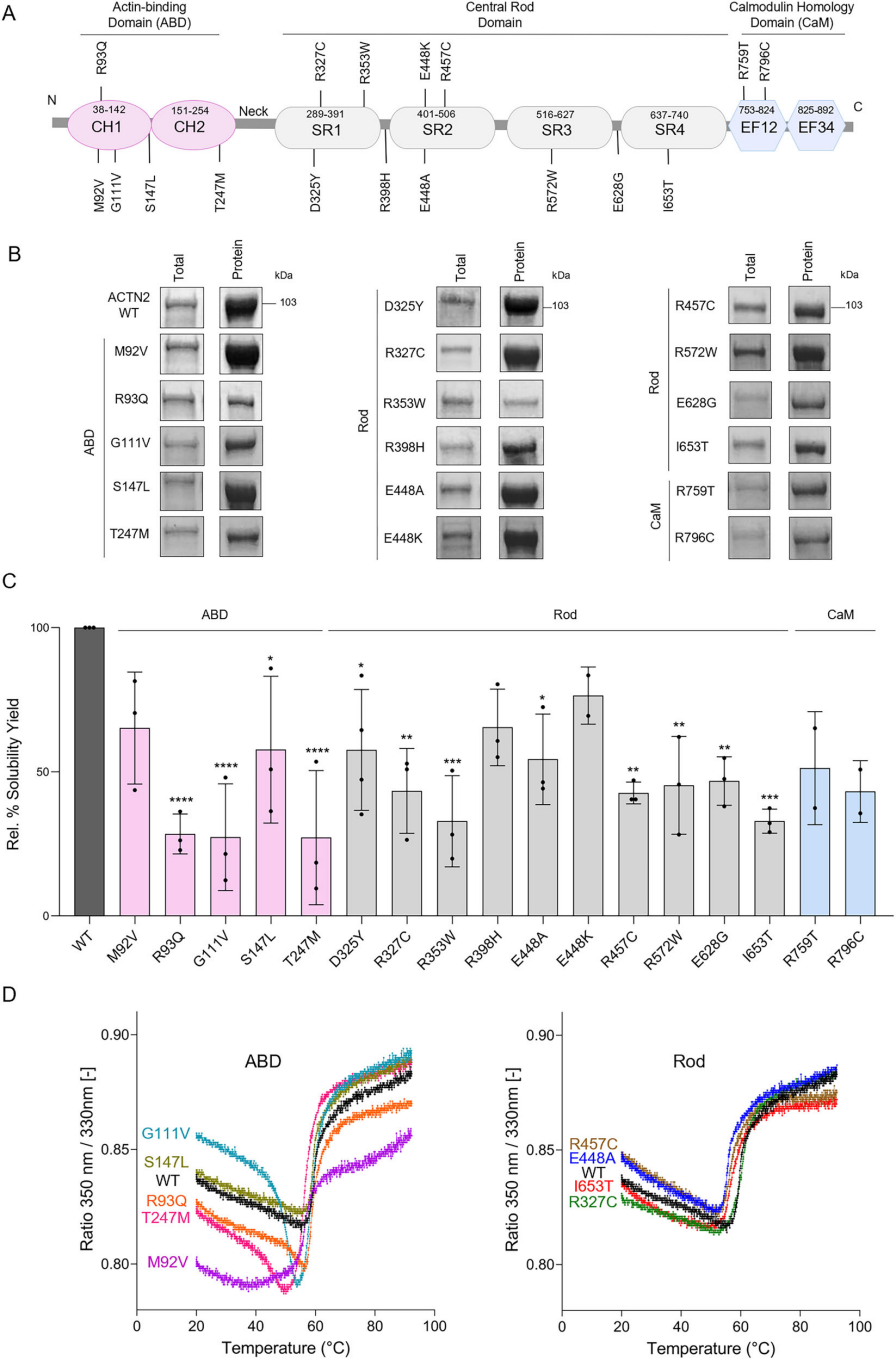
Initial assessment of the stability of the identified 17 ACTN2 HCM-linked missense variants. **A** Schematic diagram of ACTN2 monomer mapping the identified 17 ACTN2 HCM-linked missense variants distributed across the different structural domains including the actin binding domain (ABD) consisting of two calponin homology domains (CH1, CH2), neck region, central rod domain consisting of four spectrin repeats (SRs 1 to 4), and a calmodulin homology domain (CaM) consisting of two EF-hands (EF12, EF34). **B** Comparison of the solubility of WT and ACTN2 variants in *E. coli* with representative images showing total lysate (‘total’) and purified protein (‘protein’) fractions. All samples per each of the independent replicates are derived from the same experiment with gels processed in parallel. **C** Histograms showing changes in % of solubility yield of the 17 ACTN2 variants calculated as the ratio between purified protein and total lysate. Relative protein levels are normalised to WT with a value set at 100%. Data are normally distributed when tested using the Shapiro-Wilk test. Data are analysed using Ordinary One-way ANOVA with Dunnett test used for multiple comparison and values presented as mean ± standard deviation (SD), with error bars indicating SD. Values are from three independent experimental runs, except for E448K, R759T, and R796C variants with two experimental runs (excluded from statistical analysis).*p < 0.05, **p < 0.01, ***p < 0.001. *p* values of variants vs. WT are: M92V = 0.1040, R93Q < 0.0001, G111V < 0.0001, S147L = 0.0270, T247M < 0.0001, D325Y = 0.0149, R327C = 0.0014, R353W = 0.0001, R398H = 0.1079, E448A = 0.0140, E448K = undetermined [*n* = 2], R457C = 0.0012, R572W = 0.0021, E628G = 0.0029, I653T = 0.0001, R759T and R796C = undetermined [*n* = 2]. **D** Thermal denaturation profiles of the nine selected ACTN2 variants in the ABD and rod domain using differential scanning fluorimetry. Fluorescence intensity ratios (350 nm/330 nm) are measured as a function of increasing temperature (20–95 °C). Data represent mean values from two independent experimental runs. Profiles for the remaining eight variants are presented in supplementary materials (Fig. S1). Table 1 presents a summary of melting temperatures values of the 17 variants.

The 17 HCM-linked ACTN2 variants were then mapped onto the structural framework of the ACTN2 protein (Fig. 1A), enabling the identification of specific domains where these variants reside. Notably, the distribution included five variants in the actin-binding domain (ABD), ten in the central-rod module and two in the EF12-hand. Strikingly, no disease-linked variants were identified within the neck region or the EF34-hand, potentially suggesting domain-specific vulnerabilities with respect to variant-induced pathogenesis.

### ACTN2 variants show reduced solubility

We next sought to investigate whether these amino acid substitutions impact ACTN2 folding and expression levels. To evaluate this, we performed solubility assays for each variant following recombinant expression of the full-length ACTN2 protein in *E. coli*. Solubility yield was quantified as the percentage of purified protein relative to the total protein present in the lysate. Across all 17 ACTN2 variants, solubility was consistently reduced compared to the wild-type (WT) protein (Fig. 1B, C). Notably, variants located within the ABD, such as R93Q, G111V, and T247M, demonstrated marked reduction in solubility (Fig. 1B, C). Similarly, rod-domain variants R353W and I653T displayed pronounced effects (Fig. 1B, C). These findings indicate that ACTN2 variants broadly compromise protein solubility, suggesting destabilisation of folding across multiple structural domains. This widespread reduction highlights the potential for altered biophysical properties, reinforcing the need for further biochemical studies to delineate their contribution to HCM pathogenesis.

### ACTN2 variants display decreased thermal stability

While solubility analysis provided initial insights into the potential impact of ACTN2 variants on protein stability, it did not capture domain-specific alterations. To assess more subtle changes in protein thermal stability, we extended our analysis using differential scanning fluorimetry (DSF), which revealed pronounced effects in ABD variants. G111V and T247M exhibited extreme reduction in melting temperature (ΔTm₁ > 6 °C), whereas M92V and R93Q showed a moderate decrease (ΔTm₁ ≃ 4 °C) (Fig. 1D, Table 1). In contrast, S147L showed a thermal stability comparable to WT. Strikingly, some ABD variants also unfolded earlier, with an onset of unfolding temperatures (Tm₀) of 45 °C for G111V and T247M and 51 °C for R93Q (Fig. 1D, Table 1). The unfolding of these variants at lower temperatures reflects diminished structural integrity and functional stability as impaired thermostability is associated with reduced rigidity of the protein scaffold^14^. In the rod domain, R327C manifested a moderate Tm_1_ shift (ΔTm_1_ ≃ 4 °C), whereas E448A, R457C, and I653T showed only slight changes (ΔTm_1_ between 1 and 2 °C) (Fig. 1D, Table 1). Additional variants in the rod and CaM domains generally maintained WT-like thermal stability (Figs. 1D and S1, Table 1). Collectively, these findings emphasise the critical alterations in thermal properties conferred by specific ACTN2 variants in different domains, providing compelling evidence for the need to consider such high-throughput workflows for robust pathogenicity assessment.

**Table 1:** Thermal denaturation temperatures determined using differential scanning fluorimetry, based on fluorescence intensity ratios (350 nm/330 nm)

		Tm_0_ (°C)	Tm_1_ (°C)	ΔTm_1_ (°C)	Tm shift
	WT	-	57.0	-	-
ABD	**M92V**	**-**	**53.0**	**−4.0**	**Moderate**
	**R93Q**	**50.9**	**52.4**	**−4.6**	**Moderate**
	**G111V**	**45.5**	**50.2**	**−6.8**	**Extreme**
	**S147L**	**-**	**56.7**	**−0.3**	**No**
	**T247M**	**45.5**	**46.9**	**−10.1**	**Extreme**
Rod	D325Y	-	57.0	0.0	No
	**R327C**	**-**	**52.9**	**−4.1**	**Moderate**
	R353W	-	57.0	0.0	No
	R398H	-	58.1	+1.1	No
	**E448A**	**-**	**55.1**	**−1.9**	**Slight**
	E448K	-	56.7	−0.3	No
	**R457C**	**-**	**55.8**	**−1.2**	**Slight**
	R572W	-	57.8	+0.8	No
	E628G	-	56.0	−1.0	No
	**I653T**	**-**	**55.0**	**−2.0**	**Slight**
CaM	R759T	-	56.5	−0.5	No
	R796C	-	56.9	−0.1	No

Data represent mean values from two independent experimental runs. Tm_0_ represents the onset of protein unfolding, while Tm_1_ correspond to inflection point. ΔTm_1_ denotes the temperature shifts relative to WT. Variants were grouped into three classifications according to ΔTm_1_: Extreme (ΔTm_1_ > 6 °C), Moderate (ΔTm_1_ ≃ 4 °C), and Slight (ΔTm_1_ between 1 and 2 °C). Variants highlighted in bold are selected for further analysis with ΔTm_1_ > 1 °C, plus the S147L variant.

As most ABD variants exhibited substantial deviations in thermal stability relative to WT, except for S147L, all five ABD variants were selected for further analysis (Fig. 1D, Table 1). In addition, four variants from the rod domain, which demonstrated moderate to slight reductions in ΔTm_1_, were also selected for follow-up studies (Fig. 1D, Table 1).

### Predicting the structural effects of HCM-associated variants on ACTN2

As the nine selected variants span distinct functional regions of ACTN2, this enabled domain-specific investigations of their potential mechanistic impact. Recent cryo-EM structures of ABD-containing proteins bound to actin identify homologues with domain architectures similar to ACTN2, providing a structural framework for assessing conservation of disease-associated residues. Sequence comparisons revealed that many residues corresponding to HCM-associated variants are not conserved across homologues such as Utrophin, Filamin A, and β-III spectrin (Fig. S2). Because these variants are linked with human disease, comparative modelling of WT and mutant ACTN2 structures was performed to assess their domain-specific structural impact.

#### Structural modelling of the ACTN2/actin complex

Given that five variants localise to the ABD, we specifically examined their effects on domain structure and actin engagement. A structural model of the ACTN2 calponin homology domain 1 (CH1) in complex with actin was generated using the HADDOCK docking platform^15^ (see supplementary methods). Docking restraints were derived from the experimentally resolved cryo-EM structure of the Utrophin-ABD/actin complex (PDB ID: 6M5G)^16^, which shares significant structural homology with ACTN2-ABD (PDB ID: 5A38)^10^. Docking simulations were performed with ACTN2-CH1, as previous studies of actin complexes with Utrophin, Filamin A and β-III spectrin demonstrated that the CH2 domain does not directly participate in actin docking^16–19^. Analysis of the ACTN2-CH1/actin docking model identified three putative actin-binding interfaces consistent with those observed in the homologous proteins, with conserved ACTN2 residues predicted to bind actin (Figs. S2 and S3A), reinforcing the validity of the proposed docking orientation. Comparative modelling of WT and mutant structures was then performed to assess whether HCM-associated variants compromised actin-binding capacity or ABD conformation.

#### M92V and R93Q variants are predicted to disrupt actin engagement

Within the HADDOCK-derived ACTN2-CH1/actin model, the M92V and R93Q variants are positioned at the predicted actin-binding interface, suggesting a potential direct impact on ACTN2-actin interactions (Fig. S3B). M92 is a hydrophobic residue with an extended side-chain that projects from a loop within the CH1 domain and is predicted to form multiple contacts with I345 of actin (Fig. S4A, left panel). Substitution with the smaller valine residue is expected to reduce the extent of hydrophobic interactions at this interface, and potentially impair actin binding (Fig. S4A, right panel). In contrast, the R93Q variant is predicted to disrupt several key interactions, including a hydrogen bond with T148 of actin, a salt bridge with E167 of actin, and an intramolecular salt bridge with D124 of ACTN2 (Fig. S4B, left panel). Replacement with the smaller Q93 is likely to abolish these interactions, thereby potentially destabilising the ACTN2/actin complex (Fig. S4B, right panel).

#### G111V, S147L and T247M variants are predicted to destabilise ACTN2-ABD conformational regulation

**Fig. 2:**
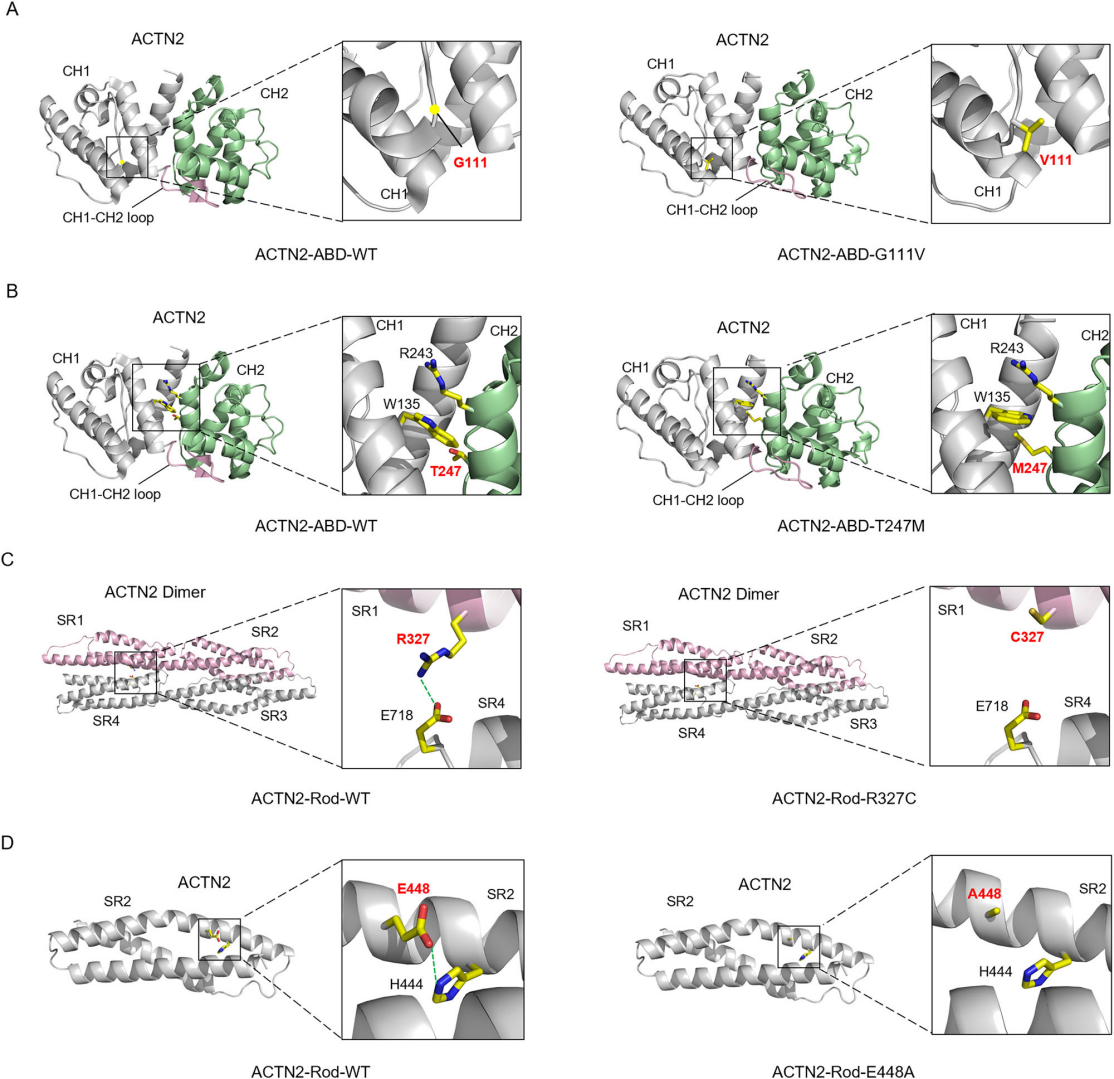
Probing the impact of the ACTN2-ABD variants (G11V and T247M), and ACTN2-Rod variants (R327C and E448A) on ACTN2 structure. **A** Schematic representation of ACTN2-ABD model showing that G111 maps to a loop region (left panel). Introduction of V111 is predicted to disrupt the loop conformation (right panel). **B** T247 side chain is near W135, which forms stacking interactions with R243 (left panel). Introduction of M247 is predicted to disrupt the W135/R243 stacking interaction (right panel). **C** Ribbon diagram illustrating that the ACTN2 dimer interface is stabilised by a salt-bridge mediated by R327 of monomer A (light pink) and E718 of monomer B (grey) (left panel). Introduction of the C327 variant is predicted to disrupt this interaction, potentially destabilising the dimer interface (right panel). **D** Ribbon diagram of ACTN2 illustrating that E448 forms a hydrogen bond with H444 (left panel). Introduction of A448 leads to the loss of this interaction and potentially destabilises the SR2 region (right panel). For clarity, specific protein structural domains are displayed. Substituted residues are highlighted in red. Green dashed line illustrates a salt bridge (**C**) or hydrogen bond (**D**) interaction. Boxes highlight close-up views of relevant interactions.

Mapping of the G111V, S147L and T247M variants onto the ACTN2-ABD indicates that these residues are positioned distal to the predicted actin-binding interface, suggesting that their effects are unlikely to arise from direct disruption of actin contact sites (Fig. S3B, C). Our HADDOCK-derived ACTN2-CH1/actin model demonstrates that the closed ABD conformation is incompatible with actin binding due to steric clash of the CH2 domain with actin (Fig. S3C). This is consistent with structural data of other CH1-containing ABD/actin complexes^16–19^, which require CH2 displacement for CH1-mediated actin engagement. G111 lies within a loop connecting two helices of the CH1 domain, and substitution with the bulkier V111 may reduce local flexibility or alter loop positioning, potentially hindering the conformational transition necessary for actin binding (Fig. 2A). Similarly, S147 resides within the inter-domain loop connecting the CH1 and CH2 domains, a region also may contribute to the conformational flexibility required for CH1-CH2 rearrangement (Fig. S4C, left panel). The introduction of the nonpolar leucine may reduce loop flexibility and restrict inter-domain movement (Fig. S4C, right panel).

The T247M variant is located at the CH1/CH2 domain interface, where T247 is positioned in close proximity to W135, a residue shown in other alpha-actinin isoforms to facilitate transitions between the open and closed ABD states via stacking interactions with R243^10,20,21^ (Fig. 2B, left panel). Substitution with the longer M247 residue could disrupt this stacking arrangement, thereby potentially interfering with the conformational dynamics that regulate actin-binding (Fig. 2B, right panel). Collectively, the G111V, S147L and T147M variants are predicted to impair the coordinated transitions between open and closed ABD conformations, thereby indirectly compromising ACTN2/actin interactions.

#### R327C, R457C, E448A, and I653T variants are predicted to destabilise the rod domain

Another predicted mechanism by which HCM-linked ACTN2 variants may contribute to disease pathogenesis is through disruption of dimer assembly. Interestingly, the R327C and R457C variants align with key dimerisation interfaces, suggesting a potential role in destabilising the ACTN2 dimer. Molecular modelling revealed that R327, situated within the SR1 region of ACTN2 (monomer A), forms a salt-bridge with E718 located in the SR4 region of monomer B (Fig. 2C, left panel). Substitution with C327, a shorter and uncharged residue, is anticipated to abolish this interaction (Fig. 2C, right panel). Likewise, R457 extends from SR2 of monomer A to form a salt-bridge with E537 in SR3 of monomer B (Fig. S4D, left panel), an interaction likely disrupted by the R457C substitution (Fig. S4D, right panel).

Two additional variants, while not directly contributing to ligand binding or dimerisation, may compromise protein stability. E448, a solvent-exposed residue projecting from SR2, forms a hydrogen bond with H444, contributing to SR2 structural integrity (Fig. 2D, left panel). Its replacement with A448 is predicted to abolish this interaction, potentially destabilising the domain (Fig. 2D, right panel). In SR4, I653 plays a role in maintaining hydrophobic core integrity through interactions with I721 and L728 (Fig. S4E, left panel). Introduction of the polar T653 in this hydrophobic environment may disrupt these interactions and invoke structural vulnerabilities within SR4 (Fig. S4E, right panel).

#### Summary of variant-induced ACTN2 structural changes

Together, these modelling data suggest that multiple ACTN2 variants may impair protein function through distinct but converging mechanisms (Table S2). Specifically, several variants may disrupt actin binding or perturb the ABD regulatory function, while others appear to compromise the structural stability of the rod domain, including dimer integrity. Overall, structural destabilisation emerges as a unifying pathogenic mechanism across the analysed ACTN2 variants. These predictions formed the basis for subsequent functional and biophysical investigations.

### R327C and R457C variants reveal no impact on ACTN2 dimer integrity

**Fig. 3:**
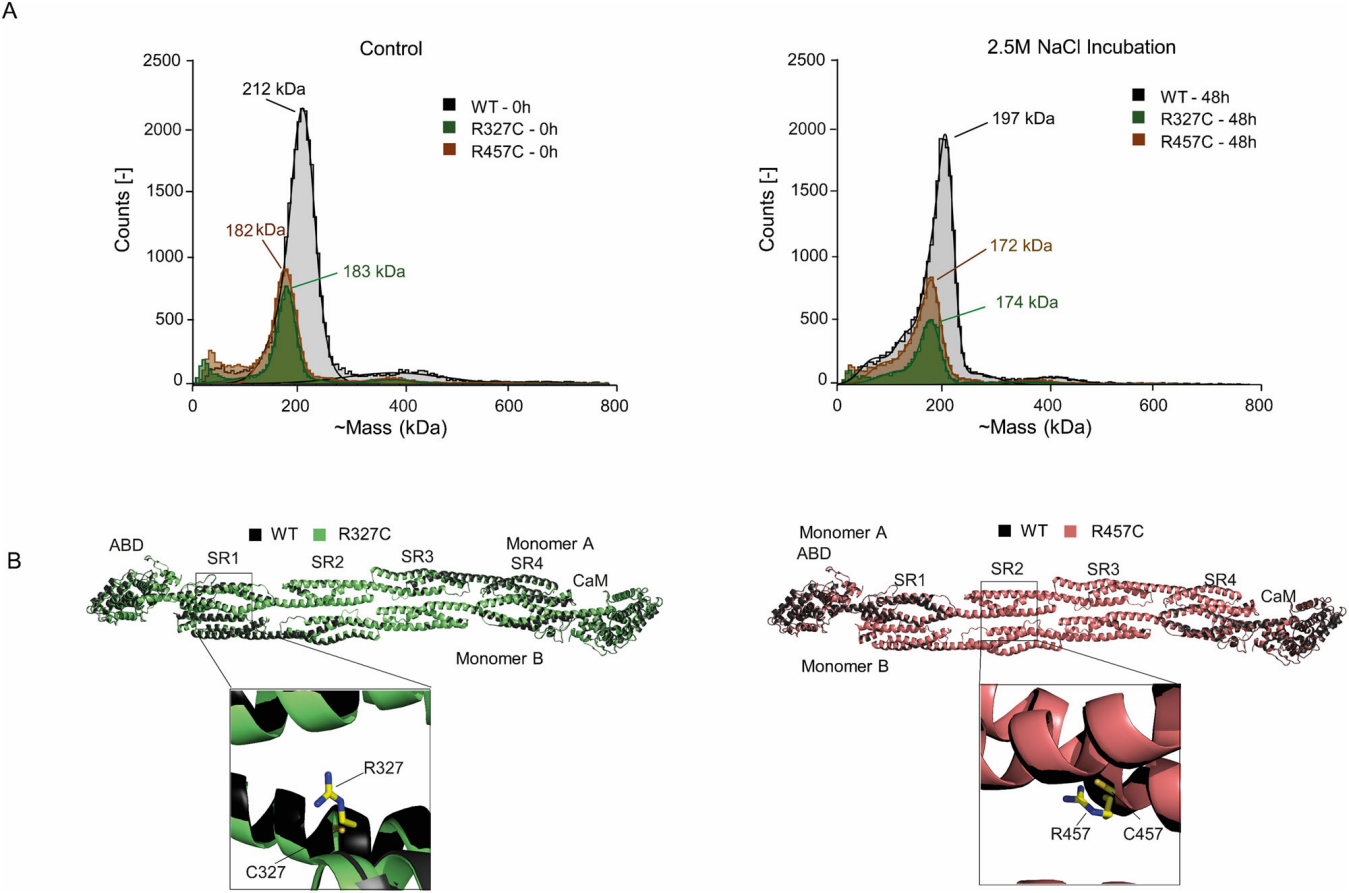
Assessment of ACTN2 dimer integrity in ACTN2-R327C and ACTN2-R457C variants. **A** The estimated molecular weights of WT, R327C, and R457C at time zero (0 h) are assessed using mass photometer (Refeyn), revealing that all samples correspond to the dimer peak (~ 200 kDa) (left panel). After 48 h of incubation with 2.5 M NaCl and rigorous shaking, reassessment of the molecular weights shows no changes across the three samples (right panel). Data presented from one independent experimental run with histograms overlayed with the peaks fit by Gaussian curves. **B** Crystal structures of the ACTN2-R327C (PDB ID: 9SIR) and ACTN2-R457C (PDB ID: 9SIS) variants. Superimposition of the ACTN2-WT crystal structure (PDB ID: 4D1E)^24^ and variant protein structures reveals high structural similarity, with a preserved dimer interface; WT (in black), R327C (in green, left panel), R47C (in brown, right panel); with boxes highlighting close-up views. Table S3 presents a summary of data collection and refinement statistics of R327C and R457C from several collected crystals.

To test our structural modelling predictions for rod-domain R327C and R457C variants, we subjected both mutant proteins and WT to high ionic strength (2.5 M NaCl) combined with mechanical stress by rigorous shaking for 48 h (Fig. 3A). Mass photometry measurements at the initial time point revealed no change in molecular weight (~ 200 kDa) for either R327C or R457C variant compared to WT (Fig. 3A, left panel). Furthermore, repeated measurements after 48 h of rigorous shaking showed no detectable differences in molecular weight (~ 200 kDa), suggesting that dimerisation remains intact even under combined ionic and mechanical stress (Fig. 3A, right panel).

To further define the structural impact of rod-domain variants, we attempted crystallisation of several ACTN2 rod domain constructs harbouring these substitutions. Of these, only two yielded diffraction-grade crystals, enabling structure determination of ACTN2-R327C (PDB ID: 9SIR) and ACTN2-R457C (PDB ID: 9SIS), both at 3.7 Å maximum resolution. The resulting structures showed no detectable disruption in dimer formation (Fig. 3B), consistent with the mass photometry data. Structural superimposition of each variant onto the WT (PDB ID: 4D1E) revealed no significant conformational differences, with a root mean square deviation values of 0.476 Å (851 aligned Cα atoms) and 0.471 Å (859 aligned Cα atoms), respectively, indicating a highly similar overall topology (Table S3). These results collectively highlight that the cysteine substitutions are not sufficient to compromise overall ACTN2 dimer integrity, suggesting that alternative structural or regulatory mechanisms may underlie the pathogenic effects of these variants.

### ACTN2 HCM-linked missense variants affect actin-binding

**Fig. 4:**
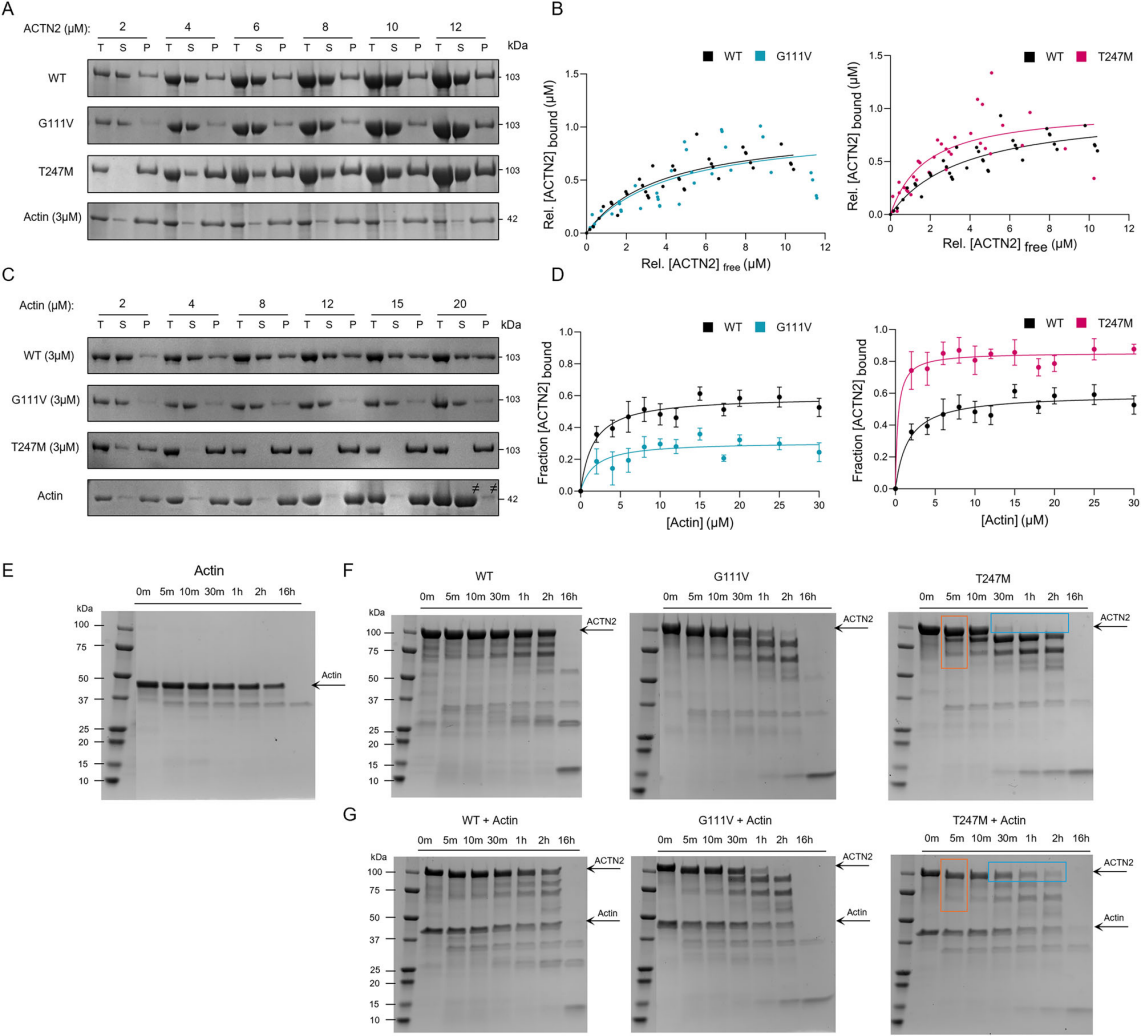
Assessment of the impact of ACTN2 variants on actin binding. **A**, **B** Actin binding assays evaluating the impact of ACTN2-ABD variants on actin-binding affinity and capacity. Rabbit skeletal muscle actin (3 μM) was co-sedimented with varying concentrations of the wild type (WT) and the variant proteins G111V and T247M (0.25–12 μM). **A** Representative gels of WT, G111V, T247M, and actin. **B** (left panel) G111V variant show no change in binding affinity. **B** (right panel) T247M variant shows an increase in binding affinity. A non-linear regression analysis was used with each data points representing an independent co-sedimentation. Graphs display measurements after normalisation to the maximum binding capacity (B_max_) values of each variant. Representative graphs for variants M92V, R93Q and S147L are displayed in Fig. S5. **C** Representative gels of reciprocal actin binding assay performed using 3 μM of WT, G111V or T247M, co-sedimented with increasing concentrations of actin (0–30 μM). ≠ denotes a swap of actin supernatant and pellet samples at 20 μM at the point of gel loading. **D** G111V variant shows an observable decrease in binding, while T247M shows an increase (left and right panels, respectively). A non-linear regression analysis was used with 4 independent co-sedimentations per actin concentration (except for values at actin concentrations of 6, 8 and 18 μM with only 3 independent replicates). Values presented with error bars indicating standard deviation (SD). Table S4 lists B_max_ and K_d_ values for each variant with corresponding confidence intervals and *p* values. Additional representative gels for (**A**, **C**) are presented in Fig. S6. T total protein, S supernatant, P pellet. **E**, **F** Proteolytic digestion of actin, ACTN2-WT, G111V, or T247M using thermolysin at 37 °C, respectively with an enzyme-to-substrate molar ratio of 1:7. **G** Proteolytic digestion of WT, G111V, or T247M with actin with a molar ratio of 1:7:7 (Thermolysin : ACTN2 : Actin). Reactions were terminated at specific time points. T247M digestion is displayed in boxes (orange [5 min]; blue [30 min to 2 h]). Molecular weight markers (kDa) are indicated. Data presented from one independent experimental run. Proteolytic digestion of other ACTN2 variants are displayed in Fig. S7.

To test the modelling predictions of impaired ABD function, the functional impact of five ACTN2-ABD variants on actin binding was assessed using actin co-sedimentation assays with fixed actin and increasing ACTN2 concentrations. The binding affinity constant (K_d_) and the maximum binding capacity (B_max_) were determined, representing the strength and extent of actin interaction, respectively. The M92V variant exhibited an increased K_d_ compared to WT (WT K_d_ = 3.75 µM, M92V K_d_ = 13.4 µM), indicating an overall reduction in actin-binding affinity, while R93Q displayed no changes (Fig. S5A, B, Table S4). In contrast, S147L showed increased binding affinity but reduced binding capacity (Fig. S5C, Table S4). G111V displayed no change in binding affinity but exhibited a decrease in binding capacity, while T247M demonstrated increases in both affinity and capacity (Figs. 4A, B and S6A, Table S4). To further characterise the effects observed for G111V and T247M, reciprocal co-sedimentation assays were performed using fixed ACTN2 and increasing actin concentrations. Under these conditions, G111V exhibited an observable decrease in actin binding as evidenced by the substantial deviation from the WT fit curve, along with a reduction in binding capacity (Fig. 4C, D [left panel], S6B, Table S4). Analysis of the T247M variant again showed increased binding affinity and capacity (Figs. 4C, D [right panel] and S6B, Table S4).

### Thermolysin digestion provides insights for structural stability

To further evaluate the functional changes observed in actin binding assays, we sought to determine whether altered interactions between the ACTN2-ABD and actin influence domain stability. As missense variants can compromise protein folding and reduce structural stability^22^, limited proteolysis with thermolysin provides a sensitive means for probing such changes. As thermolysin preferentially cleaves at hydrophobic residues, regions that become more flexible or exposed upon unfolding are more readily digested, whereas stable regions remain resistant.

We first assessed the conformational stability of actin, which remained stable for up to 2 h (Fig. 4E). ACTN2 WT also remained highly stable even after 2 h of incubation, with no change in digestion profile in the presence of actin (Fig. 4F, G, left panels). G111V showed accelerated degradation when incubated alone (within 30 min), and this profile was unchanged in the presence of actin (Fig. 4F, G, middle panels). Notably, the T247M variant underwent rapid degradation within 5 min when incubated alone (Fig. 4F right panel). However, in the presence of actin, T247M revealed clear actin-dependent stabilisation, remaining stable for up to 30 min and exhibiting increased resistance to proteolysis even after 2 h (Fig. 4G, right panel). This suggests that enhanced actin binding, likely mediated through engagement of the CH1-CH2 interface, may promote burial of exposed hydrophobic surfaces, thereby delaying proteolytic digestion and partially neutralising the destabilising effect of thermolysin. We next evaluated whether other ABD and rod variants exhibited altered stability in the presence of thermolysin (Fig. S7). The ABD variants M92V, R93Q and S147L degraded at rates similar to WT (Fig. S7A, B). Interestingly, after 16 h of incubation, R93Q and S147L variants produced displayed higher molecular weight fragments than WT, potentially reflecting reduced susceptibility to complete digestion. A similar pattern was observed for rod domain variants, R327C and E448A, which largely retained stability relative to WT but also generated additional higher molecular weight fragments (Fig. S7C). In contrast, the digestion profiles of R457C and I653T were indistinguishable from WT (Fig. S7D).

Overall, ABD variants, particularly G111V and T247M, were more prone to structural destabilisation than those in the rod domain, indicating greater conformational vulnerability within this region. Strikingly, enhanced actin binding in T247M appeared to partially mitigate this destabilisation.

### ACTN2 variants display increased propensity to aggregate formation using SEC-MALS

**Fig. 5:**
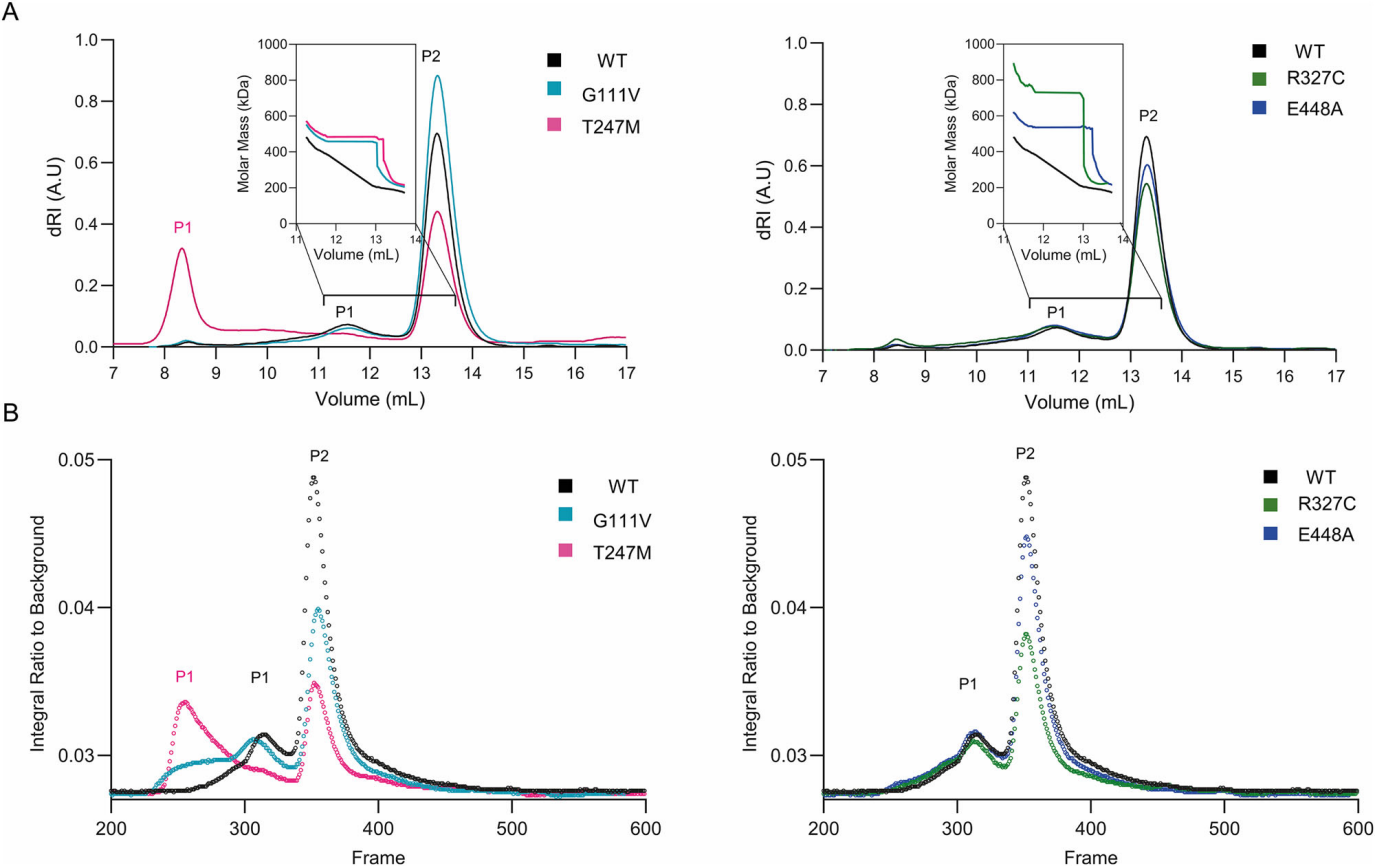
Assessment of structural stability of ACTN2 variants. **A** Size exclusion chromatography coupled with multi-angle light scattering (SEC-MALS) analysis showing differential refractive index (dRI) profiles eluting in two peaks (P1-P2) with upper right insets displaying molar mass (kDa) of WT and ACTN2 variants (G111V, T247M, left panel; R327C, E448A, right panel). Similar analysis for variants M92V, R93Q, R457C, and I653T are displayed in Fig. S8. SEC-MALS chromatograms of UV absorbance and light scattering are displayed in Fig. S9. **B** Size exclusion chromatography coupled with small-angle X-ray scattering (SEC-SAXS) signal plot showing protein eluting as two peaks (P1-P2) with each point representing the integrated area of the ratio of the sample SAXS curve to the estimated background. The first peak (P1) of ACTN2 variants shows aggregate formation compared to WT. T247M-P1 are referred to in pink in SEC-MALS and SEX-SAXS traces. The frames selected for buffer extraction and peak elution are displayed in Table S5 and Fig. S10. Log_10_ SAXS plots and Guinier analysis of P1 and P2 are displayed in Figs. S11 and S12. DENSS-generated electron density models of P2 are displayed in Fig. S13. Data presented for SEC-MALS and SEC-SAXS are from one independent experimental run, with data summarised in Table 2.

Given that thermolysin proteolysis assay highlighted particular ACTN2-ABD variants as structurally destabilised, we next investigated whether this reduced stability altered the aggregation propensity of ACTN2 variants in solution. Size-exclusion chromatography coupled with multi-angle light scattering (SEC-MALS) was used (Figs. 5A, S8A and S9, Table 2), and the WT protein eluted into two peaks (P1 and P2) (Fig. 5A, Table 2). Peak 1 of WT protein displayed a molecular weight (MW ~ 400 kDa) and a hydrodynamic radius (R_H_ ~ 10 nm), while P2 exhibited a MW ~ 200 kDa and R_H_ of ~7 nm, the latter corresponding to the physiologically relevant ACTN2 dimer (Fig. 5A, Table 2). The particles of both WT and mutant protein in P1 and P2 were homogeneous, with a poly-dispersity index (PDI) close to 1.00 (Table 2).

**Table 2:** ACTN2 variants showing aggregate formation using SEC-MALS and SEC-SAXS

				ABD				Rod				
			WT	M92V	R93Q	G111V	S147L	T247M	R327C	E448A	R457C	I653T
(A) SEC-MALS	Peak 1 (P1)	MW (kDa)	421 ± 3.7	486 ± 3.8	514 ± 2.0	515 ± 1.9	320 ± 1.5	525 ± 2.6	800 ± 14.4	603 ± 7.2	729 ± 10.2	411 ± 2.4
		R_H_ (nm)	10.4 ± 0.05	10.0 ± 0.05	10.6 ± 0.04	10.0 ± 0.06	9.6 ± 0.05	11.7 ± 0.08	16.8 ± 0.10	12.4 ± 0.05	16.8 ± 0.08	9.6 ± 0.07
		PDI [-]	1.002	1.002	1.005	1.008	1.009	1.005	1.005	1.008	1.007	1.010
	Peak 2 (P2)	MW (kDa)	193 ± 0.7	204 ± 0.6	202 ± 0.8	213 ± 1.1	185 ± 0.8	217 ± 0.6	232 ± 2.5	221 ± 1.3	222 ± 1.5	249 ± 1.0
		R_H_ (nm)	7.4 ± 0.02	7.3 ± 0.02	7.4 ± 0.02	7.0 ± 0.02	7.4 ± 0.03	7.6 ± 0.03	8.6 ± 0.03	7.8 ± 0.02	8.6 ± 0.03	7.9 ± 0.02
		PDI [-]	1.002	1.002	1.005	1.009	1.008	1.005	1.005	1.008	1.007	1.010
(B) SEC-SAXS	Peak 1 (P1)	*R*_*g*_*(Guinier)* (Å)	120	N/D	N/D	N/D	-	N/D	N/D	N/D	N/D	-
		*R*_*g*_*(real)* (Å)	125	157	180	175	-	264	157	153	159	-
		*D*_*max*_ (Å)	410	490	570	555	-	780	489	481	499	-
		*V* (10^3^ Å^3^)	865	1086	1509	1648	-	2688	1253	1125	1135	-
		MW (kDa)	521	654	909	992	-	1619	754	677	683	-
	Peak 2 (P2)	*R*_*g*_*(Guinier)* (Å)	107	105	107	117	-	118	110	116	113	-
		*R*_*g*_*(real)* (Å)	112	112	113	116	-	117	114	114	112	-
		*D*_*max*_ (Å)	362	364	370	369	-	369	360	365	370	-
		*V* (10^3^ Å^3^)	389	372	408	474	-	457	418	443	379	-
		MW (kDa)	234	224	245	285	-	275	251	266	228	-

(A) Size-exclusion chromatography coupled with multi-angle light scattering (SEC-MALS) assessment of peaks 1 and 2 showing different parameters of WT and ACNT2 variants including molecular weight (MW), hydrodynamic radius (R_H_), and poly-dispersity index (PDI). Values are presented as measured values ± uncertainty. (B) Size-exclusion chromatography coupled with small-angle X-ray scattering (SEC-SAXS) showing values such as radius of gyration with Guinier analysis [*R*_*g*_*(Guinier)*], real space radius of gyration [*R*_*g*_*(real)*], maximum particle dimension *(D*_*max*_*)*, particle volume (*V*) and estimated molecular weight (MW) for both peaks (P1 and P2). *R*_*g*_*(Guinier)* values for P1 in ACTN2 variants are undetermined (N/D) as data deviate from the Guinier approximation of (*q.Rg* < 1.3). All variants except for S147L and I653T are selected for further analysis after showing aggregate formation. Data presented for SEC-MALS and SEC-SAXS are from one independent experimental run.

While the dimer peak (P2) was detectable for all variants and comparable to WT, variants exhibited differences in higher order multimers (P1). ABD variants including M92V, R93Q, G111V, and T247M showed an increased propensity for aggregate formation, reflected by higher MW (~ 500 kDa) in P1. However, their R_H_ values were comparable to WT, except for T247M, which showed a slight increase in R_H_ (~ 12 nm) (Figs. 5A and S8A, Table 2). The rod domain variants R327C, R457C, and E448A exhibited more pronounced effects, with P1 MW ranging from 600 to 800 kDa and R_H_ values extending to 12–16 nm, consistent with larger aggregates. In contrast, the S147L and I653T variants did not display aggregate formation, with MW and R_H_ values comparable to WT (Fig. S8A, Table 2). These findings suggest that for some ACTN2 variants, conformational destabilisation is accompanied by an increased tendency to form higher-order aggregates, which may further compromise folding and stability.

### ACTN2 variants display aggregate formation using SEC-SAXS

The ACTN2 variants that exhibited aggregate formation were further characterised using size-exclusion chromatography coupled with small-angle X-ray scattering (SEC-SAXS) (Figs. 5B, S8B and S10–S13, Table 2). The SEC-SAXS elution profile of ACTN2 WT revealed two distinct peaks, consistent with the previous SEC-MALS data (Figs. 5A, S8A and S10, Table 2). While the second peak (P2) yielded parameters comparable to the ACTN2 dimer, the first peak (P1) in WT corresponded to discrete large multimers (Table 2). Analysis of P1 gave a radius of gyration of 120 Å from Guinier analysis *[R*_*g*_*(Guinier)]*, a real-space radius of gyration *[R*_*g*_*(real)]* of 125 Å, a maximum particle dimension *(D*_*max*_*)* of 410 Å, a particle volume *(V)* of 865 × 10^3^ Å^3^, and an estimated molecular weight (MW) of 410 kDa (Figs. 5B and S11, Table 2). Notably, the log_10_ SAXS profile of T247M-P1 displayed a shift at low *q* compared to WT (Fig. S11A, E, left panel). The *R*_*g*_*(Guinier)* of T247M-P1 was undetermined due to deviation from the Guiner linearity (*q.R*_*g*_ < 1.3), suggesting aggregate formation (Fig. S11E, middle panel). Consistent with this, T247M displayed increased parameters including *R*_*g*_*(real)* of 275 Å, *D*_*max*_ of 780 Å, *V* of 2688 × 10^3^ Å^3^, and MW of 1581 kDa (Figs. 5B and S11E, Table 2). Other ABD variants (M92V, R93Q, G111V) demonstrated increases similar to T247M (Figs. 5B, S8B and S11, Table 2). For these variants, P1 showed no change in log_10_ SAXS profiles but a shift in the Guiner linearity (Fig. S11B, C, D, left and middle panels). Rod-domain variants (R327C, E448A, R457C) also exhibited increased parameters indicative of aggregation (Figs. 5B, S8B and S12, Table 2). In contrast, P2 yielded parameters comparable to the WT dimer across all variants (Table 2). Ab initio SAXS electron density reconstructions generated with DENSS^23^ confirmed these findings, with P2-derived models closely resembling the ACTN2 dimer crystal structure (Fig. S13).

### ACTN2 variants display structural instability at elevated temperatures

**Fig. 6:**
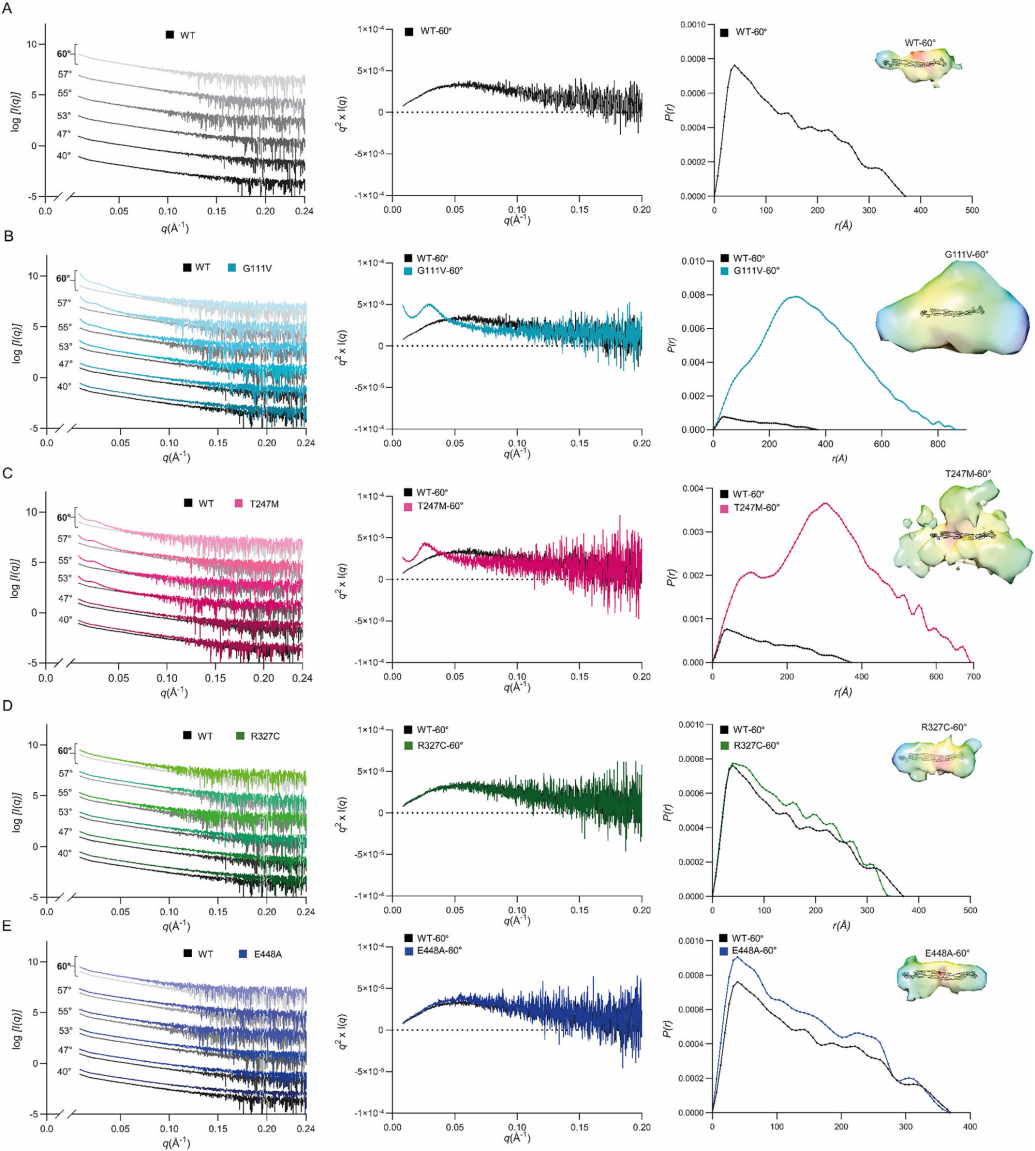
Assessment of thermal stability of ACTN2 variants using small-angle X-ray scattering (SAXS) at increasing temperature. **A** (left panel) ACTN2 WT log_10_ SAXS intensity versus scattering vector *(q)* at temperatures ranging from 40 to 60 °C; plotted range represents positive-only data within the specified *q*-range. **A** (middle panel) Kratky analysis of ACTN2 WT; plotted range represents the product of *q²* and I*(q)* versus scattering vector *(q)*. **A** (right panel) Pair-distance *P(r)* distribution function of WT at 60 °C showing the maximum particle dimension *(D*_*max*_*)*, defined as the largest non-negative value supporting a smooth distribution function; upper right inserts display SAXS electron density model generated using DENSS (3D colourful transparent volume), with ACTN2 X-ray structure (PDB ID: 4D1E)^24^ as a ribbon diagram in black. **B**, **C** Variants G111V and T247M display shifts in log_10_ SAXS plots between 53 and 60 °C (left panel), shift in Kratky plots (middle panel), and increased *D*_*max*_ (right panel), with insets (upper right) displaying aggregation in DENSS models. **D**, **E** Variants R327C and E448A show no changes in log_10_ plots (left panel), Kratky plots (middle panel), or *P(r)* distribution (right panel) compared to WT; insets (upper right) show DENSS models with no aggregation. Similar analyses for variants M92V, R93Q, and R457C are displayed in Fig. S14. Figures S15–S18 display the Guinier analysis at 40, 53, and 60 °C; Kratky analysis, *P(r)* distributions, DENSS models at 40 and 53 °C, and FoXS analysis at 40, 53 and 60 °C for ABD variants. Figures S19–S22 display similar analyses for rod variants. Data presented are from one independent experimental run at each temperature, with data summarised in Table 3.

Since SEC-SAXS revealed substantial aggregate formation in all selected ACTN2 variants, we next assessed their thermal stability using batch-mode SAXS (Figs. 6 and S14–S22, Table 3). These variants had also previously demonstrated impaired stability by DSF (Fig. 1D, Table 1). To minimise interference from aggregates, all samples were initially passed through a size-exclusion chromatography column, and only fractions corresponding to the dimer peak were used for analysis.

**Table 3:** Summary of small angle X-ray scattering (SAXS) data collected in batch-mode across temperatures ranging from 40 to 60 °C for WT, and variants in the ABD and rod domains

			ABD				Rod		
	Temperature (°C)	WT	M92V	R93Q	G111V	T247M	R327C	E448A	R457C
*R*_*g*_*(Guinier)* (Å)	40	108	105	108	116	123	113	110	103
	47	105	107	108	112	126	108	109	103
	53	103	103	106	N/D	N/D	103	106	102
	55	108	117	108	N/D	N/D	123	113	104
	57	105	108	114	N/D	N/D	113	115	107
	60	110	N/D	N/D	N/D	N/D	104	110	115
*R*_*g*_*(real)* (Å)	40	113	114	112	120	120	115	121	120
	47	111	110	111	114	130	114	122	108
	53	112	110	111	**145 ↑**	**226 ↑↑**	112	121	109
	55	112	115	113	**189 ↑**	**219 ↑↑**	113	114	111
	57	113	115	115	**205 ↑↑**	**224 ↑↑**	114	113	112
	60	116	**177 ↑**	**169 ↑**	**243 ↑↑**	**240 ↑↑**	113	114	113
*D*_*max*_ (Å)	40	377	383	371	386	379	370	380	370
	47	371	350	372	370	404	360	380	341
	53	377	350	360	**430 ↑**	**670 ↑↑**	360	360	353
	55	366	360	350	**550 ↑**	**633 ↑↑**	347	358	350
	57	370	360	360	**700↑↑**	**630 ↑↑**	370	370	360
	60	370	**468 ↑**	**492 ↑**	**870 ↑↑**	**701 ↑↑**	340	369	370
*V* (10^3^ Å^3^)	40	406	402	396	487	594	455	421	391
	47	387	401	408	471	664	433	399	390
	53	403	423	419	**894 ↑**	**2396 ↑↑**	433	417	410
	55	495	545	469	**1668 ↑**	**2900 ↑↑**	599	501	471
	57	445	513	580	**4124 ↑↑**	**2244 ↑↑**	527	465	451
	60	567	**1472 ↑**	**1570 ↑**	**6685 ↑↑**	**3895 ↑↑**	682	554	684
MW (kDa)	40	244	242	239	293	358	274	253	235
	47	233	242	246	284	400	261	240	235
	53	242	255	252	**538 ↑**	**1443 ↑↑**	260	251	247
	55	298	328	282	**1005 ↑**	**1747 ↑↑**	361	302	283
	57	268	309	349	**2484 ↑↑**	**1352 ↑↑**	317	280	272
	60	341	**886 ↑**	**945 ↑**	**4027 ↑↑**	**2346 ↑↑**	411	333	412
χ² Fit [-]	40	0.96	0.83	1.28	1.43	1.27	1.66	0.87	0.85
	47	0.93	0.90	1.37	1.35	1.74	1.70	1.09	0.89
	53	1.07	1.24	1.45	**4.84 ↑**	**12.97 ↑↑**	1.30	1.16	1.01
	55	0.81	1.05	1.08	**7.50 ↑**	**16.24 ↑↑**	1.08	0.99	0.75
	57	1.53	2.10	2.93	**36.10 ↑↑**	**15.97 ↑↑**	1.89	0.67	1.41
	60	2.77	**10.84 ↑**	**19.93 ↑**	**60.91 ↑↑**	**22.69 ↑↑**	4.34	3.29	3.98

Parameters include the radius of gyration by Guinier analysis *[R*_*g*_*(Guinier)]*, real-space radius of gyration *[R*_*g*_*(real)]*, maximum particle dimension *(D*_*max*_*)*, estimated particle volume *(V)*, estimated molecular weight (MW). χ² (chi-squared) values are obtained from FoXS (Fast X-ray Scattering) analysis^47^. *R*_*g*_ *(Guinier)* values for some ACTN2 variants are undetermined (N/D) as the scattering data deviate from the Guinier approximation of (*q.R*_*g*_ < 1.3). The symbol (**↑**) denotes an increase relative to WT, with corresponding values highlighted in bold. Guinier analysis, representative *P(r)* distributions illustrating *D*_*max*_, DENSS models, and χ² FoXS fits at 40, 53, and 60 °C for WT and variants are provided in the supplementary material (Figs. S11–S22). Data presented for batch-mode SAXS are from one independent experimental run per each measured temperature.

SAXS measurements were performed at six temperatures (ranging from 40 to 60 °C) and log_10_ scattering profiles, Guinier analysis, Kratky analysis, pair-distance *P(r)* distribution functions, and DENSS reconstructions were determined. FoXS server was also used for fitting SAXS profiles to ACTN2 experimental data. ACTN2 WT showed no appreciable changes in the log_10_ plots across the tested range; even at 60 °C, and the data fitted to the Guinier linearity (*q.R*_*g*_ < 1.3) (Figs. 6A and S15, Table 3). *P(r)* distribution yielded a *D*_*max*_ of 370 Å (Figs. 6A and S17), consistent with values reported in previous SAXS analyses of ACTN2 protein^24^. FoXS analysis revealed a consistent fitting between SAXS profile and experimental data (Fig. S18, Table 3).

In contrast to the WT protein, the ABD variants G111V and T247M displayed marked structural transitions between 53 to 60 °C. These variants exhibited pronounced shifts in log_10_ scattering profiles (Fig. 6B, C [left panel], Table 3), and a systematic deviation from Guinier linearity (*q.R*_*g*_ > 1.3) at these elevated temperatures (Fig. 6B, C [left panel], S15, Table 3). The upward divergence of data points from the best-fit line, suggest the onset of temperature-induced aggregation^25^ (Fig. S15D, E). Further characterisation via Kratky analysis (Figs. 6A, B, C [middle panel] and S16A, D, E), revelaed transition to a partualy unfolded state, corroborated by a substantial increase in *D*_*max*_, a pronounced boradening of the *P(r)* distance distrubution curves. These markers of decreased structural compactness were further visualised as disordered density in DENSS models (Figs. 6B, C [right panel] and S17A, D, E, Table 3), supported by a noticeable increase in χ² values during FoXs analysis, indicating that the experimental scattering no longer matched the folded state (Fig. S18C, D, Table 3). Other ABD variants (M92V and R93Q) exhibited structural transitions similar to G111V and T247M, however these were only evident at 60 °C (Fig. S14A, B, Table 3). At 40 and 53 °C, these variants showed no significant deviations in Guinier fitting (Fig. S15B, C), Kratky analysis (Fig. S16B, C), *P(r)* distributions or DENSS reconstructions (Fig. S17B, C), and maintained stable FoXS χ² values (Fig. S18A, B).

By contrast, rod domain variants (R327C, R457C, E448A) maintained stable scattering profiles across all temperatures tested (Figs. 6D, E and S14C, Table 3). These variants showed no evidence of aggregation or unfolding, with Guinier linearity, Kratky peak profiles, *P(r)* distributions and FoXS χ² values remaining comparable to WT (Figs. 6D, E and S14C and S19–S22).

Collectively, our batch-mode SAXS analysis reveals a clear distinction in the thermal stability of ACTN2 domains. The ABD appears to be a hotspot for structural vulnerability, particularly for G111V and T247M, which exhibit early onset destabilisation at 53 °C, and M92V and R93Q failing at 60 °C. Conversely, the rod-domain variants remain conformationally robust, suggesting that variants in the rod region are significantly better tolerated by the global protein fold. These results indicate that ABD-domain variants may confer a higher risk of structural and functional impairment due to their lower intrinsic stability compared to the rod domain.

## Discussion

ACTN2 missense variants have been implicated in hypertrophic cardiomyopathy (HCM), yet the molecular mechanisms driving their pathogenicity remain poorly defined. Building on our earlier findings^4^, we expand this work by systematically characterising ACTN2 variants using an integrated and tiered workflow. Seventeen missense variants spanning multiple domains were initially assessed through high-throughput approaches, including solubility screening and differential scanning fluorimetry (DSF), after which a subset was prioritised for in-depth functional, biophysical and structural analyses. This strategy uncovered domain-specific mechanisms of pathogenicity and established a scalable framework for variant interpretation that may be applicable to other HCM-associated genes.

Initial solubility screening in *E. coli* indicated reduced solubility for most variants. Subsequent DSF assessment revealed pronounced thermal instability of ABD variants, including R93Q, G111V, and T247M, which unfolded at markedly lower temperatures than WT. In contrast, most rod and CaM-domain variants retained thermal stability, highlighting the ABD as a structurally vulnerable region, consistent with its critical role in anchoring actin filaments. Importantly, these results extend prior observations from ABD-only constructs of G111V and T247M variants^10,11^ by confirming destabilisation in the full-length protein, thereby capturing effects in a more physiologically relevant context.

Based on DSF effect size, nine variants showing mild to large thermal shifts were selected for structural modelling to generate mechanistic hypotheses. These analyses predicted variant-specific effects such as impaired actin-binding (M92V, R93Q), disruption of ABD regulatory conformations (G111V, S147L, T247M), perturbed dimerisation (R327C, R457C), and destabilisation of rod-domain architecture (E448A, I653T). Functional and biophysical analyses were used to validate or challenge modelling-based interpretations. Analysis of rod-domain R327C and R457C variants contradicted modelling predictions by revealing intact dimers, even under high-salt conditions known to dissociate other dimeric proteins^26,27^. This resilience likely reflects extensive inter-subunit contacts within spectrin repeats^28^, suggesting that disruption of individual salt bridges is insufficient to destabilise dimer assembly, although subtle allosteric effects cannot be excluded.

Functional actin-binding assays both validated and refined modelling predictions for ABD variants, revealing mechanistic diversity. Specifically, while M92V reduced actin-binding affinity as predicted, R93Q exhibited no detectable change despite predicted impairment. In contrast, S147L increased binding affinity but reduced capacity, suggesting tighter yet less dynamic actin engagement. G111V displayed decreased binding capacity in both the actin-binding assay and its reciprocal approach, while the latter additionally indicated an observable decrease in binding. These findings align with earlier reports of reduced actin-binding affinity for G111V in ABD-only constructs^10^. T247M, by contrast, exhibited enhanced actin-binding affinity and capacity in both assay formats, consistent with previously reported kinetic data^11^ and our modelling prediction that it favours an open CH1-CH2 conformation, a key regulatory feature of ABD function^4^. Notably, similar increases in actin binding have been reported for variants at this interface in β-III spectrin^29^. Together, these findings indicate that pathogenicity in ACTN2-ABD variants may arise from either increased or reduced actin-binding, with altered binding dynamics emerging as a critical determinant of functional disruption.

Domain-specific effects of ACTN2 variants were further supported by thermolysin proteolysis assays, which probe structural flexibility through preferential cleavage at hydrophobic residues^30^. ABD variants (G111V, T247M) exhibited accelerated proteolytic digestion, consistent with enhanced structural lability. Notably, the increased actin-binding observed for T247M to actin served as a potential neutraliser for CH1-CH2 interface destabilisation by thermolysin. In contrast, rod-domain variants displayed greater resistance to digestion, indicative of compartively limited structural rearrangements. Complementary analyses using SEC-MALS and SEC-SAXS revealed that most variants were prone to aggregation, although non-aggregated fractions maintained a WT-like dimeric state. Thermal SAXS confirmed heightened vulnerability in ABD variants, particularly G111V and T247M, revealing earlier onset of temperature-induced aggregation and compromised global thermodynamic stability of the ABD. While these structural deviations occur above physiological thermal range, they serve as a sensitive ‘stress test’. Such destabilisation may increase the frequency of transient unfolding at 37 °C, potentially rendering the ABD more susceptible to proteolysis or mechanical failure under the cyclic loading conditions of the sarcomeres. Conversely, rod-variants maintained a more robust fold reflecting no detectable impact from this ‘stress test’.

Collectively, these results demonstrate that ABD variants are more susceptible to structural vulnerability, while rod-domain variants appear more resistant but remain prone to secondary perturbations. These findings also suggest that the pathogenicity of ACTN2 variants arises not only from direct domain-specific disruptions, such as altered actin-binding, but also from broader biophysical consequences including decreased solubility, thermal instability, impaired folding, and aggregration (as summarised in Table 4). Such mechanistic diversity may extend to other cytoskeletal proteins with similar domain architectures, including Utrophin, Filamins, and β-III spectrin. However, despite exhibiting comparable actin-binding modes, HCM-linked ACTN2 variants within the ABD and rod domains are not fully conserved across these homologues. This lack of conservation suggests that some residues may fulfil ACTN2-specific roles in stabilising domain organisation or modulating ligand interactions. Interestingly, three ACTN2 variants (R327C, R457C, E628G) correspond to mutation sites identified in β-III spectrin using exome sequencing studies, including R351P^31^, R480W^32^, R633Q^33^, linked to spinocerebellar ataxia, an inherited neurodegenerative disease.

**Table 4:** Summary of analyses performed for the nine selected variants in ACTN2-ABD and rod domains

Domain	ACTN2 variants	Solubility yield	DSF (Tm_1_ shift, Tm_0_ onset)	Structural modelling predictions	Dimer stabi-lity	Actin-binding assay	Reciprocal actin-binding assay	Thermolysin digestion without actin	Thermolysin digestion with actin	SEC-MALS aggregation	SEC-SAXS aggregation	SAXS batch-mode aggregation (at Temp)
ABD	M92V	**↓**	Moderate	Impairs actin binding	-	**↓** Affinity **↑** Capacity	-	No change	-	Yes	Yes	**↑** at 60 °C
	R93Q	↓****	Moderate Tm_0_ 51 °C	Impairs actin binding	-	No change	-	Retention of larger fragments	-	Yes	Yes	**↑** at 60 °C
	G111V	↓****	Extreme Tm_0_ 45 °C	Disrupts ABD conformation	-	No change in Affinity **↓** Capacity	No change in Affinity ↓ Capacity	Accelerated digestion (30 min)	Same digestion as without actin	Yes	Yes	**↑** at 53 °C, 55 °C and **↑↑** at 57 °C, 60 °C
	S147L	↓*	None	Disrupts ABD conformation	-	↑ Affinity, **↓** Capacity	-	Retention of larger fragments	-	No	-	-
	T247M	↓****	Extreme Tm_0_ 45 °C	Disrupts ABD conformation	-	**↑** Affinity **↑** Capacity	**↑** Affinity **↑** Capacity	Rapid degradation (5 min)	Digestion at 30 min and increased stability	Yes	Yes	**↑↑** at 53 °C, 55 °C, 57 °C, 60 °C
Rod	R327C	↓**	Moderate	Disrupts Dimerisation	Stable	-	-	Retention of larger fragments	-	Yes	Yes	No
	E448A	↓*	Slight	Domain instability	-	-	-	Retention of larger fragments	-	Yes	Yes	No
	R457C	↓**	Slight	Disrupts Dimerisation	Stable	-	-	No change	-	Yes	Yes	No
	I653T	↓***	Slight	Domain instability	-	-	-	No change	-	No	-	-

The symbols (↑) and (↓) denote an increase or decrease relative to WT, respectively. Solubility yield data are analysed using Ordinary One-way ANOVA with Dunnett test used for multiple comparison, and statistical significance indicated as *p < 0.05, **p < 0.01, ***p < 0.001, ****p < 0.0001. *p* values of variants vs. WT are: M92V = 0.1040, R93Q < 0.0001, G111V < 0.0001, S147L = 0.0270, T247M < 0.0001, R327C = 0.0014, E448A = 0.0140, R457C = 0.0012, I653T = 0.0001.

*ABD* actin-binding domain, *DSF* differential scanning fluorometry, *Tm*_*1*_ temperature inflection point, *Tm*_*0*_ onset of unfolding.

Beyond mechanistic insights, this study establishes a systematic, tiered workflow for variant assessment that integrates high-throughput screening with detailed structural and biophysical analyses. Rapid, resource-efficient approaches, such as conservational sequence alignment, structural modelling, solubility testing and DSF, proved effective for initial prioritisation, while resource-intensive methods, including X-ray crystallography, SEC-MALS, SEC-SAXS and thermal SAXS, provided deeper mechanistic resolution. This strategy balances throughput with analytical depth and offers a scalable framework for evaluating variants of uncertain significance in cardiomyopathy-linked genes. Importantly, our use of full-length ACTN2 protein provides a more physiologically relevant context than prior studies based on truncated proteins^10,11^, and extends analyses beyond the ABD to include variants in the rod and CaM domains. Notably, our application of thermal SAXS to monitor conformational changes and aggregation represents, to our knowledge, an important approach in the study of variant pathogenicity.

Nevertheless, several limitations should be acknowledged: Bacterial expression systems present challenges for studying some cardiomyopathy-associated proteins^34,35^, particularly those of a large size, such as titin^36^, or those requiring specialised cellular contexts for proper expression, such as skeletal muscle cells for myosin heavy chain beta (β-MHC)^37^. In addition, while *E. coli* provides a tractable platform for biophysical characterisation, it cannot recaptitulate key aspects of cardiomyocyte biology, including post-translational modifications, intracellular trafficking pathways and protein turnover. In particular, protein degradation pathways, such as the ubiquitin-proteosome system and autophagy-lysosomal pathway^38^, both implicated in HCM^39^, are absent. As such, these cellular consequences of variant-induced structural perturbations require validation in more physiologically relevant systems, including in vivo models^40^, neonatal rat cardiomyocytes transfection assays^41^, and human-induced pluripotent stem cell-derived cardiomyocytes (hiPSC-CMs)^42^.

More broadly, while our biophysical analyses revealed consistent structural vulnerabilities, these features cannot be directly equated with clinical pathogenicity, and discrepancies in variant classification illustrate the persistent gap between molecular characterisation and patient-level interpretation. In this context, the apparent structural stability of certain rod-domain and CaM variants may reflect benign effects; however, structural stability does not necessarily imply functional neutrality. Variants may still perturb interactions with binding partners, including myozenin-1, a known interactor of the ACTN2 rod-domain^43^, alongside additional ligands that have yet to be identified. Importantly, such effects may also arise through allosteric mechanisms, whereby variants within the rod-domain propagate conformational changes that influence distal functional sites, including actin-binding regions. This possibility is supported by evidence from homologous proteins, including Utrophin, where spectrin repeats contribute to actin-binding affinity^44^.

Further structural analyses will be important to contextualise these findings. In particular, higher-resolution approaches such as Cryo-electron microscopy^16^ of the full-length ACTN2/actin complex would enable visualisation of domain organisation and intermolecular interfaces that are not captured in truncated systems, while X-ray crystallography of ABD variants could provide atomic-level detail on local conformational changes. Finally, molecular dynamics simulations offer a complementary approach to capture the dynamic behaviour of ACTN2 variants over time. Such analyses could provide insight into conformational flexibility, interface stability, and thermal responses, helping to bridge structural and functional outcomes.

In summary, this study identifies multiple, variant-specific mechanisms through which ACTN2 missense substitutions contribute to HCM, including impaired actin-binding, reduced solubility, thermal instability, structural destabilisation, and aggregation. Our analysis further highlights that diverse mechanisms may arise even for variants within the same structural domain, indicating that a single unifying mechanism is unlikely; rather, each variant may drive HCM pathogenesis through multiple routes. Notably, the pronounced vulnerability of ABD variants underscores the importance of this domain in maintaining protein integrity and sarcomere organisation. By integrating diverse biophysical approaches into a systematic workflow, we provide a comprehensive framework for variant interpretation that can inform functional follow-up studies and potentially improve the classification of variants of uncertain significance linked to cardiomyopathy in genetic studies. Ultimately, these insights will advance the understanding of how structural perturbations in ACTN2 drive cardiomyopathic remodelling and lay the foundation for future therapeutic strategies.

## Methods

All experiments are discussed in further detail in the supplementary material. Raw data and uncropped gels for all main and supplementary figures are supplied in the source data file (see ‘Data availability’ statement).

### Mutagenesis and protein purification

ACTN2 missense variants were retrieved from the Human Gene Mutation Database (HGMD), and their pathogenicity was assessed using various in silico tools^4^, with 17 ACTN2 HCM-linked missense variants selected (see detailed methodology). The full-length ACTN2 cDNA cloned into the pET23a vector was transformed into competent DH5α *Escherichia coli* (*E.coli*) cells, and site-directed mutagenesis was performed to generate mutant proteins incorporating each of the 17 variants using designed primers (Table S6). Proteins were expressed using *E. coli* BL21-CodonPlus (DE3)-RP Competent Cells and purified using Nickel-NTA (NiNTA) agarose and size-exclusion chromatography.

### Solubility assay

Bacterial cell pellets were harvested for total lysate analysis. Protein was purified using NiNTA, and soluble and insoluble fractions were collected. Samples including total lysate, soluble, insoluble, and purified protein were denatured at 95 °C in the presence of 1x SDS sample buffer (composition in supplementary materials), and resolved using SDS-PAGE. Gels were stained with Coomassie Brilliant Blue to visualise protein bands, and densitometry was carried out using Image J.

### Differential scanning fluorimetry (DSF)

Thermal stability of the purified protein was assessed using differential scanning fluorimetry (DSF). Fluorescence emission at 330 nm and 350 nm was recorded by gradually increasing temperatures from 20 to 95 °C, and melting temperature (T_m_) was determined.

### Structural modelling

Structural models of ACTN2 incorporating nine selected variants were generated using the Phyre2 server^45^. The structural integrity of the wild-type (WT) and mutant forms of ACTN2 was analysed using PyMOL. The ACTN2-ABD/actin complex model was generated using HADDOCK^15^ (see detailed methodology in supplementary materials). Stabilising residues within the ACTN2 dimer were identified using the CCP4 programme, CONTACT^46^.

### High salt incubation using mass photometry

Mass photometry was performed using the Two MP instrument (Refeyn) to quantify the mass of ACTN2 samples. An equal amount of high salt solution (final concentration 2.5 M NaCl solution) was added to the WT and mutant (R327C and R457C) proteins, and measurements were recorded at 0 and 48 h with rigorous shaking. Standardisation was achieved by calibrating the instrument with bovine serum albumin (BSA) prior to data acquisition.

### X-ray crystallography

ACTN2-R327C and ACTN2-R457C variants were lysine methylated and crystallised in a reservoir containing 6.5% PEG smear high, 0.1 M HEPES buffer pH 6.5, 0.01 M EDTA and 0.17 M Magnesium Formate by the sitting-drop vapour diffusion method (1:1 protein/reservoir). Diffraction data were collected at the I03 beamline (Diamond Light Source, Harwell, UK). Structure determination and refinement is presented in the supplementary material. Data collection and refinement statistics are reported in Table S3.

### Actin-binding assays

Co-sedimentation assays were performed using 3 μM rabbit skeletal actin in conjunction with increasing concentrations of ACTN2 in binding buffer consisting of 150 mM KCl, 10 mM Tris-HCl, pH 7.5, and 4 mM MgCl_2_. Following ultracentrifugation using a Beckman TLA100 rotor, the concentration of ACTN2 bound to actin and the concentration of free ACTN2 in the solution were quantified, with calculations adjusted based on percentage pellet recovery. Densitometry was performed using Image Lab (Bio-Rad).

Reciprocal actin-binding assay was performed for WT, G111V and T247M using fixed ACTN2 concentration (3 μM) and increasing actin concentrations in binding buffer (300 mM KCl, 10 mM Tris-HCl, pH 7.5 and 4 mM MgCl_2_). Similar experimental conditions were used as above. Parameters binding affinity constant (K_d_) and maximum binding capacity (B_max_) were determined.

### Thermolysin digest in the presence and absence of actin

For proteolytic analysis, WT and variant ACTN2 samples (G111V, T247M) at 1 mg/mL were mixed with 10 μM rabbit skeletal actin (see section above) and 1 mg/mL of thermolysin at an enzyme-to-substrate molar ratio of 1:7:7 (Thermolysin : ACTN2 : Actin). In parallel, either actin or ACTN2 samples were mixed alone with thermolysin at 1:7 molar ratio. Other ACTN2 variants were tested using thermolysin only. All samples were incubated at 37 °C. Equal volumes of 5x SDS sample buffer were added to terminate reactions at specific time points. SDS-PAGE and Coomassie Brilliant Blue staining was used for visualisation.

### Size exclusion chromatography coupled with multi-angle X-ray scattering (SEC-MALS)

Size exclusion chromatography coupled with multi-angle light scattering (SEC-MALS) was employed using Superose 6 Increase 10/300 GL column (Cytiva) to determine the molar mass of WT and variant ACTN2 proteins using DAWN HELEOS II and Optilab T-rEX detectors (Wyatt Technology).

### Size exclusion chromatography coupled with small-angle X-ray scattering (SEC-SAXS)

Size exclusion chromatography coupled with small-angle X-ray scattering (SEC-SAXS) data was collected at B21 beamline (Diamond Light Source, Harwell, UK) using a KW-404 column (Shodex). Data analysis was performed using ScÅtter including log_10_ SAXS profiles and Guinier analysis. ScÅtter software was also used to determine radius of gyration with Guinier analysis *[R*_*g*_*(Guinier)]*, real space radius of gyration *[R*_*g*_*(real)]*, maximum particle dimension *(D*_*max*_*)*, particle volume *(V)*, and molecular weight (MW). Scattering electron density profiles were created using DENsity from Solution Scattering (DENSS) software^23^. Additional methodological details are reported in Table S7.

### Small-angle X-ray scattering (SAXS) using batch-mode

Protein samples were purified using a Superose 6 Increase 10/300 GL column, with fractions corresponding to dimer peak collected. SAXS data were then collected at B21 beamline. Measurements of SAXS were performed at six temperatures ranging from 40 to 60 °C. Data processing and analysis were performed using ScÅtter including log_10_ SAXS profiles, Guinier analysis, Kratky analysis, and Pair-distance *P(r)* distribution function, with the following parameters calculated: R_g_*(Guinier)*, R_g_*(real)*, *D*_*max*_, particle volume (*V*), and molecular weight (MW). DENSS was used to generate SAXS electron density models. Fast X-ray Scattering (FoXS) software was used to fit SAXS data to experimental data^47^. Additional methodological details are supplied in Table S7.

### Statistical analysis

Statistical analysis was performed using the GraphPad Prism software (v. 10.5.0; GraphPad Inc., San Diego, CA, USA). Solubility yield data were expressed as mean ± standard deviation (SD). Data were tested for normal distribution using the Shapiro-Wilk test. Data were normally distributed and one-way ANOVA was used for analysis, with Dunnett test used for multiple comparisons. Statistical significance was set at *p* ≤ 0.05, with significance levels indicated as follows: *p < 0.05, **p < 0.01, ***p < 0.001, ****p < 0.0001. A non-linear regression fit analysis (Saturation binding) was used to calculate actin-binding kinetics.

### Reporting summary

Further information on research design is available in the Nature Portfolio Reporting Summary linked to this article.

## Supplementary information

Supplementary Information

Reporting Summary

Transparent Peer Review file

## Data Availability

The experimental data on which this manuscript is based are made freely available through the Open Science Framework (OSF) public data repository (with no access restrictions), with source data files including data underlying plots and uncropped SDS-PAGE gels under [10.17605/OSF.IO/9TXMK]^48^. SAXS data are deposited in the Small Angle Scattering Biological Data Bank (SASBDB) under the following link [https://www.sasbdb.org/] and [reference IDs]: [SEC-SAXS Experiments: ACTN2-WT (SASDZJ4/K4), M92V (SASDZR5/Q5), R93Q (SASDZP5/N5), G111V (SASDZM5/L5), T247M (SASDZK5/J5), R327C (SASDZH5/G5), E448A (SASDZF5/D5), and R457C (SASDZE5/C5), with numbers corresponding to Peak 1/Peak 2]; [Batch-mode SAXS Experiments: ACTN2-WT (SASDZB5/A5/95), M92V (SASDZ85/75/65), R93Q (SASDZ55/45/35), G111V (SASDZ25/Z4/Y4), T247M (SASDZX4/W4/V4), R327C (SASDZU4/T4/S4), E448A (SASDZR4/Q4/P4), and R457C (SASDZN4/M4/L4), with numbers corresponding to dimer peak at 40/53/60 °C]. The X-ray crystallography structures have been deposited in RCSB Protein Data Bank under accession codes: 9SIR (Human Muscle Alpha-Actinin-2 Mutant R327C); 9SIS (Human Muscle Alpha-Actinin-2 Mutant R457C). The previously published PDB files referred to in this study include: 4D1E, 6M5G and 5A38.

## References

[1] Houston, B. A. & Stevens, G. R. Hypertrophic cardiomyopathy: a review. *Clin. Med. Insights Cardiol.* **8**, 53–65 (2014). 25657602 10.4137/CMC.S15717 PMC4309724

[2] Ommen, S. R. et al. Hypertrophic cardiomyopathy: state of the art. *Mayo Clin. Proc.* **100**, 557–566 (2025). 40044364 10.1016/j.mayocp.2024.07.013

[3] Noureddine, M. & Gehmlich, K. Structural and signaling proteins in the Z-disk and their role in cardiomyopathies. *Front. Physiol.* **14**, 1143858 (2023). 36935760 10.3389/fphys.2023.1143858 PMC10017460

[4] Noureddine, M. et al. Structural and functional insights into α-actinin isoforms and their implications in cardiovascular disease. *J. Gen. Physiol.* **157**, e202413684 (2025). 10.1085/jgp.202413684 PMC11804879 39918740

[5] Franzot, G. et al. The crystal structure of the actin binding domain from alpha-actinin in its closed conformation: structural insight into phospholipid regulation of alpha-actinin. *J. Mol. Biol.* **348**, 151–165 (2005). 15808860 10.1016/j.jmb.2005.01.002

[6] Speicher, D. W., Weglarz, L. & DeSilva, T. M. Properties of human red cell spectrin heterodimer (side-to-side) assembly and identification of an essential nucleation site. *J. Biol. Chem.* **267**, 14775–14782 (1992). 1634521

[7] Prondzynski, M. et al. Disease modeling of a mutation in α-actinin 2 guides clinical therapy in hypertrophic cardiomyopathy. *EMBO Mol. Med.* **11**, e11115 (2019). 31680489 10.15252/emmm.201911115 PMC6895603

[8] Theis, J. L. et al. Echocardiographic-determined septal morphology in Z-disc hypertrophic cardiomyopathy. *Biochem. Biophys. Res. Commun.* **351**, 896–902 (2006). 17097056 10.1016/j.bbrc.2006.10.119

[9] Chiu, C. et al. Mutations in alpha-actinin-2 cause hypertrophic cardiomyopathy: a genome-wide analysis. *J. Am. Coll. Cardiol.* **55**, 1127–1135 (2010). 20022194 10.1016/j.jacc.2009.11.016

[10] Haywood, N. J. et al. Hypertrophic cardiomyopathy mutations in the calponin-homology domain of ACTN2 affect actin binding and cardiomyocyte Z-disc incorporation. *Biochem. J.* **473**, 2485–2493 (2016). 27287556 10.1042/BCJ20160421 PMC4980809

[11] Atang, A. E. et al. Cardiomyopathy-associated variants alter the structure and function of the α-actinin-2 actin-binding domain. *Biochem. Biophys. Res. Commun.* **670**, 12–18 (2023). 37271035 10.1016/j.bbrc.2023.05.050 PMC13217505

[12] Stenson, P. D. et al. The Human Gene Mutation Database (HGMD(®)): optimizing its use in a clinical diagnostic or research setting. *Hum. Genet.* **139**, 1197–1207 (2020). 32596782 10.1007/s00439-020-02199-3 PMC7497289

[13] Landrum, M. J. et al. ClinVar: improving access to variant interpretations and supporting evidence. *Nucleic Acids Res.* **46**, D1062–d1067 (2018). 29165669 10.1093/nar/gkx1153 PMC5753237

[14] Karshikoff, A., Nilsson, L. & Ladenstein, R. Rigidity versus flexibility: the dilemma of understanding protein thermal stability. *FEBS J.* **282**, 3899–3917 (2015). 26074325 10.1111/febs.13343

[15] Dominguez, C., Boelens, R. & Bonvin, A. M. HADDOCK: a protein-protein docking approach based on biochemical or biophysical information. *J. Am. Chem. Soc.* **125**, 1731–1737 (2003). 12580598 10.1021/ja026939x

[16] Kumari, A. et al. Structural insights into actin filament recognition by commonly used cellular actin markers. *EMBO J.* **39**, e104006 (2020). 32567727 10.15252/embj.2019104006 PMC7360965

[17] Iwamoto, D. V. et al. Structural basis of the filamin A actin-binding domain interaction with F-actin. *Nat. Struct. Mol. Biol.* **25**, 918–927 (2018). 30224736 10.1038/s41594-018-0128-3 PMC6173970

[18] Avery, A. W. et al. Structural basis for high-affinity actin binding revealed by a β-III-spectrin SCA5 missense mutation. *Nat. Commun.* **8**, 1350 (2017). 29116080 10.1038/s41467-017-01367-w PMC5676748

[19] Fealey, M. E. et al. Dynamics of dystrophin’s actin-binding domain. *Biophys. J.* **115**, 445–454 (2018). 30007583 10.1016/j.bpj.2018.05.039 PMC6084637

[20] Weins, A. et al. Disease-associated mutant alpha-actinin-4 reveals a mechanism for regulating its F-actin-binding affinity. *Proc. Natl. Acad. Sci. USA*. **104**, 16080–16085 (2007). 17901210 10.1073/pnas.0702451104 PMC2042165

[21] Borrego-Diaz, E. et al. Crystal structure of the actin-binding domain of alpha-actinin 1: evaluating two competing actin-binding models. *J. Struct. Biol.* **155**, 230–238 (2006). 16698282 10.1016/j.jsb.2006.01.013

[22] Yue, P., Li, Z. & Moult, J. Loss of protein structure stability as a major causative factor in monogenic disease. *J. Mol. Biol.* **353**, 459–473 (2005). 16169011 10.1016/j.jmb.2005.08.020

[23] Grant, T. D. Ab initio electron density determination directly from solution scattering data. *Nat. Methods* **15**, 191–193 (2018). 29377013 10.1038/nmeth.4581

[24] Ribeiro Ede, A. Jr. et al. The structure and regulation of human muscle α-actinin. *Cell* **159**, 1447–1460 (2014). 25433700 10.1016/j.cell.2014.10.056 PMC4259493

[25] Putnam, C. D. et al. X-ray solution scattering (SAXS) combined with crystallography and computation: defining accurate macromolecular structures, conformations and assemblies in solution. *Q. Rev. Biophys.* **40**, 191–285 (2007). 18078545 10.1017/S0033583507004635

[26] Couthon, F., Clottes, E. & Vial, C. High salt concentrations induce dissociation of dimeric rabbit muscle creatine kinase. Physico-chemical characterization of the monomeric species. *Biochim. Biophys. Acta* **1339**, 277–288 (1997). 9187248 10.1016/s0167-4838(97)00010-1

[27] Mrabet, N. T. et al. Dissociation of dimers of human hemoglobins A and F into monomers. *J. Biol. Chem.* **261**, 1111–1115 (1986). 2418013

[28] Djinović-Carugo, K. et al. Molecular basis for cross-linking of actin filaments: structure of the α-actinin rod. *Cell* **98**, 537–546 (1999). 10481917 10.1016/s0092-8674(00)81981-9

[29] Atang, A. E., Keller, A. R., Denha, S. A. & Avery, A. W. Increased actin binding is a shared molecular consequence of numerous SCA5 mutations in β-III-spectrin. *Cells* **12**, 2100 (2023). 10.3390/cells12162100 PMC10453832 37626910

[30] Morihara, K. & Tsuzuki, H. Thermolysin: kinetic study with oligopeptides. *Eur. J. Biochem.* **15**, 374–380 (1970). 4993757 10.1111/j.1432-1033.1970.tb01018.x

[31] Brunet, T. et al. De novo variants in neurodevelopmental disorders—experiences from a tertiary care center. *Clin. Genet.* **100**, 14–28 (2021). 33619735 10.1111/cge.13946

[32] Salinas, V. et al. The odyssey of complex neurogenetic disorders: From undetermined to positive. *Am. J. Med. Genet. C Semin. Med. Genet.* **184**, 876–884 (2020). 33084218 10.1002/ajmg.c.31848

[33] Reis, M. C. et al. Kv3.3 expression enhanced by a novel variant in the Kozak sequence of KCNC3. *Int. J. Mol. Sci.* **25**, 12444 (2024). 10.3390/ijms252212444 PMC11595341 39596509

[34] Domínguez, F. et al. Titin missense variants as a cause of familial dilated cardiomyopathy. *Circulation* **147**, 1711–1713 (2023). 37253077 10.1161/CIRCULATIONAHA.122.062833

[35] Woo, A. et al. Mutations of the beta myosin heavy chain gene in hypertrophic cardiomyopathy: critical functional sites determine prognosis. *Heart* **89**, 1179–1185 (2003). 12975413 10.1136/heart.89.10.1179 PMC1767874

[36] Jin, J. P. Titin-thin filament interaction and potential role in muscle function. *Adv. Exp. Med. Biol.* **481**, 319–333 (2000). discussion 334-5. 10987081 10.1007/978-1-4615-4267-4_19

[37] McMillan, S. N., Pitts, J. R. T., Barua, B., Winkelmann, D. A. & Scarff, C. A. Mavacamten inhibits myosin activity by stabilising the myosin interacting-heads motif and stalling motor force generation. *Sci. Adv.* **12**, eaea9335 (2026). 10.1126/sciadv.aea9335 PMC13127578 42054462

[38] Li, Y., Li, S. & Wu, H. Ubiquitination-Proteasome System (UPS) and autophagy two main protein degradation machineries in response to cell stress. *Cells* **11**, 851 (2022). 10.3390/cells11050851 PMC8909305 35269473

[39] Zech, A. T. L. et al. ACTN2 mutant causes proteopathy in human iPSC-derived cardiomyocytes. *Cells* **11**, 2745 (2022). 10.3390/cells11172745 PMC9454684 36078153

[40] Noureddine, M. et al. Atrial electrical alterations with intact cardiac structure and contractile function in a mouse model of an HCM-linked ACTN2 variant. *J. Mol. Cell. Cardiol.* **12**, 100455 (2025). 10.1016/j.jmccpl.2025.100455 PMC12153375 40503000

[41] Pearce, A. et al. Missense mutations in the central domains of cardiac myosin binding protein-C and their potential contribution to hypertrophic cardiomyopathy. *J. Biol. Chem.* **300**, 105511 (2024). 38042491 10.1016/j.jbc.2023.105511 PMC10772716

[42] Cumberland, M. J. et al. Generation of a human iPSC-derived cardiomyocyte/fibroblast engineered heart tissue model. *F1000Research* **12**, 1224 (2023). 38298530 10.12688/f1000research.139482.2 PMC10828555

[43] Sponga, A. et al. Order from disorder in the sarcomere: FATZ forms a fuzzy but tight complex and phase-separated condensates with α-actinin. *Sci. Adv.* **7**, eabg7653 (2021). 10.1126/sciadv.abg7653 PMC8163081 34049882

[44] Rybakova, I. N. & Ervasti, J. M. Identification of spectrin-like repeats required for high affinity utrophin-actin interaction. *J. Biol. Chem.* **280**, 23018–23023 (2005). 15826935 10.1074/jbc.M502530200

[45] Kelley, L. A. et al. The Phyre2 web portal for protein modeling, prediction and analysis. *Nat. Protoc.* **10**, 845–858 (2015). 25950237 10.1038/nprot.2015.053 PMC5298202

[46] Agirre, J. et al. The CCP4 suite: integrative software for macromolecular crystallography. *Acta Crystallogr. D Struct. Biol.* **79**, 449–461 (2023). 37259835 10.1107/S2059798323003595 PMC10233625

[47] Schneidman-Duhovny, D., Hammel, M. & Sali, A. FoXS: a web server for rapid computation and fitting of SAXS profiles. *Nucleic Acids Res.* **38**, W540–W544 (2010). 20507903 10.1093/nar/gkq461 PMC2896111

[48] Gehmlich, K., Noureddine, M. & Mohammed, F. Data related to “Comprehensive biophysical and structural profiling of alpha-actinin-2 variants reveals mechanistic diversity in hypertrophic cardiomyopathy”. OSF project. 10.17605/OSF.IO/9TXMK (2026). 10.1038/s41467-026-75392-z PMC13388997 42481460

