# Supplementary material for: Comprehensive biophysical and structural profiling of alpha-actinin-2 variants reveals mechanistic diversity in hypertrophic cardiomyopathy
Source: Nat Commun. 2026 Jul 21;17:6127. doi: 10.1038/s41467-026-75392-z (PMC13388997; doi:10.1038/s41467-026-75392-z)
Supplement: Supplementary file 1 — Supplementary Information [file 41467_2026_75392_MOESM1_ESM.pdf]

Comprehensive Biophysical and Structural Profiling of Alpha-actinin-2 Variants Reveals Mechanistic Diversity in Hypertrophic Cardiomyopathy

Supplementary Information

Maya Noureddine[1], Halina Mikolajek[2], Nathan Cowieson[2], Nikos Pinotsis[3], Paul Robinson[4], Alexandre Slater[1], Charles Redwood[4], Siobhan Loughna[5], Chris Denning[6], Fiyaz Mohammed[7]*, Katja Gehmlich[1, 4]*

* F. Mohammed and K. Gehmlich contributed equally to this work

Corresponding:

Maya Noureddine: m.noureddine@bham.ac.uk

Fiyaz Mohammed: f.mohammed@bham.ac.uk

Katja Gehmlich: k.gehmlich@bham.ac.uk

[1]Department of Cardiovascular Sciences, University of Birmingham, Birmingham, United Kingdom

[2]Diamond Light Source Ltd, Didcot, United Kingdom

[3]Birkbeck College, London, United Kingdom

[4]Division of Cardiovascular Medicine, Radcliffe Department of Medicine and British Heart Foundation Centre of Research Excellence Oxford, University of Oxford, Oxford, United Kingdom

[5]School of Life Sciences, University of Nottingham, Nottingham, United Kingdom

[6]Biodiscovery Institute, University of Nottingham, Nottingham, United Kingdom

[7]Department of Immunology and Immunotherapy, University of Birmingham, Birmingham, United Kingdom

Contents:

Detailed Methodology

Tables S1-S7

Supplementary Figures S1-S22

Detailed Methodology

1. Methods

1.1. ACTN2 Variants Identification

The Human Gene Mutation Database (HGMD) [1] served as a critical resource in identifying and retrieving ACTN2 missense variants. The pathogenicity of these variants was assessed using minor allelic frequency (MAF) values obtained from the Genome Aggregation Database (GnomAD) with a cutoff of $1x\ 10^{-4}$ [2] and *in silico* tools, such as SIFT (Sorting Intolerant from Tolerant) [3], and PolyPhen-2 (Polymorphism Phenotyping v2) [4]. Seventeen ACTN2 variants linked to HCM were selected[5]. These variants were further assessed by retrieving interpretations derived from ClinVar database[6]. Relevant reports from HGMD were reviewed to retrieve associated clinical data, with summary presented in Table S1.

1.2. Mutagenesis of ACTN2 Plasmid

The modified pET23a plasmid (Novagen) containing full-length human ACTN2 cDNA, with N-terminal T7 immunotag and C-terminal Hexahistidine-tag [7] was transformed into DH5α *Escherichia. coli (E. coli)* competent cells (ThermoFisher Scientific) via heat shock at 42°C to facilitate plasmid uptake. A single colony was selected, propagated in LB-broth supplemented with 100 µg/mL ampicillin, and used for plasmid DNA isolation employing the Qiagen Mini-prep method (QIAprep Spin Miniprep Kit). The ACTN2 wild-type (WT) plasmid sequence was confirmed using Sanger DNA sequencing (Source BioScience). The purified mini-prep of the ACTN2 wild-type (WT) plasmid DNA served as a template for generating ACTN2 missense variants using the QuikChange II XL Site-Directed Mutagenesis Kit (Agilent). Variant-specific primers were designed following the manufacturer's guidelines and prepared at 10µM (Agilent QuikChange Primer Design) (Table S6). Mutagenesis PCR reaction was conducted in a 25 µL reaction mixture: 1 µL ACTN2-WT plasmid DNA mini-prep, 2.5 µL 10x reaction buffer, 1.5 µL QuikSolution, 1 µL each of the forward and reverse primers, 0.5 µL of dNTP mix, and 0.5 µL pFu Ultra High Fidelity DNA polymerase (2.5 U/µl). PCR reaction was carried out using a MiniAmp Plus thermal cycler (Applied Biosystems). The thermal cycling conditions were as follows: denaturation at 95°C for 1 minute; 18 cycles of 95°C for 50 sec, 60°C for 50 sec, and 68°C for 6.5 minutes (1min/kb). A final extension step was applied at 68°C for 7 minutes. PCR products were digested with 0.5 µL DpnI (10 U/µl) at 37°C for 1 hour. Digested PCR products were transformed into XL10-Gold Ultracompetent Cells (Agilent), and single colonies were selected, propagated and plasmid DNA was purified using the Qiagen Mini-prep method. Successful incorporation of the desired single missense mutation, without additional sequence alterations, was confirmed using Sanger DNA sequencing (Source BioScience).

1.3. Small-scale Protein Expression and Purification

Plasmid DNA encoding either WT or ACTN2 variant was transformed into *E. coli* BL21-CodonPlus(DE3)-RP Competent Cells (Agilent). Single colonies were inoculated into 4 mL of LB broth supplemented with 100 µg/mL of ampicillin and 50 µg/mL of chloramphenicol and grown overnight at 37°C with shaking at 200-225 rpm. The following day, cultures were expanded to 5ml in fresh antibiotic-containing LB broth. After 1 hour incubation ($OD_{600} \approx 0.6$), protein expression was induced with 1mM isopropyl β-D-1-thiogalactopyranoside (IPTG). Cultures were incubated for an additional 3 hours, after which bacterial pellets were harvested by centrifugation at 900 g for 5 minutes at 4°C.

1.4. Solubility Assays

Bacterial cell pellets were harvested for total lysate analysis and resuspended in 100µl of 1x SDS sample buffer (consisting of 6% (v/v) glycerol, 2% (w/v) sodium dodecyl sulfate, 100 mM dithiothreitol, and 62.5 mM Tris-HCL pH 6.8 with addition of bromophenol blue in trace amounts), followed by sonication (Vibra cell, SONICS) for 5-10 seconds. For solubility assessment, separate bacterial pellets were resuspended in 500 µl of cold phosphate-buffered saline (PBS) and sonicated for five 10s cycles. Lysates were centrifuged at 17,000 g for 10 minutes at 4°C, and 20 µl of the resulting supernatant (soluble fraction) was collected and mixed with 20 µl 5x SDS sample buffer. An additional aliquot of the supernatant fraction was incubated with Ni-NTA beads (HIS-Select Nickel Affinity Gel, Merck) for 1 hour at 4°C with gentle rotation. Beads were washed three times with PBS containing 20 mM imidazole. Bound protein was eluted by adding 30 µl 5x SDS sample and heating at 95°C for 3 minutes. The insoluble fraction was recovered by resuspending the post-centrifugation pellet in 500 µl of cold PBS. A 50µl was taken, mixed with an equal volume of 5x SDS sample buffer, and sonicated for 5-10 seconds. All samples including total lysate, soluble and insoluble fractions and purified proteins were heated at 95°C for 3 minutes and resolved by SDS-PAGE (Bio-Rad 4-15 % TGX, cat no. 4561084). Gels were stained with Coomassie Brilliant Blue to visualise protein bands. Densitometry was quantified using Image J software (v.1.54), and protein yield was calculated as the ratio of purified protein to total lysate, and normalised against the WT sample.

1.5. Large-scale Protein Expression and Purification

Recombinant WT and mutant ACTN2 constructs were transformed into *E. coli* BL21-CodonPlus (DE3)-RP Competent Cells and cultured in 1 L of SOC media (ThermoFisher Scientific) supplemented with 20 mM glucose, 5 mM $MgCl_2$, 5 mM $MgSO_4$, 100 µg/mL of ampicillin and 50 µg/mL of chloramphenicol. Cultures were grown at 37°C with shaking at 180 rpm until reaching an OD_{600} of ~0.6, at which point protein expression was induced with 0.5 mM IPTG. Cultures were incubated for an additional 5-6 hours at 28°C, then harvested by centrifugation at 1900 g for 15 minutes at 4°C. Cell pellets were resuspended in PBS containing 10 mM imidazole and lysed by sonication (10 micron amplitude; 10 cycles of 30 seconds on/30 seconds off, on ice). Lysates were clarified by centrifugation at 15,000 g, for 30 minutes at 4°C, and the supernatant was filtered through a 0.22 µm syringe filter. The supernatant was incubated overnight at 4°C with 1 mL of Ni-NTA agarose beads (pre-equilibrated with PBS and 10 mM imidazole) under gentle rotation. The following day, beads were washed 3 times with PBS containing 20 mM imidazole, and bound proteins were eluted using PBS supplemented with 500 mM imidazole via gravity flow through 2 mL disposable columns (PierceTM Disposable Columns, ThermoFisher Scientific). Eluted fractions were analysed by SDS-PAGE and visualised by Coomassie Brilliant Blue staining. Ni-NTA purified proteins were concentrated using Amicon Ultra 30kDa centrifugal units (Merck) and filtered through a 0.22 µm syringe filter.

For further purification, size exclusion chromatography (SEC) was performed using an ÄKTA pure chromatography system equipped with a Superose 6 Increase 10/300 GL column (Cytiva) at 4°C. The column was equilibrated with 20 mM HEPES pH 8 and 150 mM NaCl, with a flow rate of 0.5 mL/min. A volume of 500 µl of Ni-NTA purified proteins was loaded onto the column. Elution was monitored at 280 nm, and elution profiles were analyzed using UNICORN software (v.7.3). Fractions eluting as the second peak were collected for further experiments and concentrated using Amicon Ultra 30kDa centrifugal units (Merck). Final protein

concentrations were measured by UV absorbance at 280 nm. Due to precipitation upon snap-freezing, purified proteins were stored at 0°C, with media cooling replaced every few days to maintain stability.

1.6. Differential Scanning Fluorimetry (DSF)

Thermal stability of the purified protein was assessed using a Prometheus NT.48 nano-Differential Scanning Fluorimetry (nano-DSF) instrument (NanoTemper Technologies). Protein samples were prepared at 1mg/ml, and 10 µL of each sample was loaded into standard-grade glass capillaries (Prometheus Standard Capillaries, cat no. SKU:PR-C002). Thermal unfolding was monitored by gradually increasing temperatures from 20 to 95°C at a rate of 1.5°C/min, with an excitation power set at 40 %. Fluorescence emission at 330 nm and 350 nm was recorded during the temperature ramp, and the ratio of fluorescence intensity at 350nm to 330 nm was calculated. The melting temperature (T_m) was determined from the fluorescence ratio curve, with T_{m0} representing the onset of unfolding, and T_{m1} corresponding to the inflection point. Data acquisition and analysis was performed using P.R.ThermControl software (v. 2.3.1).

1.7. Structural Modelling

1.7.1. Model Generation of ACTN2 Variants using Phyre2

The ACTN2 sequence was retrieved from UniProt (accession code P35609) and modified to incorporate HCM-linked variants through specific amino acid substitutions. Sequences corresponding to the ACTN2 domains were submitted to Phyre2 protein homology/analogy recognition server [8] to generate structural models of the corresponding domain structure incorporating the variants. The highest-confidence Phyre2 models, based on template alignment coverage and confidence scores, were selected for downstream structural analysis. Comparative analysis was performed between wild-type ACTN2 crystal structure (PDB ID: 4D1E) [9] and variant-containing models to evaluate alterations in residue interactions and local structural stability. Differences in molecular contacts between variant residues and surrounding amino acids were visualised and analysed using PyMOL (Schrödinger) [10].

1.7.2. Generation of the ACTN2-CH1/actin Complex Model using HADDOCK

A structural model of the ACTN2-CH1/actin complex was generated using High Ambiguity Driven protein-protein DOCKing (HADDOCK) [11], an information-driven docking software that incorporates restraints to guide model building. To define interaction restraints for docking, closely related homologues of ACTN2-ABD in complex with actin were assessed. The highest resolution structure among these was the cryo-electron microscopy structure of Utrophin-ABD in complex with actin (PDB ID: 6M5G) [12]. The structure identified three discrete actin-binding sites that contain residues conserved in ACTN2. Based on sequence alignment with ACTN2, the corresponding regions were defined as ABD1 (A35-R41), ABD2' (K91-N101), and ABD2 (V115-M133). These conserved segments were used as ambiguous interaction restraints to guide the docking of the ACTN2-CH1 domain ontoactin. The resulting docking models were ranked based on standard HADDOCK scoring metrics. Selection criteria included the overall HADDOCK score , cluster Z-score and interface energetics including electrostatic energy, Van der Waals energy, and buried surface area.Models exhibiting the most favourable combination of these parameters were considered highest confidence, and the top-ranked cluster representative was selected as the most probable structural model of the ACTN2-

CH1/actin complex. Docking simulations for ACTN2 variant proteins, including M92V and R93Q, in complex with actin were performed using identical restraint definitions to ensure comparability between models.

1.7.3. Analysis of Dimer Interface Residues using CONTACT

Residues involved in stabilising the ACTN2 dimer interface within the full-length ACTN2 crystal structure (PDB ID: 4D1E) [9] were identified using the CONTACT program within the CCP4 crystallographic software suite [13]. Polar interactions were defined by interatomic distances of 2.3-3.5 Å between relevant oxygen and nitrogen atoms, while non-polar interactions were defined by distances of 3-4.5 Å between relevant carbon-carbon and carbon-sulphur atoms.

1.8. High-salt Incubation Assessment using Mass Photometry

Wild-type and variant ACTN2 protein samples (R327C and R457C) were purified using SEC and fractions eluting as second peak were used. Samples were incubated with an equal amount of high salt solution (2.5 M of NaCl solution). Mass photometry measurements were taken initially at 0 hours using a Two MP instrument (Refeyn). Aliquots of ACTN2 samples were diluted to 0.1 µM in PBS and applied to a clean microscope coverslip fitted with CultureWell gaskets (Grace Bio-labs, cat no. CW-50R-1.0) to take measurments. Data acquisition was carried out using Acquire MP software (Refeyn, v. 2.5.0) and analysed using Discover MP software (Refeyn, v. 2024). Bovine serum albumin (BSA) also diluted to 0.1 µM in PBS was used as a standard for molecular mass calibration. The samples were then subjected to continuous agitation for 48 hours at 4°C. Mass photometry measurements were repeated after this incubation period.

1.9. X-ray Crystallography

1.9.1. Protein Crystallisation

Protein samples (ACTN2-R327C and ACTN2-R457C) were lysine methylated using reagents from the crystal growth kit (Hampton research, cat no. HR2-434), following manufacturer's protocol [14]. To remove methylation traces, samples underwent an additional purification step via SEC with column pre-equilibrated with 20 mM HEPES pH 8 and 150 mM NaCl. Pure methylated protein was used at a final concentration of 7 mg/ml for the R327C variant and 5 mg/ml for R457C variant. Crystallisation experiments were performed at the Crystallisation Facility within the Research Complex at Harwell (UK) using a SPT Labtech Mosquito robot. Proteins were crystallised using the sitting-drop vapour diffusion method in 96-well InSitu-1 crystallisation plates (MiTeGen, USA, cat no. InSitu-01CL-40). Each drop comprised of 200 nL of protein-reservoir mixture (1:1 protein/reservoir) and was equilibrated against a reservoir containing 6.5% PEG smear high (Molecular Dimensions), 0.1 M HEPES buffer pH 6.5, 0.01 M Ethylenediaminetetraacetic Acid (EDTA) and 0.17 M Magnesium Formate. Crystallisation plates were initially incubated at 16°C for 3 days before being transferred to Formulatrix imaging systems at 20°C for an additional 3-5 days. Preliminary diffraction data were collected from selected crystals at the VMXi beamline (Diamond Light Source, Harwell, UK). Remaining crystals were cryoprotected in reservoir solution supplemented with 15% PEG smear high.

1.9.2. Structure Determination and Refinement

Crystals of the ACTN2 variants R327C and R457C were measured at the I03 beamline (Diamond Light Source, Harwell, UK) using the unattended data collection mode. Several crystals for both ACTN2 variants were collected and the data set with the best diffraction statistics was selected for structure determination. All ACTN2 crystals belonged to the $P\,2\,2_1\,2_1$ space group, with a solvent content of 65.2 %, corresponding to one molecule (or half of the ACTN2 dimer) per asymmetric unit. Structures were solved by molecular replacement using MOLREP [15], with the native structure as the search model (PDB ID: 4D1E) [9]. After an initial rigid body refinement using PHENIX refine [16], the model was inspected in COOT [17] where the individual R327C and R457C were manually introduced into the respective models. Further model refinement was carried out iteratively thorough maximum-likelihood and TLS refinement using the PHENIX suite, coupled with manual adjustments in COOT to optimise model geometry, improve Ramachandran statistics, and remove poorly defined side chains lacking electron density. The final refined structures of both ACTN2 mutants encompass the human ACTN2 amino-acid sequence 34-892. ACTN2-R327C and ACTN2-R457C refined structures were aligned with ACTN2-WT (PDB ID: 4D1E), using PDBeFold (EMBL-EBI) and root mean square deviation (RMSD) values were calculated [18].

1.10. Actin-binding Assays

Protein samples were purified using the ÄKTA pure chromatography system equipped with a Superose 6 Increase 10/300 GL column, with fractions eluting as second peak being used. Co-sedimentation binding assays were used to determine binding of ACTN2 to actin. ACTN2 samples were subjected to an initial spin at 240,000 g for 30 minutes at 4°C using a 5 mL Open-Top Thinwall Ultra-Clear Tube (Beckman Coulter, cat no: 344057) in an Optima MAX-XP Ultracentrifuge and a Beckman Coulter MLS-50 swinging-bucket rotor, and the resulting ACTN2 supernatant was collected. Native rabbit skeletal actin was isolated after preparation of acetone powder [19]. Fixed concentration of actin (3 μM) was mixed with increasing concentrations of ACTN2 (0-12 μM for WT, G111V, T247M, and 0-10 μM for M92V, R93Q and S147L). Actin-binding buffer consisted of 150 mM KCl, 10 mM Tris-HCl pH 7.5, and 4 mM $MgCl_2$, and samples were mixed in a total sample volume of 100 μL. For total lysate protein, 30 μL from each sample were mixed with 10 μL of 4x SDS sample buffer. The remaining 70 μL were transferred to 0.2 mL Open-Top Thickwall Polycarbonate Tube (Beckman Coulter, cat no: 343775) and centrifuged at 436,000 g for 15 minutes at 4°C using a Beckman TLA-100 fixed-angle rotor. 30 μL of supernatant from each sample was collected and mixed with 10 μL of 4x SDS sample buffer. Pellets were resuspended in 92.8 μL of 1x SDS sample buffer. Control samples containing the similar ACTN2 concentrations but without actin were processed in parallel to determine actin-independent ACTN2 sedimentation. Pellet and total lysate fractions were analysed by 4-15% SDS-PAGE (Bio-Rad TGX, cat no: 4561084), followed by Coomassie Brilliant Blue staining. Representative gels showing total lysate, supernatant and pellet were analysed using 4-15% SDS-PAGE (Bio-Rad Criterion TGX gels, cat no. 5671085).

A reciprocal actin-binding experiment was also performed for WT and ACTN2 variants (G111V and T247M). A fixed ACTN2 concentration of 3 μM was used and mixed with increasing concentrations of actin (0-30 μM) in a total sample volume of 100 μL. To maintain ionic strength, a buffer with a higher concentration of KCL was used (300 mM KCl, 10 mM Tris-HCl pH 7.5, and 4 mM $MgCl_2$). The reaction was performed under conditions similar to

those described above. Control samples containing the similar actin concentrations but without ACTN2 were also processed in parallel.

Band intensities were quantified using densitometry (Image Lab v. 6.1, Bio-Rad). The background intensity of each lane was subtracted from the corresponding protein band intensity to correct for any background signal. Concentration of ACTN2 bound to actin versus free ACTN2 in solution were adjusted by percentage pellet recovery. In particular, densitometry of ACTN2 bound was adjusted by the ACTN2 recovered and non-actin dependent sedimentation of the ligand. Free ACTN2 is calculated by subtracting the calculated bound from the total protein inputted through the assay. Binding affinity constant (K_d) and the maximum binding capacity (B_{max}) were determined by nonlinear regression analysis using a saturation binding model in GraphPad Prism (v.10.5.0) using the following equation of [$Y=(B_{max}.X)/(K_d+X)$] [20], where $Y=[ACTN2]_{bound}$ and $X= [ACTN2]_{free}$.

1.11. Thermolysin Digest in the Presence and Absence of Actin

Wild-type and variant ACTN2 samples were purified via SEC and fractions eluting as second peak were collected and prepared at 1mg/mL. Isolated native rabbit skeletal actin (see section 1.10) was used at 10µM and mixed with ACTN2-WT, G111V, T247M (at 1 mg/mL) together with thermolysin (Target Mol; cat no. T76009) prepared at concentration of 1 mg/mL by maintaining an enzyme-to-substrate molar ratio of 1:7:7 (thermolysin:ACTN2:Actin). Samples were diluted using actin-binding buffer (150 mM KCl, 10 mM Tris-HCl pH 7.5, and 4 mM $MgCl_2$) in a total sample volume of 40 µL. Reactions were terminated at specific time points (at 5, 10, 30 minutes and 1, 2, 16 hours) by the addition of equal volumes of 5x SDS sample buffer. In parallel, control samples containing either actin with thermolysin or ACTN2 with thermolysin were processed under the same conditions. The samples were then heated at 95°C for 3 minutes. Proteolytic digestion products were analysed by SDS-PAGE, followed by staining with Coomassie Brilliant Blue to visualise the protein fragments.

Similar analysis was performed for the remaining ACTN2-ABD variants (M92V, R93Q, S147L) and rod variants (R327C, E448A, R457C, I653T). ACTN2 samples were mixed with thermolysin only, both at 1 mg/mL, while maintaining an enzyme-to-substrate molar ratio of 1:7 (thermolysin:ACTN2). Reactions were stopped and visualised as described above.

1.12. Size Exclusion Chromatography Coupled with Multi-angle Light Scattering (SEC-MALS)

Size exclusion chromatography coupled with multi-angle light scattering (SEC-MALS) was employed to determine parameters including, differential refractive index (dRI), light scattering (LS), UV absorbance (UV), and molar mass of WT and variant ACTN2 proteins. The analyses were carried out using an ÄKTA pure chromatography system equipped with a Superose 6 Increase 10/300 GL column (Cytivia) at room temperature. The column was equilibrated with 20 mM HEPES pH 8 and 150 mM NaCl at a flow rate of 0.5 mL/min. A volume of 100 µL of protein samples, prepared at concentrations of 2-2.5 mg/mL, were injected onto the column. The eluate was monitored using a DAWN HELEOS II multi-angle light scattering detector and an Optilab T-rEX differential refractive index detector (Wyatt Technology). Data acquisition and processing were performed using ASTRA software (v. 7.3.2), from which the molecular weight (MW), hydrodynamic radius (R_H), and polydispersity index (PDI) of the proteins were determined.

1.13. Size Exclusion Chromatography coupled with Small-angle X-ray Scattering (SEC-SAXS)

Size exclusion chromatography coupled with small-angle X-ray scattering (SEC-SAXS) data were collected at the B21 beamline (Diamond Light Source, Harwell, UK) using an in-line high-performance liquid chromatography (HPLC) system (Agilent) and an EigerX 4M (Dectris) detector [21]. The beamline was used in its default configuration with a beam energy of 13.1 keV, $4x10^{12}$ photons per second, and a sample-detector distance of 3.7 m. Protein samples were filtered through a 0.22 µm membrane and prepared at a concentration of 6-8 mg/mL. A volume of 50 µL was injected onto a KW-404 coloumn (Shodex) at a flow rate of 0.16 mL/min into a 1.5 mm diameter glass capillary in the X-ray beam, equilibrated with a buffer of 20 mM HEPES pH 8, 150 mM NaCl, and 1% glycerol to minimise the impact of radiation damage. The system was calibrated with bovine serum albumin (BSA) to validate the SEC-SAXS setup. The scattering intensity *(I)* in the range of momentum transfer $0.0045 < q < 0.34$ Å^{-1} was recorded ($q = 4\pi\sin(\theta)/\lambda$, where X-ray wavelength $\lambda = 0.94$ Å, scattering angle is 2θ, and q is the scattering vector). The collected SEC-SAXS data for each sample consisted of 600 frames. The scattering profiles of the eluted protein peaks 1 or 2 (P1-P2) were averaged after subtraction of selected background frames using CHROMIXS [22] and ScÅtter software (v. IV.d, BIOISIS). Azimuthal integration of the 2D images to 1D scattering curves was performed via DAWN [23]. The forward scattering $I(0)$ and radius of gyration $[R_g(Guinier)]$ was determined using the Guinier approximation with a Guinier region of ($q.R_g < 1.3$). Log_{10} SAXS profiles and pair distribution function of the particle $P(r)$ was performed using ScÅtter and real space radius of gyration $[R_g(real)]$ and maximum particle dimension (D_{max}) were determined. ScÅtter was also used to derive particle volume *(V),* with molecular weight (MW) estimated using the following formula, MW=V/1.66 [24, 25]. Electron density profiles of SEC-SAXS scattering data of peak 2 (P2) were generated using DENsity from Solution Scattering (DENSS) software [26], and overlined with ACTN2 X-ray crystal structure (PDB ID: 4D1E) [9] using Chimera (v. 1.19) and PyMOL (Schrödinger) [10].

1.14. Small-angle X-ray Scattering (SAXS) Thermal Analysis using Batch-mode

Protein samples were purified using the ÄKTA pure chromatography system equipped with a Superose 6 Increase 10/300 GL column. Fractions corresponding to the dimer peak (eluting as peak 2) were collected, concentrated to 1 mg/mL and 35 µl were loaded into 1.5 mm quartz capillary tubes (Quarzkapillaren, Hampton Research) using a liquid handling robot at a rate of 1 µl/s. SAXS data were collected at B21 beamline (Diamond Light Source, Harwell, UK). A temperature-controlled sample exposure unit (SEU) was used, and measurements were performed at six temperatures (°C): 40, 47, 53, 55, 57, and 60. At each temperature, 20x 1s exposures were made for each sample and replicate measurements were averaged following rejection of outliers (i.e radiation damage). Data processing and analysis were performed using ScÅtter software. Parameters including $R_g(Guinier)$, $R_g(real)$, D_{max}, V, *and* MW at each measured temperature were derived using ScÅtter. 1D SAXS profiles and Kratky plots were also generated using PRIMUS [27]. Scattering electron density profiles were created using DENSS [26]. SAXS data was fitted to the crystal structure (PDB ID: 4D1E) using Fast SAXS Profile Computation algorithm (FoXS) [28].

Domains	ACTN2 Variants	Nucleotide Change	ClinVar Interpretation	Cases/HCM/symptoms	Reference
ABD	M92V	c.58A > G	Uncertain significance	1 case/807	[29]
	R93Q	c.274A > G	Uncertain significance (5) and benign (1) #	1/ 242 (African)	[30]
	G111V	c.278G > A	Uncertain significance	1 case/ myocyte hypertrophy and disarray, endocardial fibrosis	[31]
	S147L	c.332G > T	Uncertain significance (1) and likely benign (1) #	1/89	[32]
	T247M	c.440C > T	Likely pathogenic	1 case/ LVH, LVOT, atypical AFL, and paroxysmal AFib	[33]
Rod	D325Y	c.740C > T	Absent	1/45	[34]
	R327C	c.973G > T	Uncertain significance	1/807	[29]
	R353W	c.979C > T	Uncertain significance (4) and likely benign (1) #	1/632	[29]
	R398H	c.1057C > T	Likely benign	1/758	[35]
	E448A	c.1193G > A	Absent	1/212	[36]
	E448K	c.1343A > C	Uncertain significance	1/34/asymmetric septal hypertrophy, NYHA I	[37]
	R457C	c.1342G > A	Uncertain significance (1) and likely benign (1) #	1/758	[35]
	R572W	c.1369C > T	Uncertain significance (2) and likely benign (1) #	1/807	[29]
	E628G	c.1714C > T	Pathogenic	1/758	[35]
		c.1883A > G		3/297/LVH in 2 cases	[38]
	I653T	c.1958T > C	Absent	1/807	[29]
CaM	R759T	c.2276G > C	Pathogenic	1/myectomy	[31]
	R796C	c.2386C > T	Uncertain significance	1/632	[29]

Table S1: Identification and characterisation of ACTN2 missense variants. ACTN2 missense variants were retrieved from the Human Gene Mutation Database (accessed 10/2022) and pathogenicity was predicted as described [5]. Pathogenicity classifications are sourced from ClinVar database (accessed 01/2023). Conflicting interpretations are indicated by #, with the number of studies reporting each interpretation provided in parentheses. Abbreviations: LVH (left ventricular hypertrophy), LVOT (Left ventricular outflow tract obstruction), AFL (atrial flutter), AFib (atrial fibrillation), NYHA I (New York Heart Association heart failure stage 1); ABD: actin-binding domain, CaM, calmodulin-homology domain.

ACTN2 variants	Localisation	Potential Mechanism	Predicted Modelling Outcome
M92V	CH1	Ligand binding	Disrupt actin-binding due to reduced hydrophobic contacts
R93Q	CH1	Ligand binding	Disrupt actin-binding due to loss of polar interactions
G111V	CH1	ABD regulatory function	Reduce CH1loop flexibility which indirectly impacts actin-binding
S147L	CH1-CH2	ABD regulatory function	Reduce interdomain CH1-CH2 loop flexibility which indirectly impacts actin-binding
T247M	CH2	ABD regulatory function	Compromise ABD conformational dynamics
R327C	SR1	Dimerisation	Disrupt ACTN2 dimerisation due to loss of salt-bridge interaction
E448A	SR2	Domain stability	Disrupt SR2 stabilisation due to loss of hydrogen bond interaction
R457C	SR2	Dimerisation	Disrupt ACTN2 dimerisation due to loss of salt-bridge interaction
I653T	SR4	Domain stability	Disrupt SR4 stabilisation due to reduced hydrophobic contacts

Table S2: Summary of the predicted impact of selected HCM-linked missense variants on ACTN2 structure. Results from structural modelling highlighting potential mechanisms of disease pathogenesis. Abbreviations: CH: Calponin-homology domain, SR: spectrin repeat, ABD: actin-binding domain.

	ACTN2-R327C	ACTN2-R457C
(a) Data Collection		
Beamline	I03 (DLS, UK)	I03 (DLS, UK)
Wavelength (Å)	0.976230	0.976269
Resolution range (Å)	60.9-3.70 (4.05-3.70)	181.8-3.70 (3.76-3.70)
Space group	$P\ 2\ 2_1\ 2_1$	$P\ 2\ 2_1\ 2_1$
Cell parameters a, b, c (Å)	73.14, 102.26, 182.68	73.33, 102.70, 181.83
Total Reflections	202,607 (47,448)	197,569 (9,945)
Unique Reflections	15,251 (3,576)	15,303 (725)
Multiplicity	13.3 (13.3)	12.9 (13.7)
Completeness (%)	99.9 (100.0)	100.0 (100.0)
Mean I/σ(I)	6.1 (1.0)	7.9 (0.4)
Wilson B factor (Å^2)	121.1	121.7
R_{merge}	0.246 (2.848)	0.194 (2.863)
$CC_{1/2}$	0.999 (0.766)	1.000 (0.601)
Software	XDS/AIMLESS	DIALS
(b) Refinement		
R_{work} / R_{free} (%)	24.44 / 29.02	24.26 / 29.08
Protein atoms	6,846	6,840
Solvent molecules / other atoms	0	0
B-factor (Å^2) (Protein)	145.21	138.10
(c) Ramachandran Plot		
Favoured (%)	95.04	95.65
Allowed (%)	4.84	4.23
Outliers (%)	0.12	0.12
Rotamer outliers (%)	0.00	0.00
Clash score	23.40	24.58
(d) RMSD		
Bonds (Å) / Angles (°)	0.006 / 1.057	0.006 / 1.002
Software	PHENIX refine / TLS	PHENIX refine / TLS
PDB code	9SIR	9SIS

Table S3: Data collection and refinement statistics of ACTN2-R327C and ACTN2-R457C structures. The highest resolution shell is displayed in parentheses. Abbreviations: DLS: Diamond Light Source, R_{merge}: merging R factor; $CC_{1/2}$: half dataset correlation coefficient; RMSD: root mean square deviation.

		R^2	K_d (μM)	CI	p-value	Δ Affinity	B_{max} (μM)	CI	p-value	Δ Capacity
Actin Binding Assay	WT	0.80	3.75	2.22-6.43	-	-	2.63	2.16-3.37	-	-
	M92V	0.81	13.40	3.24-inf	-	↓	4.49	1.89-inf	-	↑
	R93Q	0.86	2.97	0.85-15.2	-	-	2.22	1.47-5.56	-	-
	G111V	0.53	4.10	1.56-10.6	-	-	1.23	0.88-1.99	-	↓
	S147L	0.82	2.19	0.84-5.86	-	↑	1.25	0.89-1.92	-	↓
	T247M	0.63	1.79	0.85-3.59	-	↑	5.16	4.04-6.88	-	↑
Reciprocal Actin Binding Assay	WT	0.86	1.60	0.97-2.42	-	-	0.59	0.55-0.63	-	-
	G111V	0.61	2.19	0.81-4.54	0.498	-	0.32	0.27-0.37	<0.0001****	↓
	T247M	0.91	0.32	0.08-0.60	0.0002***	↑	0.85	0.82-0.89	<0.0001****	↑

Table S4: summary of actin binding assay parameters for the WT and ACTN2-ABD variants. Parameters include R^2 (best-line fit), K_d (binding affinity constant), CI (confidence interval), p-value, and B_{max} (maximum binding capacity). Arrows indicate a decrease (↓) or increase (↑) in Δ affinity and capacity relative to WT. Data are analysed using a non-linear regression analysis with ***$p < 0.001$ and ****$p < 0.0001$. Values for the actin-binding assay represents multiple individual pulldown experiments over a range of different starting ACTN2 concentrations (0-12μM). Values for the reciprocal actin binding assay represent 3 to 4 independent co-sedimentations at each increasing actin concentration (0-30 μM).

	Frames selected		
Samples	Buffer subtraction	Elution Peak 1 (P1)	Elution Peak (P2)
WT	215-246 and 512-543	300-329	340-366
M92V	207-233 and 511-540	300-329	342-366
R93Q	205-237 and 564-588	262-325	342-363
G111V	201-231 and 572-594	256-340	350-380
T247M	202-223 and 568-589	245-320	343-366
R327C	204-234 and 567-591	263-328	343-370
E448A	216-242 and 573-596	266-334	350-374
R457C	207-237 and 511-543	276-326	340-371

Table S5: Summary of the SEC-SAXS frames selected for buffer extraction and elution peaks (P1 and P2) for ACTN2-WT and ACTN2 variants with SEC-SAXS profiles displayed in Figures 5, S8, and S10.

		ACTN2 variants		Primers (5' to 3')
ABD	1	M92V	FWD	CCCAAACCTGACCGGGGAAAAGTGCGGTTCCAC
			REV	GTGGAACCGCACTTTTCCCCGGTCAGGTTTGGG
	2	R93Q	FWD	CTGACCGGGGAAAAATGCAGTTCCACAAAATTGCTAA
			REV	TTAGCAATTTTGTGGAACTGCATTTTTCCCCGGTCAG
	3	G111V	FWD	TTACATAGCCAGCAAAGTGGTGAAACTGGTGTCCA
			REV	TGGACACCAGTTTCACCACTTTGCTGGCTATGTAA
	4	S147L	FWD	CATCCTTCGCTTTGCTATTCAGGATATTTTGGTTGAAGAAACATC
			REV	GATGTTTCTTCAACCAAAATATCCTGAATAGCAAAGCGAAGGATG
	5	T247M	FWD	GATGAAAGAGCCATCATGATGTACGTCTCTTGCTTCTAC
			REV	GTAGAAGCAAGAGACGTACATCATGATGGCTCTTTCATC
Rod	6	D325Y	FWD	TGGAGGACTTCCGGTATTACCGCCGGAAG
			REV	CTTCCGGCGGTAATACCGGAAGTCCTCCA
	7	R327C	FWD	GACTTCCGGGATTACTGCCGGAAGCACAAG
			REV	CTTGTGCTTCCGGCAGTAATCCCGGAAGTC
	8	R353W	FWD	CTGCAGACCAAGCTGTGGATCAGCAACCGTC
			REV	GACGGTTGCTGATCCACAGCTTGGTCTGCAG
	9	R398H	FWD	TCGGAGACTGGAGCACTTGGAACACCTGG
			REV	CCAGGTGTTCCAAGTGCTCCAGTCTCCGA
	10	E448A	FWD	ACGAGGCGTTCGCGAGCGACCTGGC
			REV	GCCAGGTCGCTCGCGAACGCCTCGT
	11	E448K	FWD	GCACGAGGCGTTCAAGAGCGACCTGGC
			REV	GCCAGGTCGCTCTTGAACGCCTCGTGC
	12	R457C	FWD	GCGCACCAGGACTGCGTGGAGCAGA
			REV	TCTGCTCCACGCAGTCCTGGTGCGC
	13	R572W	FWD	GGCGGACGGAGAGTGGCAGTCCATCAT
			REV	ATGATGGACTGCCACTCTCCGTCCGCC
	14	E628G	FWD	CCTGCAGGAGGGGCTGGCTCGCC
			REV	GGCGAGCCAGCCCCTCCTGCAGG
	15	I653T	FWD	GCCATTGGGCCCTGGACCCAGAACAAGATGG
			REV	CCATCTTGTTCTGGGTCCAGGGCCCAATGGC
CaM	16	R759T	FWD	CCAGGAGCAGATGAATGAGTTCACGGCCTCCTTCAAC
			REV	GTTGAAGGAGGCCGTGAACTCATTCATCTGCTCCTGG
	17	R796C	FWD	GTGAAGCCGAATTTGCCTGCATTATGACCCTGGTA
			REV	TACCAGGGTCATAATGCAGGCAAATTCGGCTTCAC

Table S6: Summary of the forward (FWD) and reverse (REV) primers (5' to 3') used for generating the ACTN2 missense variants.

(*a*) Sample details	
Organism	Homo sapiens
Source	pET23a plasmid (Novagen) [7]
Description	Uniprot ID: P35609 ACTN2_HUMAN, with N-terminal T7 immunotag and C-terminal Hexahistidine-tag
Extinction coefficient ε	127365 M^{-1} cm^{-1} at 280 nm
Partial specific volume $\bar{\upsilon}$	0.734 cm^3g^{-1}
Mean solute and solvent scattering length densities and mean scattering contrast $\Delta\bar{\rho}$	2.876x10^{10} cm^{-2}; 0.734 cm^3g^{-1}
Molecular mass *M* from chemical composition	103,853 Da
Loading concentration, injection volume and flow rate	(SEC-SAXS): concentration: 6-8 mg/mL: injection volume: 50 µL; flow rate: 0.16 mL/min; (Batch-mode SAXS): concentration: 1mg/mL: injection volume: 35 µL; flow rate: 1 µL/s
Concentration measured and method	(SEC-SAXS): concentration: 6-8 mg/mL; (Batch-mode SAXS): concentration: 1mg/mL; NanoDrop spectrophotometer at 280 nm (A280)
Solvent composition and source	20 mM HEPES pH 8, 150 mM NaCl, and 1% glycerol (Sigma-Aldrich)
(*b*) SAXS data collection parameters	
Source, instrument and reference	B21 beamline, Diamond Light Source, Harwell, UK [21]
Wavelength (λ)	λ = 0.94 Å
Beam geometry (size, sample-to-detector distance)	Point source, spherical 50 mm FWHM at detector. Sample-detector distance of 3.7 m
q-measurement range ($Å^{-1}$)	Range of momentum transfer $0.0045 < q < 0.34$ $Å^{-1}$; $q = 4\pi\sin(\theta)/\lambda$
Absolute scaling method	Scale water scatter to 0.0163 cm^{-1}
Basis for normalization to constant counts	Divide by beam intensity reading on an integrating beamstop diode near the detector face
Method for monitoring radiation damage, X-ray dose where relevant	Multiple short exposures taken and checked for systematic increase in aggregation before averaging
Exposure time, number of exposures	(SEC-SAXS): 3 s exposures with flow of 0.075 ml/min; (Batch-mode SAXS): 1s x 21 exposures
Sample configuration including path length and flow rate where relevant	1.5 mm diameter quartz capillary; (Batch-mode SAXS):1 ml/s; (SEC-SAXS): 0.075 mL/min
Sample temperature (ºC)	(SEC-SAXS): room temperature; (Batch-mode SAXS): measurement taken at each of the following temperatures: 40, 47, 53, 55, 57, 60 ºC
(*c*) Software employed for SAXS data reduction, analysis and interpretation	
SAXS data reduction to sample–solvent scattering, and extrapolation, merging, and desmearing	Normalisation, radial integration, application of mask and AgBeh calibration via DAWN [39]
Calculation of ε from sequence	N/A
Calculation of $\Delta\bar{\rho}$ and $\bar{\upsilon}$ values from chemical composition	MULCh ModULes for the analysis of small-angle neutron Contrast variation data from bio-molecular assemblies [40]
Basic analyses: Guinier, *P*(*r*), scattering particle volume	ScÅtter software (v. IV.d, BIOISIS); CHROMIXS [22]; PRIMUS [27]
Shape/bead modelling	DENsity from Solution Scattering (DENSS) software [26]
Atomic structure modelling	N/A
Modelling of missing sequence from PDB files	N/A
Molecular graphics	N/A

(*d*) Structural parameters (See also Tables 2 and 3)									
SEC-SAXS									
Guinier Analysis		WT	M92V	R93Q	G111V	T247M	R327C	E448A	R457C
I(0) (cm^{-1})		-	-	-	-	-	-	-	-
R_g (Å)	Peak 1	120	N/D	N/D	N/D	N/D	N/D	N/D	N/D
	Peak 2	107	105	107	117	118	110	116	113

q-range (Å^{-1})		-	-	-	-	-	-	-	-
Quality-of-fit parameter		-	-	-	-	-	-	-	-
M from I(0)		-	-	-	-	-	-	-	-
$P(r)$ analysis		WT	M92V	R93Q	G111V	T247M	R327C	E448A	R457C
I(0) (cm^{-1})		-	-	-	-	-	-	-	-
R_g (Å)	Peak 1	125	157	180	175	264	157	153	159
	Peak 2	112	112	113	116	117	114	114	112
D_{max} (Å)	Peak 1	410	490	570	555	780	489	481	499
	Peak 2	362	364	370	369	369	360	365	370
q-range (Å^{-1})		-	-	-	-	-	-	-	-
Quality-of-fit parameter		-	-	-	-	-	-	-	-
M from I(0)		-	-	-	-	-	-	-	-
Volume (10^3 Å^3)	Peak 1	865	1,086	1,509	1,648	2,688	1,253	1,125	1,135
	Peak 2	389	372	408	474	457	418	443	379
Batch-mode SAXS									
Guinier Analysis		WT	M92V	R93Q	G111V	T247M	R327C	E448A	R457C
I(0) (cm^{-1})		-	-	-	-	-	-	-	-
R_g (Å)	40 ºC	108	105	108	116	123	113	110	103
	53 ºC	103	103	106	N/D	N/D	103	106	102
	60 ºC	110	N/D	N/D	N/D	N/D	104	110	115
q-range (Å^{-1})		-	-	-	-	-	-	-	-
Quality-of-fit parameter		-	-	-	-	-	-	-	-
M from I(0)		-	-	-	-	-	-	-	-
$P(r)$ analysis		WT	M92V	R93Q	G111V	T247M	R327C	E448A	R457C
I(0) (cm^{-1})		-	-	-	-	-	-	-	-
R_g (Å)	40 ºC	113	114	112	120	120	115	121	120
	53 ºC	112	110	111	145	226	112	121	109
	60 ºC	116	177	169	243	240	113	114	113
D_{max} (Å)	40 ºC	377	383	371	386	379	370	380	370
	53 ºC	377	350	360	430	670	360	360	353
	60 ºC	370	468	492	870	701	340	369	370
q-range (Å^{-1})		-	-	-	-	-	-	-	-
Quality-of-fit parameter		-	-	-	-	-	-	-	-
M from I(0)		-	-	-	-	-	-	-	-
Volume (10^3 Å^3)	40 ºC	406	402	396	487	594	455	421	391
	53 ºC	403	423	419	894	2,396	433	417	410
	60 ºC	567	1,472	1,570	6,685	3,895	682	554	684
(*e*) Shape modelling results (N/A)									
		WT	M92V	R93Q	G111V	T247M	R327C	E448A	R457C
q-range for fitting		-	-	-	-	-	-	-	-
Symmetry/anisotropy assumptions		-	-	-	-	-	-	-	-
Ambiguity measure		-	-	-	-	-	-	-	-
χ^2value/range		-	-	-	-	-	-	-	-

		WT	M92V	R93Q	G111V	T247M	R327C	E448A	R457C
P value, any other quality-of-fit parameters		-	-	-	-	-	-	-	-
Adjustable parameters in the model fit		-	-	-	-	-	-	-	-
Model volume and/or *M* estimate		-	-	-	-	-	-	-	-
Model precision/resolution		-	-	-	-	-	-	-	-
For multiple phase shape models, R_g values and relative phase volumes		-	-	-	-	-	-	-	-
(*f*) Atomistic modelling (N/A)									
		WT	M92V	R93Q	G111V	T247M	R327C	E448A	R457C
Method		-	-	-	-	-	-	-	-
q-range for fitting		-	-	-	-	-	-	-	-
Symmetry assumptions		-	-	-	-	-	-	-	-
Any measures of model precision		-	-	-	-	-	-	-	-
χ^2value/range		-	-	-	-	-	-	-	-
P value, any other quality-of-fit parameters		-	-	-	-	-	-	-	-
Adjustable parameters in the model fit		-	-	-	-	-	-	-	-
Relevant output parameters		-	-	-	-	-	-	-	-
Domain/subunit coordinates and contacts, regions of presumed flexibility as appropriate		-	-	-	-	-	-	-	-
(*g*) Data and model deposition IDs									
SASBDB deposition IDs		WT	M92V	R93Q	G111V	T247M	R327C	E448A	R457C
SEC-SAXS	Peak 1	SASDZJ4	SASDZR5	SASDZP5	SASDZM5	SASDZK5	SASDZH5	SASDZF5	SASDZE5
	Peak 2	SASDZK4	SASDZQ5	SASDZN5	SASDZL5	SASDZJ5	SASDZG5	SASDZD5	SASDZC5
Batch-mode SAXS	40 ºC	SASDZB5	SASDZ85	SASDZ55	SASDZ25	SASDZX4	SASDZU4	SASDZR4	SASDZN4
	53ºC	SASDZA5	SASDZ75	SASDZ45	SASDZZ4	SASDZW4	SASDZT4	SASDZQ4	SASDZM4
	60 ºC	SASDZ95	SASDZ65	SASDZ35	SASDZY4	SASDZV4	SASDZS4	SASDZP4	SASDZL4

Table S7: Summary of SAXS data acquisition, sample details, data analysis, and software used for SEC-SAXS and batch-mode SAXS for ACTN2-WT and tested variants. Abbreviations: FWHM: full width at half maximum; N/D: undetermined. SASBDB: small angle scattering biological data bank [https://www.sasbdb.org/]. Not applicable parameters are referred to as N/A or (-). SAXS parameters are also summarised in table 2 (SEC-SAXS) and table 3 (batch-mode SAXS).

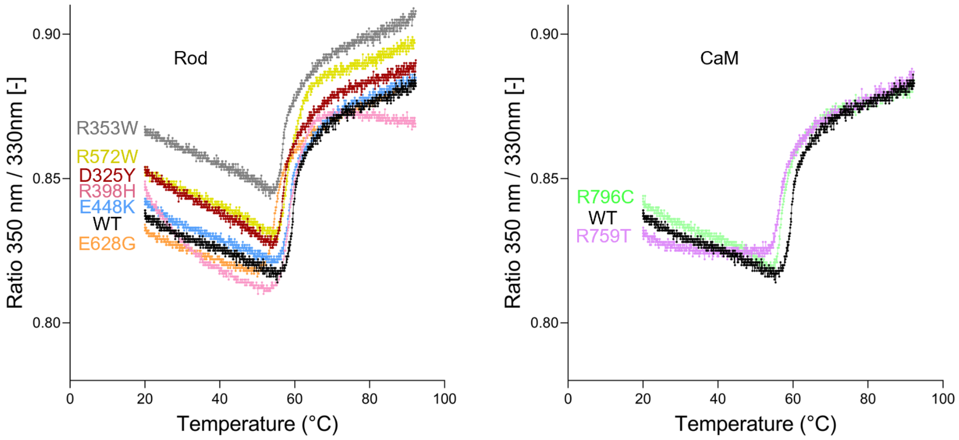

Figure S1: Evaluation of thermal stability of ACTN2 HCM-linked missense variants. Thermal denaturation profiles of six ACTN2 rod variants and two CaM variants using differential scanning fluorimetry showing fluorescence intensity ratios (350 nm/330 nm). Data represents mean values from two independent experimental runs.

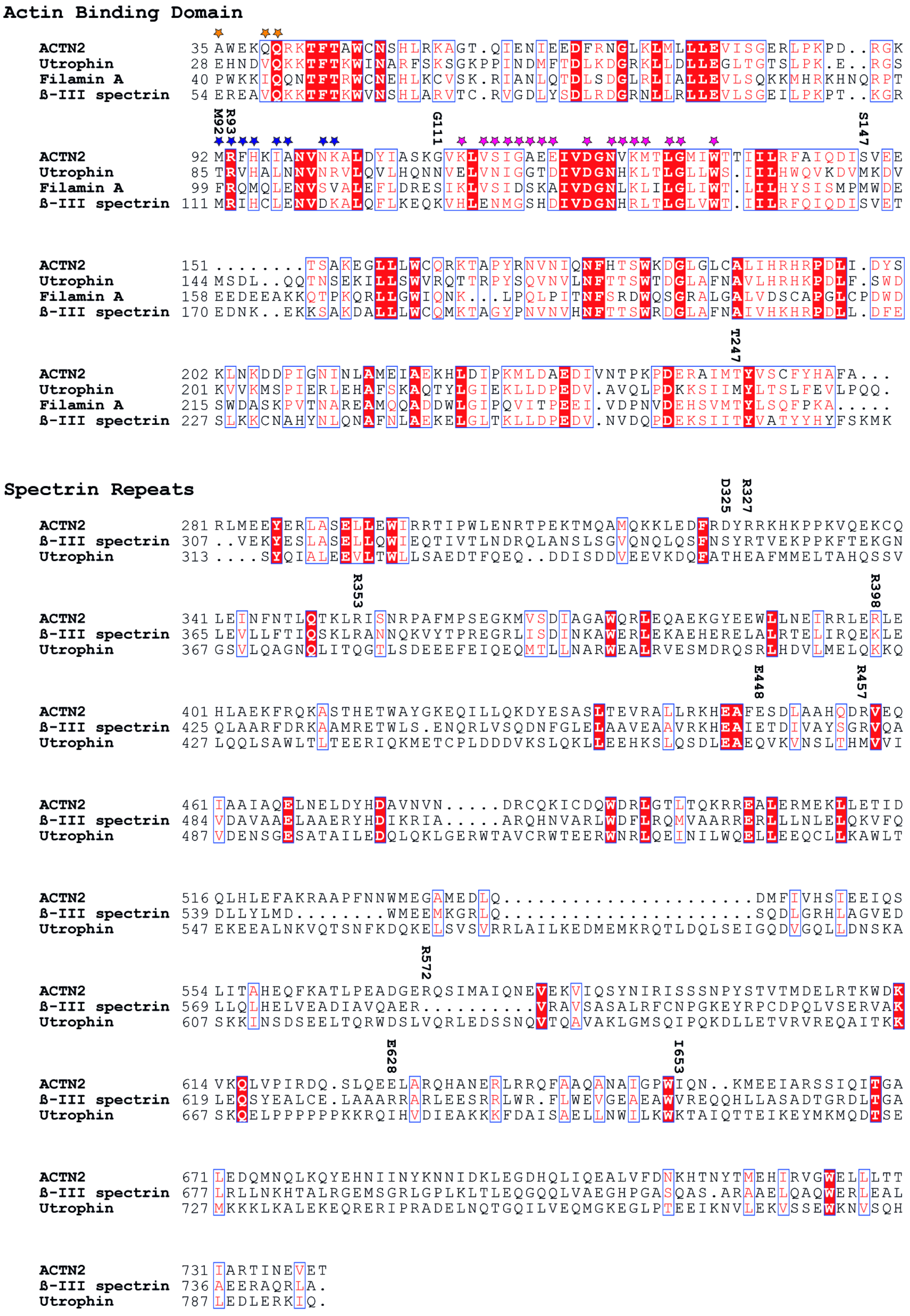

Figure S2: Sequence alignment of actin-binding domains and spectrin repeats across cytoskeletal proteins ACTN2, Utrophin, Filamin A, and β-III spectrin. Protein sequences were obtained from UniProt (accession codes P35609 [ACTN2]; P46939 [Utrophin]; P21333 [Filamin A] and O15020-1 [β-III spectrin]) and aligned using Clustal Omega [41] with default parameters. This figure was generated using Espript [42]. Conserved residues are indicated according to sequence similarity, with identical residues displayed as white characters on a red background and similar residues shown as red characters within blue frames. Hypertrophic cardiomyopathy (HCM)-associated variants in ACTN2 are mapped onto the alignment. ACTN2 residues predicted to bind actin from the ACTN2–actin complex model are indicated by asterisks and cluster into three principal sites: ABD1 (orange), ABD2 (blue), and ABD2′ (magenta).

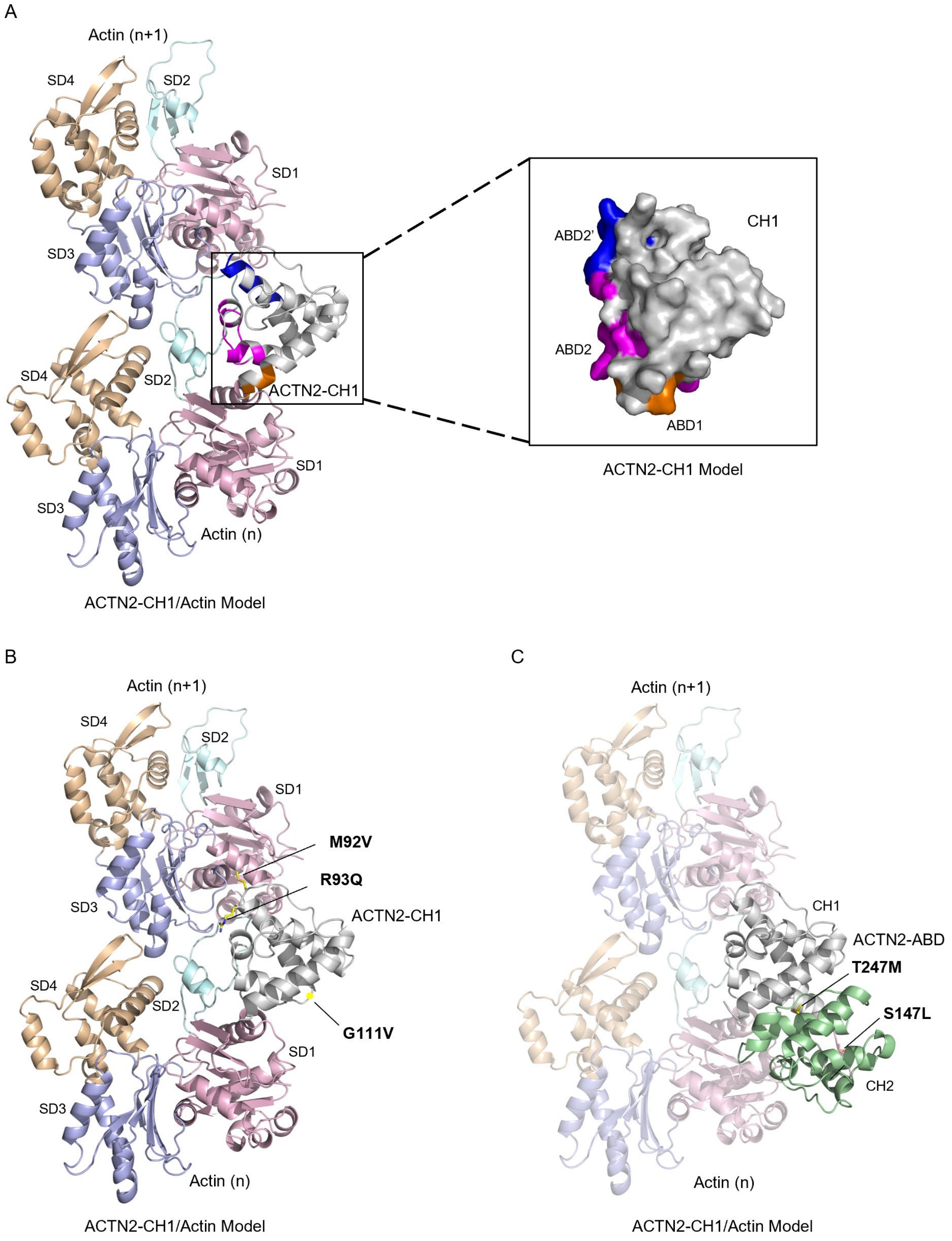

Figure S3: HADDOCK-derived model of the ACTN2-CH1/actin complex with mapped HCM-linked variants. (A) Ribbon diagram showing actin comprised of four subdomains (SD1, light pink; SD2, cyan; SD3, blue; and SD4, wheat). ACTN2-CH1 (grey) engages with the SD1 and SD2 of the nth actin monomer and with the SD1 of the neighboring n+1st actin monomer. Close-up view shows a molecular surface representation of the ACTN2-CH1 domain with three putative actin-binding sites highlighted (ABD1 in orange, ABD2' in blue, and ABD2 in magenta). (B) HCM-associated M92V and R93Q variants are positioned near the putative actin-binding interface and G111V is positioned away from this site. (C) Superimposition of ACTN-ABD onto ACTN2/actin complex reveals that the CH2 domain in its closed state is incompatible with actin binding. HCM-linked S147L and T247M variants are located distal to the putative actin binding site. For clarity, actin monomers are shown in transparent representation, with ACTN2-CH1 domain shown in grey, and ACTN2-CH2 domain in light green.

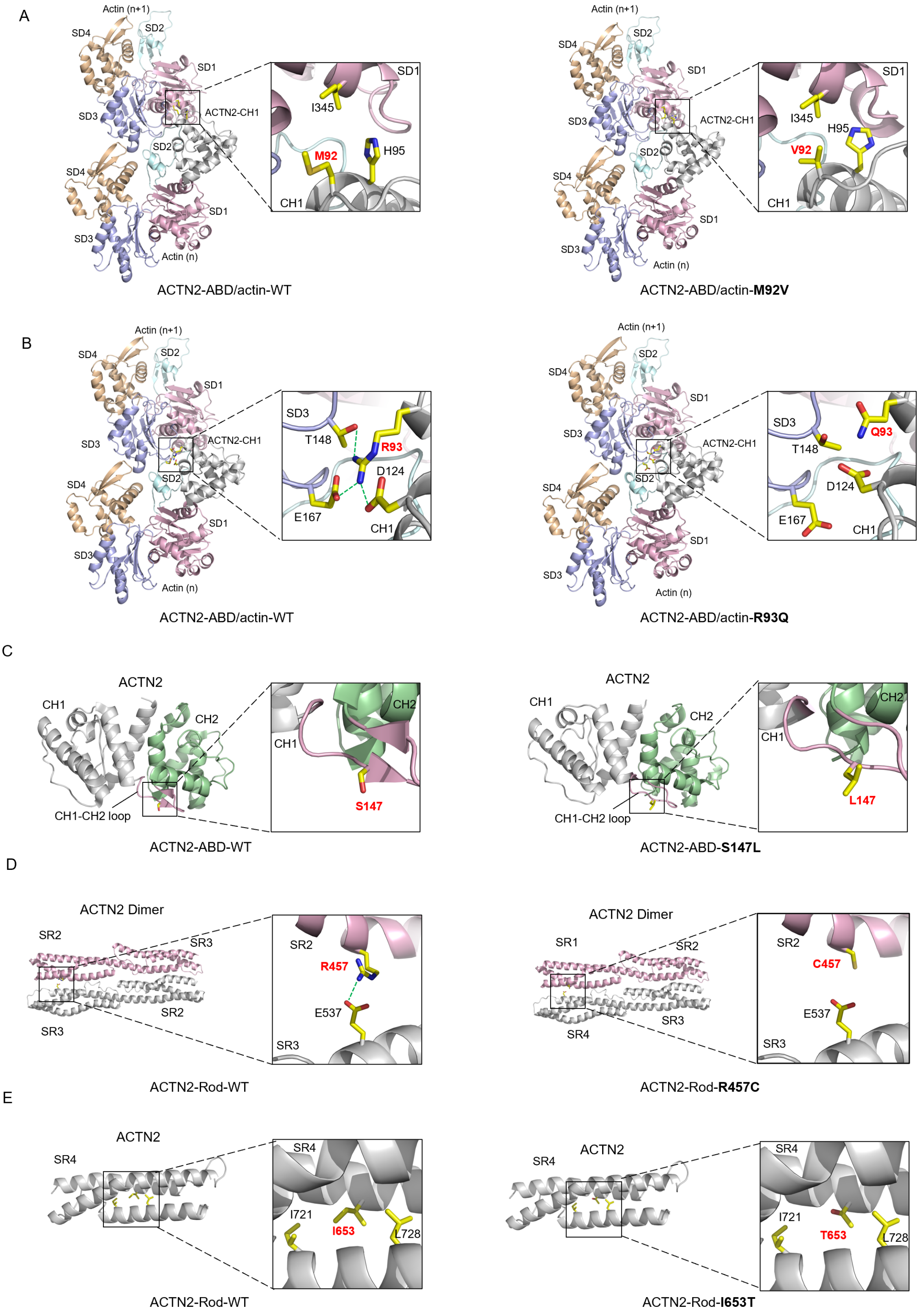

Figure S4: Probing the impact of the ACTN2-ABD variants (M92V, R93Q, S147L), and ACTN2-Rod variants (R457C and I653T) on ACTN2 structure. (A) Ribbon diagram of the HADDOCK-derived molecular model of ACTN2-CH1 domain (grey) in complex with actin (colours as in Figure. S3). M92 forms hydrophobic interactions with residue I345 from actin (left panel). Introduction of V92 is likely to mediate less contacts, potentially disrupting the binding interface (right panel). (B) R93 forms a hydrogen bond interaction with T148

of actin, and salt bridge interaction with E167 of actin and D124 of ACTN2-CH1 (left panel). Introduction of Q93 is predicted to disrupt these interactions (right panel). (C) S147 maps to the interdomain loop connecting CH1 and CH2 domains (in light pink) (left panel). Introduction of L147 may restrict interdomain linker flexibility (right panel). (D) Ribbon diagram illustrating that the ACTN2 dimer interface is stabilised by a salt bridge with R457 of monomer A (light pink) and E537 of monomer B (light grey) (left panel). Introduction of C457 is likely to result in a loss of this interaction (right panel). (E) Ribbon diagram of ACTN2 demonstrating that I653 mediates multiple non-polar interactions with hydrophobic SR4 core residues (I721 and L728) (left panel). (c) Introduction of the polar T653 variant may destabilise the SR4 core region (right panel). For clarity, specific protein structural domains are displayed. Green dashed lines illustrate salt bridges or hydrogen bond interactions. Residue substitutions are highlighted in red. Boxes highlight close-up views of relevant interactions.

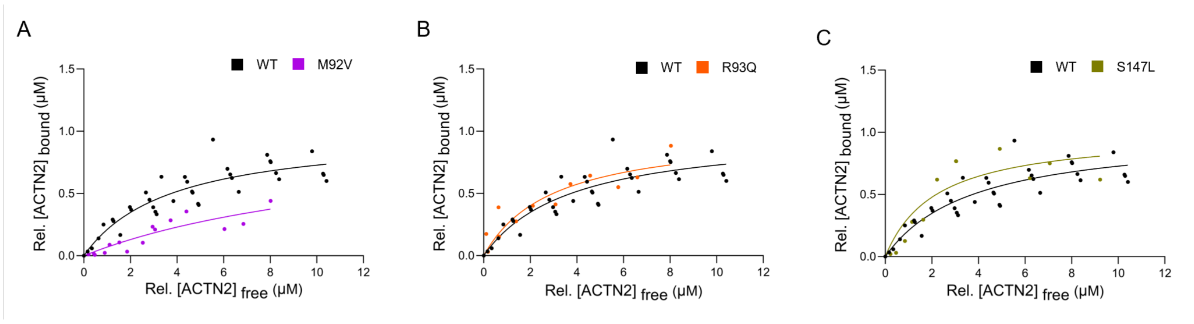

Figure S5: Actin binding assays evaluating the impact of three ACTN2-ABD variants on actin-binding affinity. Rabbit skeletal muscle actin (3 µM) was co-sedimented with varying concentrations of the wild type (WT) protein (0.25-12µM) and the variant proteins M92V, R93Q and S147L (0.25-10µM). (A) M92V variant shows decreased binding affinity. (B) R93Q shows no changes in binding affinity. (C) S147L variant shows increased binding affinity. A nonlinear regression analysis was used to determine binding affinity constant (K_d) and maximum binding capacity (B_{max}). Graphs display measurements after normalisation to the B_{max} value of each variant. Table S4 summarise K_d, B_{max}, Confidence Intervals, R^2 values, Δ affinity and Δ capacity for these variants. Each data point represents individual pulldown experiments over a range of different starting ACTN2 concentrations.

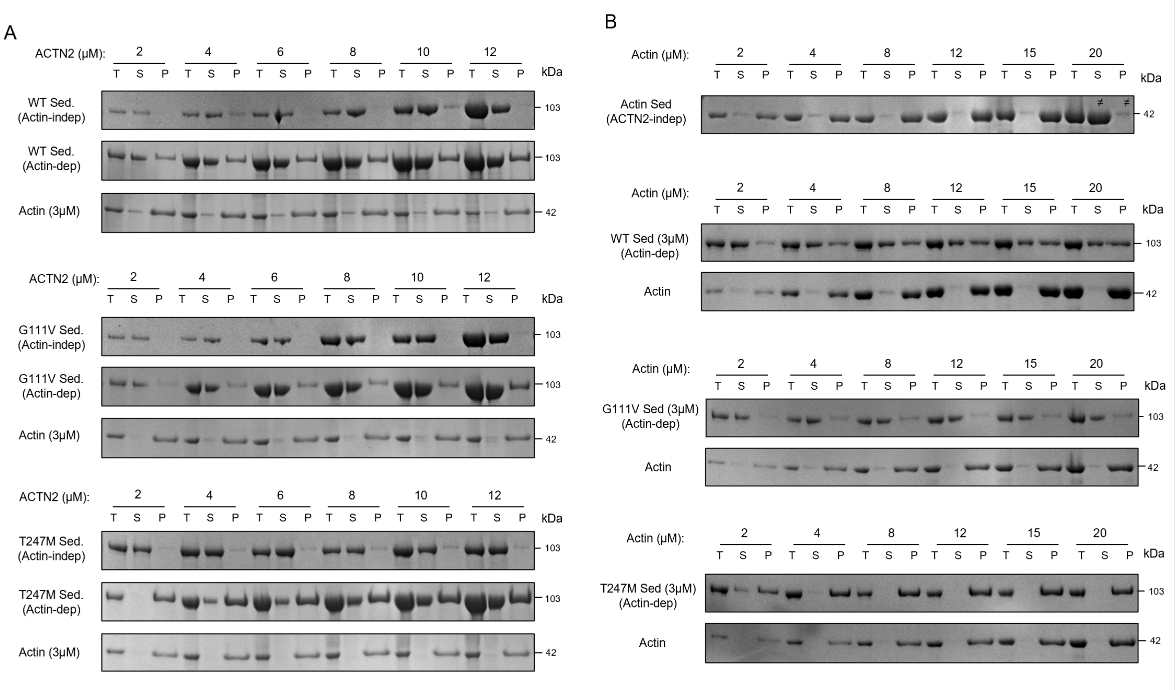

Figure S6: Representative gels for actin-binding assays. (A) Actin-binding assay using 3 µM of actin with increasing concentrations of ACTN2 (2-12 µM) showing ACTN2 sedimentation without and with actin for WT (1st panel), G111V (2nd panel) and T247M (3rd panel). (B) Reciprocal actin-binding assay using 3 µM of ACTN2 with increasing concentrations of actin (2-20 µM) showing actin sedimentation independent of ACTN2 (1st panel) with ≠ denoting a swap of actin supernatant and pellet samples at 20 µM at the point of gel loading. The second panel shows ACTN2 sedimentation (actin-dependent) for WT, G111V (3rd panel) and T247M (4th panel). Abbreviations: T: total protein; S: supernatant; P: pellet; Actin-dep: actin-dependent; Actin-indep: actin-independent.

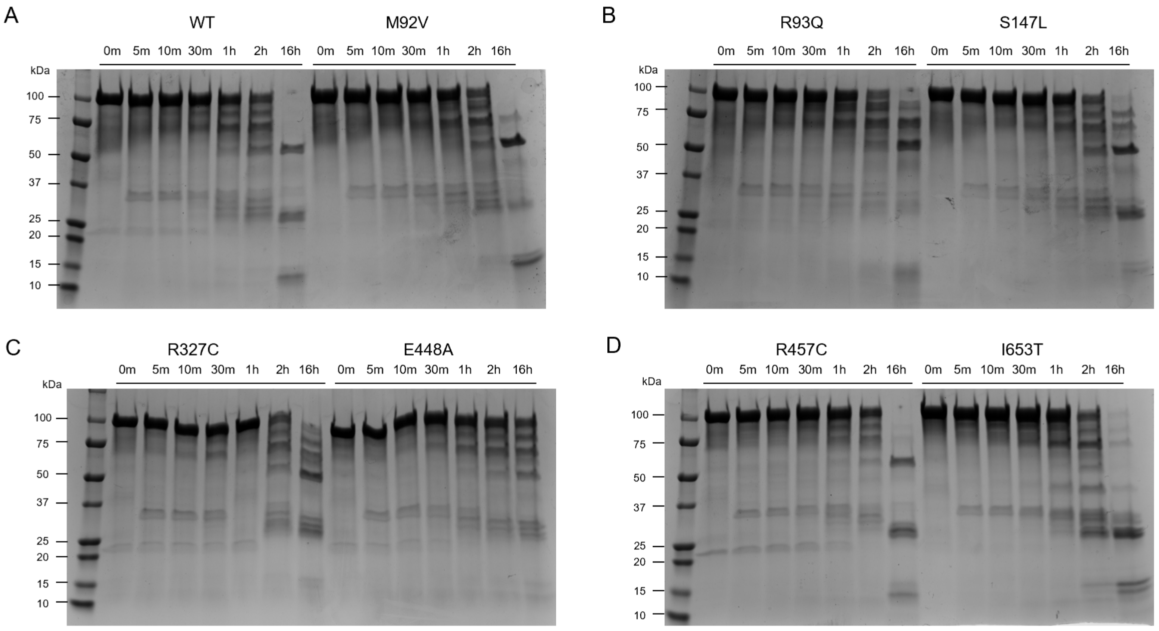

Figure S7: Representative gels for thermolysin (only) digestion in ACTN2-ABD and rod variants. (A, B) Proteolytic digestion of ACTN2-WT and ABD variants (M92V, R93Q, S147L) performed using thermolysin incubation at 37°C, with an enzyme-to-substrate molar ratio of 1:7 (Thermolysin: ACTN2). (C, D) Thermolysin proteolytic digestion of ACTN2 rod variants (R327C, E448A, R457C, I653T). Reactions were stopped at specific time points (5, 10, 30 minutes and 1, 2, 16 hours). Molecular weight markers (kDa) are indicated. Each gel represents one independent replicate.

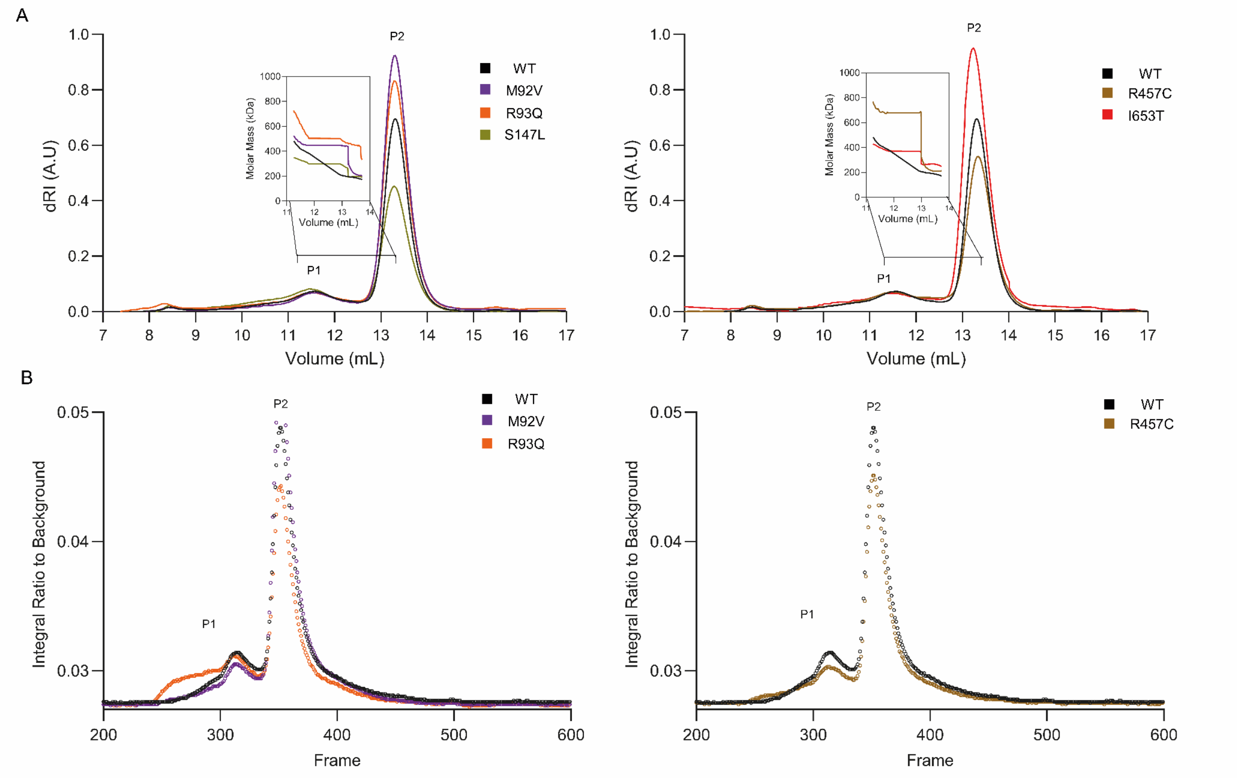

Figure S8: Structural stability assessment of ACTN2 variants. (A) Differential refractive index (dRI) profiles using size-exclusion chromatography coupled with multi-angle light scattering (SEC-MALS) showing ACTN2 variants eluting in two peaks (P1-P2) [M92V, R93Q, S147L (left panel); R457C, I653T (right panel)]. Inset panels display measured molar mass of ACTN2 variants in kDa. SEC-MALS chromatograms of UV absorbance (UV) and light scattering (LS) are displayed in Figure S9. (B) Size exclusion chromatography coupled with small-angle X-ray scattering (SEC-SAXS) showing protein elution profile as two peaks (P1-P2) with P1 displaying aggregate formation in M92V, R93Q, and R457C variants. SEC-SAXS traces represent data points from one independent experimental run. Analysed parameters are summarised in Table 2. Information on frames selected for eluted peaks and background subtraction are summarised in Table S5 and Figure S10. Log_{10} SAXS plots and Guinier analysis of P1 and P2 are displayed in Figures S11 and S12. DENSS-generated electron density models of P2 are displayed in Figure S13. SEC-MALS and SEC-SAXS traces represent data points from one independent experimental run.

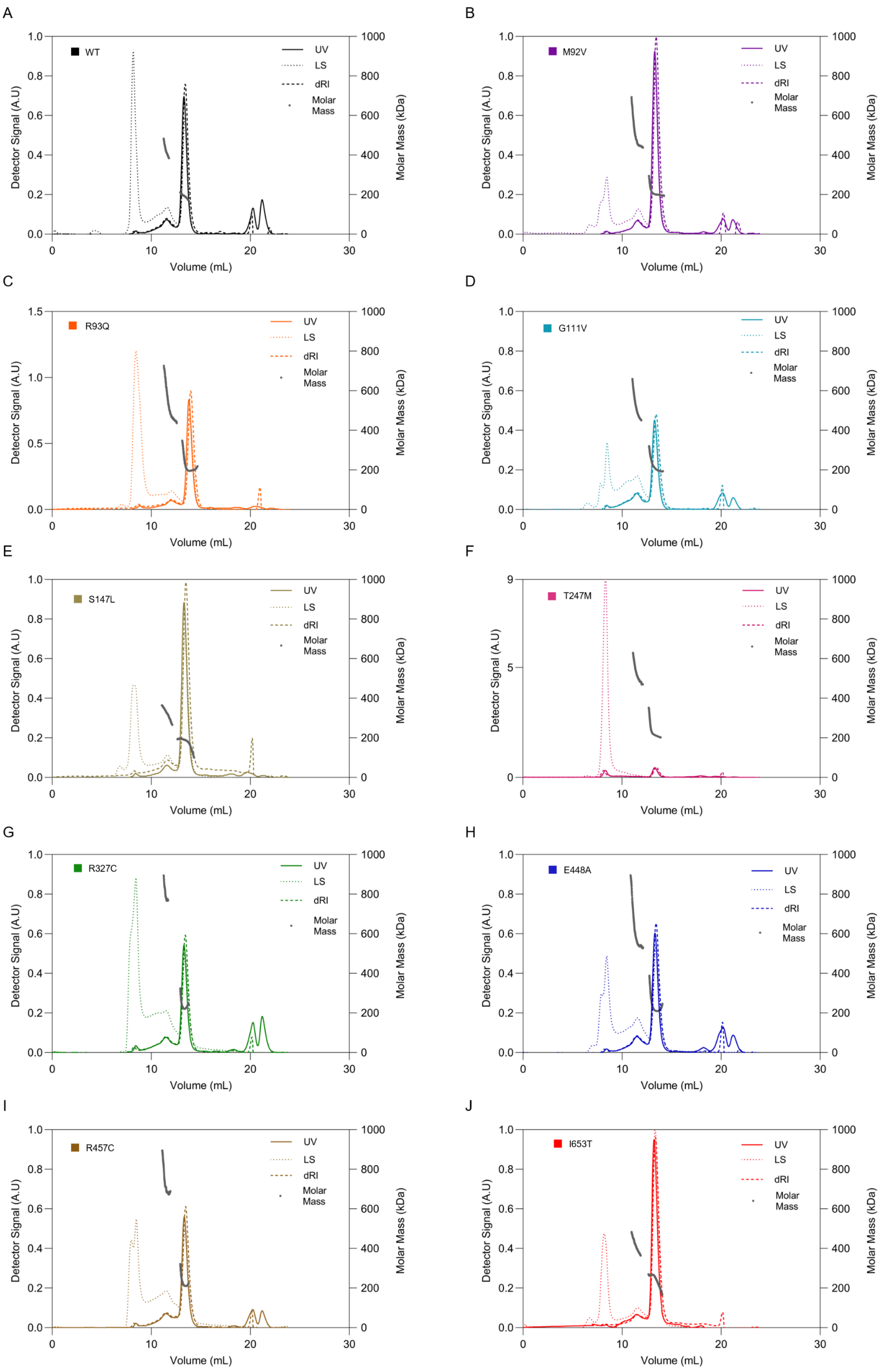

Figure S9: Size-exclusion chromatography coupled with multi-angle light scattering (SEC-MALS). Traces show UV absorbance (UV), light scattering (LS), differential refractive index (dRI) chromatograms, and molar mass across the elution profiles of (A) WT and ACTN2 Variants: (B) M92V, (C) R93Q, (D) G111V, (E) S147L, (F) T247M, (G) R327C, (H) E448A, (I) R457C, (J) I653T. SEC-MALS traces represent data points from one independent experimental run.

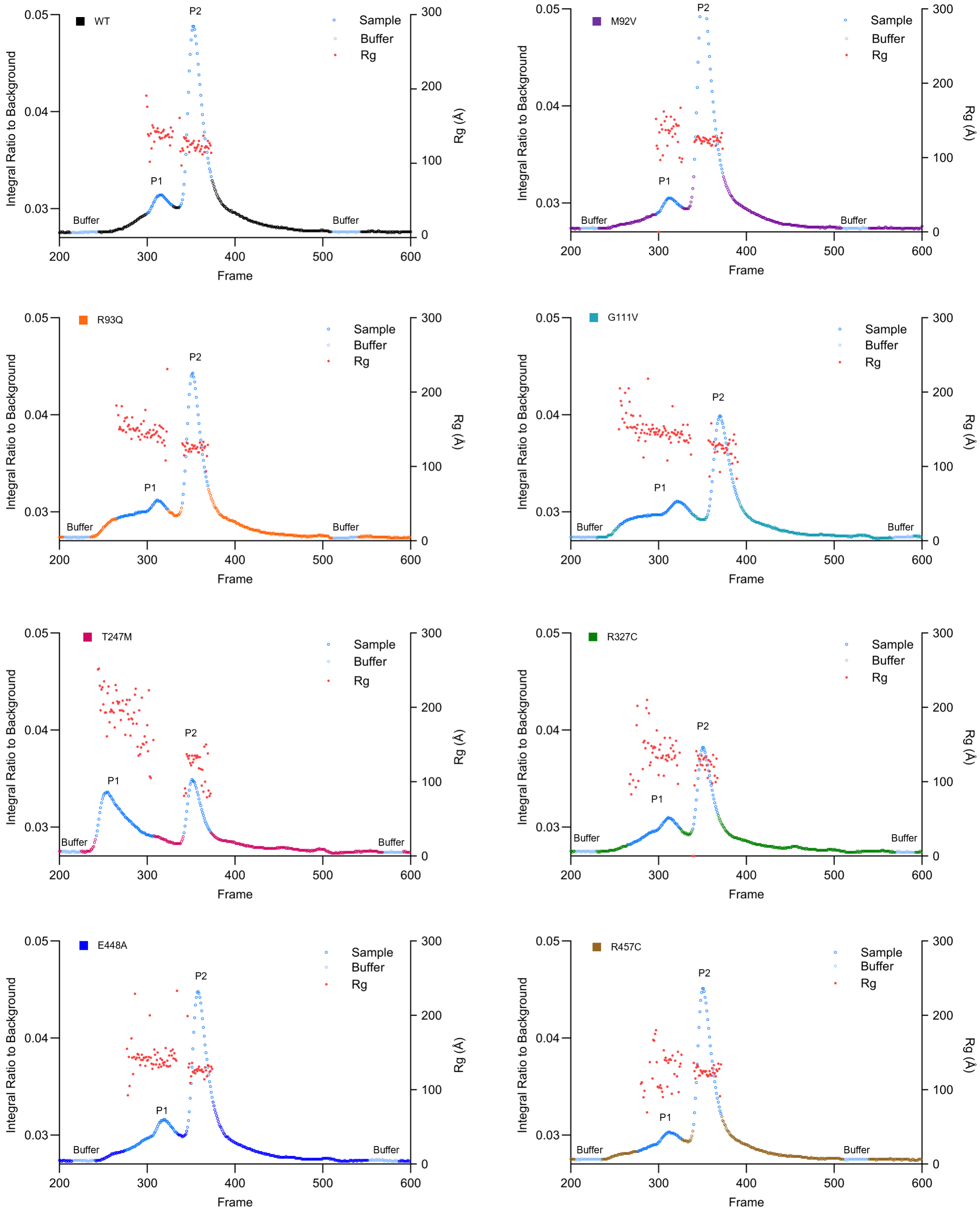

Figure S10: SEC-SAXS traces of WT and ACTN2 variants showing frames selected and R_g values. SEC-SAXS traces were extracted using CHROMIXS [22] showing selected background frames (in light blue), sample frames for P1 and P2 (in blue) and Rg (in red) across the elution peaks. SEC-SAXS traces represent data points from one independent experimental run.

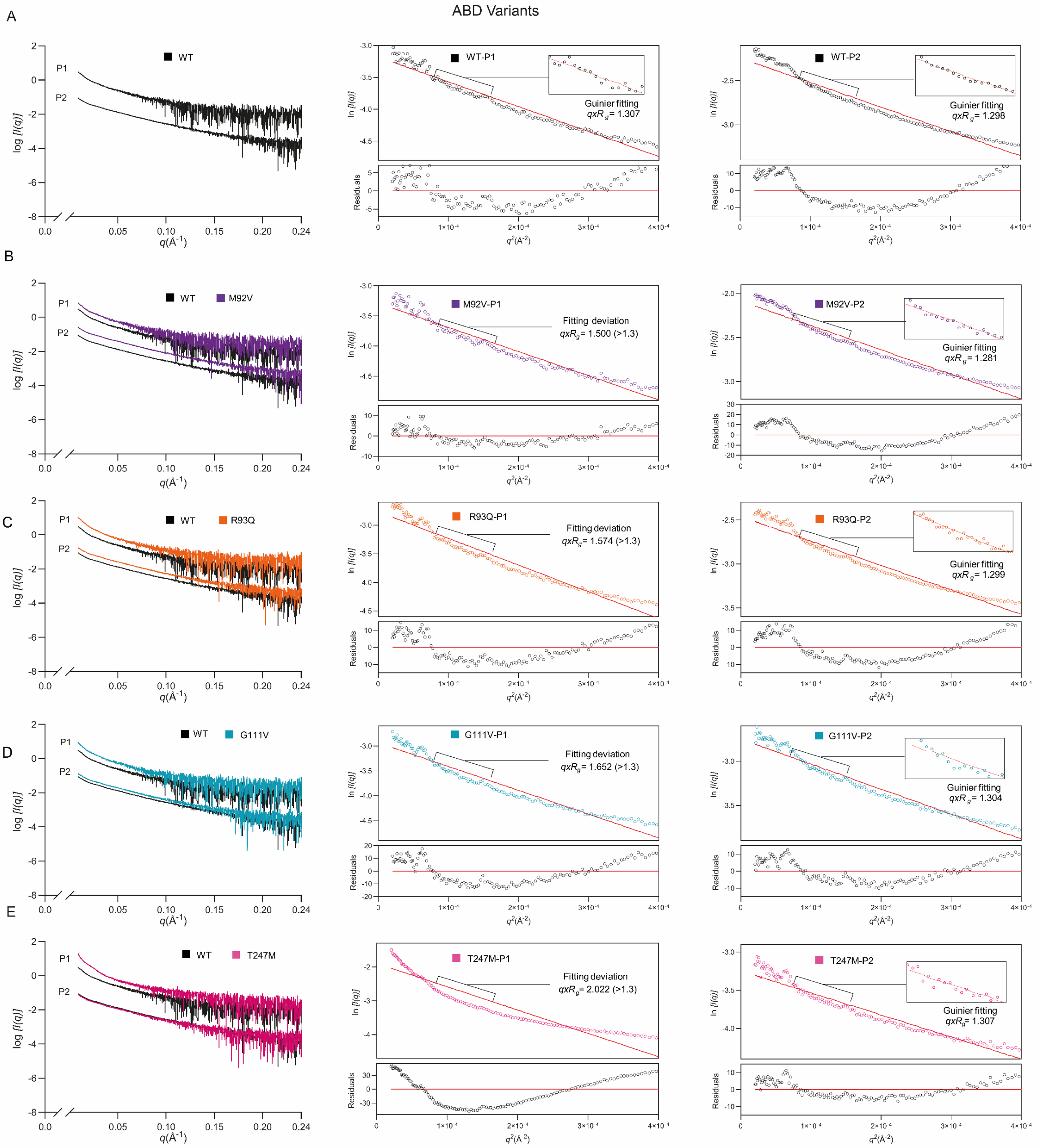

Figure S11: Assessment of ACTN2-ABD variants using size-exclusion chromatography coupled with small-angle X-ray scattering (SEC-SAXS). (A; left panel) ACTN2-WT $\log_{10}$ SAXS intensity vs. scattering vector *(q)* of SEC-SAXS peak 1 and peak 2 (P1-P2). (A; middle and right panels) non-linear dependence of scattering intensity *I(q)* plotted as ln*[I(q)]* vs. q^2 of P1 and P2, respectively, with upper right insets showing Guinier fitting with Guinier approximation of ($q \cdot Rg < 1.3$) and linear fit region highlighted in red. (B, C, D, E, left panels) $\log_{10}$ SAXS intensity of M92V, R93Q, G111V and T247M, respectively, showing P1 and P2 profiles with T247M displaying shift in P1 plot. (B, C, D, E, middle and right panels) data fitting of M92V, R93Q, G111V, and T247M at P1 and P2 with fitting deviation in Guinier approximation of ($q \cdot Rg > 1.3$) at P1. SEC-SAXS traces represent data points from one independent experimental run.

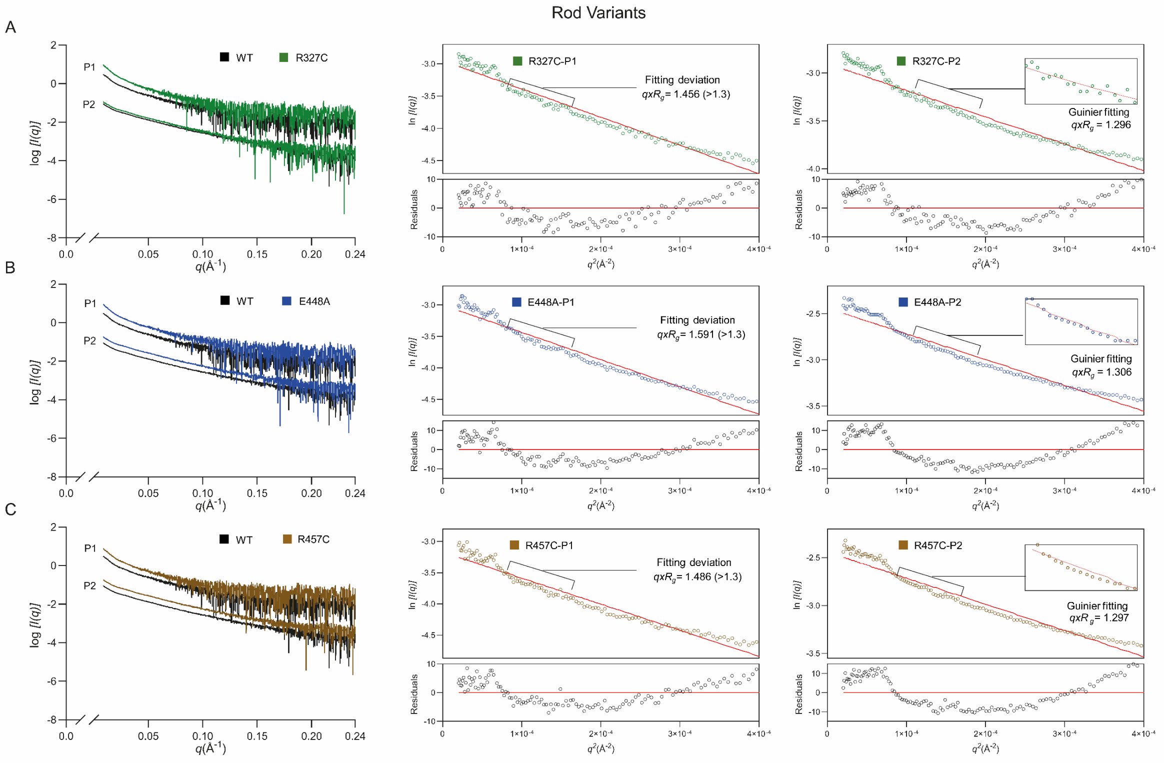

Figure S12: Assessment of ACTN2 rod variants SEC-SAXS P1 and P2. (A, B, C; left panels) $\log_{10}$ SAXS intensity vs. scattering vector *(q)* of SEC-SAXS peak 1 and peak 2 (P1-P2) of R327C, E448A and R457C. (A, B, C; middle and right panels) non-linear dependence of scattering intensity *I(q)* plotted as ln*[I(q)]* vs. q^2 of P1 and P2, respectively, showing fitting deviation in Guinier approximation of ($q{\cdot}Rg > 1.3$) in P1 and Guinier fitting with Guinier approximation of ($q{\cdot}Rg < 1.3$) in P2 (upper insets) with linear fit region highlighted in red. SEC-SAXS traces represent data points from one independent experimental run.

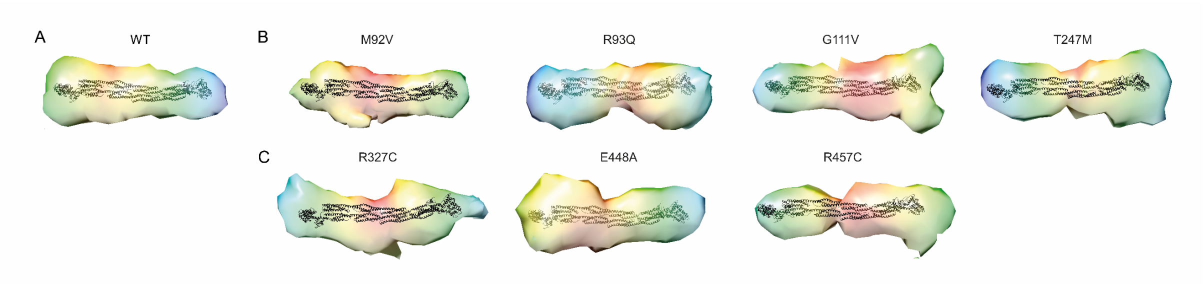

Figure S13: DENsity from Solution Scattering (DENSS)-generated models of SEC-SAXS elution data of peak 2 (P2). (A) DENSS-reconstruction of ACTN2-WT with electron density map showing as 3D colourful transparent volume aligned with the ACTN2 crystal structure (PDB ID: 4D1E) as ribbon diagram (black). (B,C) DENSS-models of ACTN2-ABD and Rod variants, respectively, showing maps similar to WT. DENSS models are generated using the software's default configuration [26].

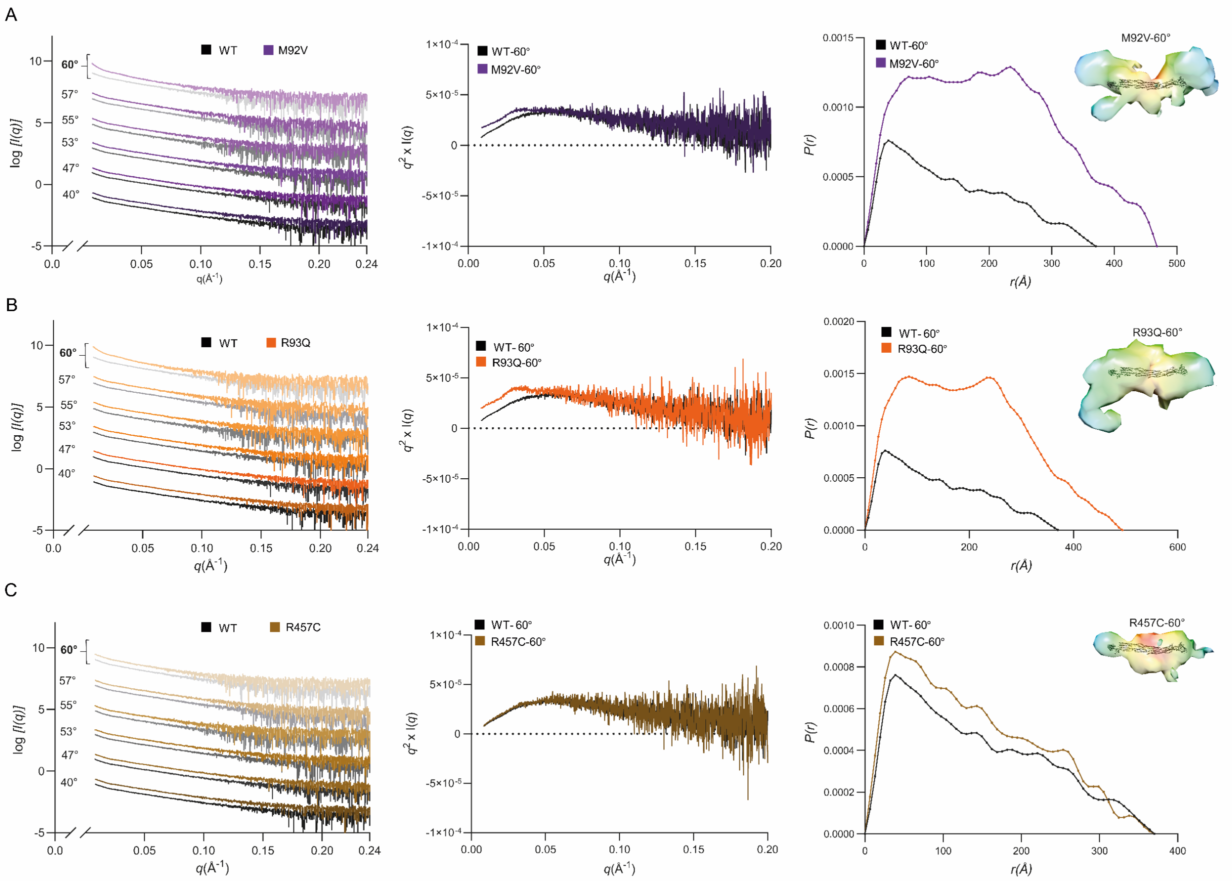

Figure S14: Thermal stability assessment of ACTN2 variants using batch-mode small-angle X-ray scattering (SAXS) with increasing temperature. (A, B; left panel) ACTN2 variants M92V and R93Q respective $\log_{10}$ SAXS intensity vs. scattering vector *(q)* from 40 to 60°C, showing a shift at 60°C compared to ACTN2-WT (in black). (A, B; middle panel) Kratky analysis of M92V and R93Q plotted range represents q^2. I*(q)* versus scattering vector *(q)*. (A, B; right panel) Pair-distance distribution function *P(r)* of M92V and R93Q at 60°C, showing increased maximum particle dimension *(D_{max});* insets (upper right) illustrate aggregation in the SAXS electron density model generated using DENSS [26]. (C) Variant R457C shows no change in $\log_{10}$ intensity plots (left panel), Kratky analysis (middle panel), or D_{max} values and DENSS models (right panel) compared to WT. SAXS traces represent data points from one independent experimental run at each measured temperature.

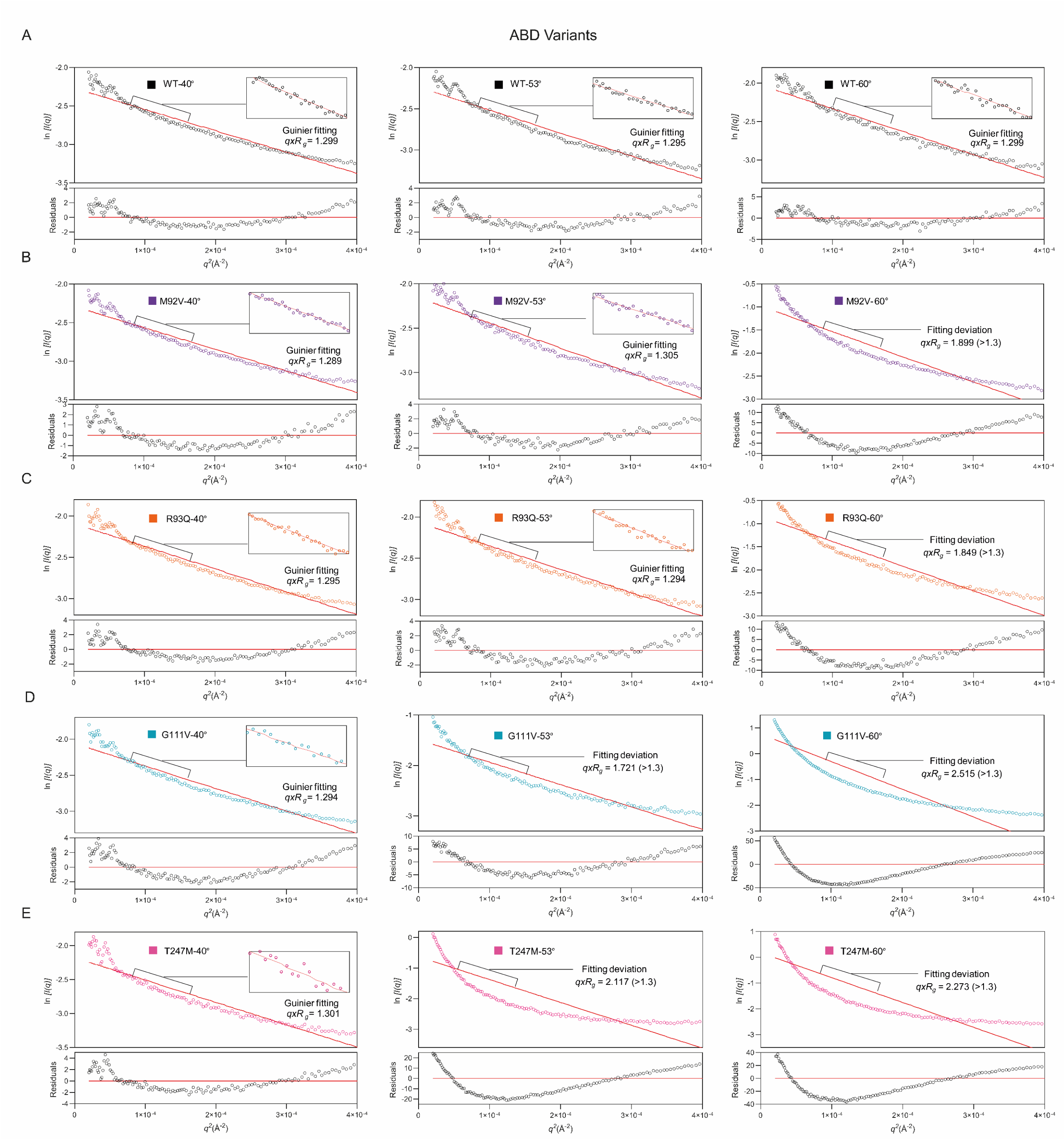

Figure S15: A non-linear dependence of scattering intensity *I(q)* plotted as *ln[I(q)]* vs. q^2 with upper right insets showing Guinier fitting with linear fit region highlighted in red for WT and ABD variants. (A, B, C) Data fitting with Guinier approximation of ($q{\cdot}Rg < 1.3$) of WT, M92V, and R93Q at 40 and 53°C (left and middle panels, respectively) and ($q{\cdot}Rg > 1.3$) at 60°C for M92V and R93Q (right panel). (D, E) Data fitting of G111V and T247M at 40, °C (left panels) with fitting deviation in Guinier approximation of ($q{\cdot}Rg > 1.3$) at 53° and 60°C (middle and right panels, respectively). Analysed data are from one independent experimental run at each measured temperature.

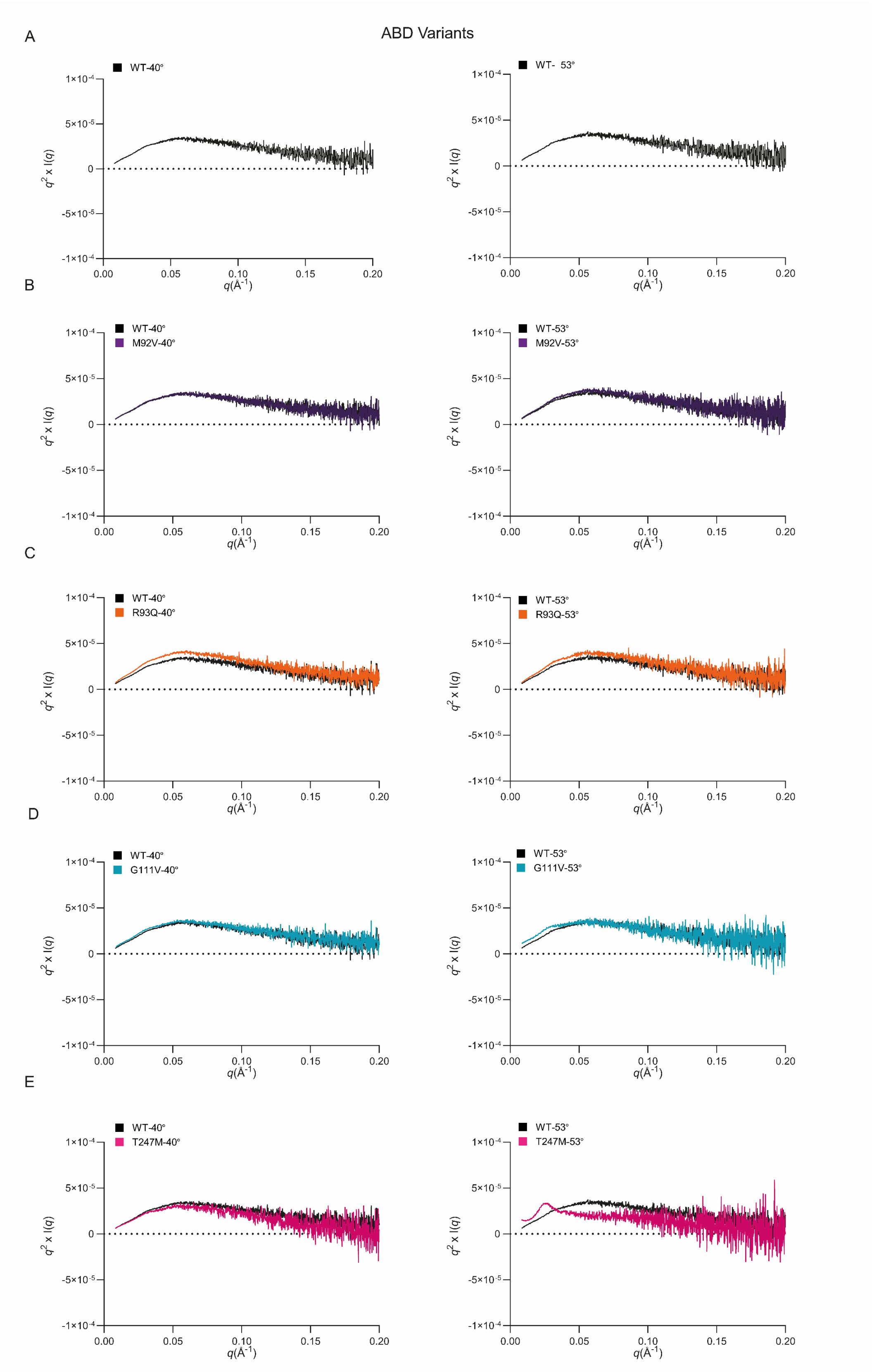

Figure S16: Kratky analysis of ACTN2-ABD variants using batch-mode small-angle X-ray scattering (SAXS). (A) Kratky analysis of ACTN2-WT with plotted range representing q^2. I*(q)* versus scattering vector *(q)* at 40°C (left panel) and 53°C (right panel). (B, C, D, E) Kratky analysis of M92V, R93Q, G111V and T247M at 40°C (left panels) and 53°C (right panels) with analysis showing a shift at 53°C for G111V and T247M. Analysed data are from one independent experimental run at each measured temperature.

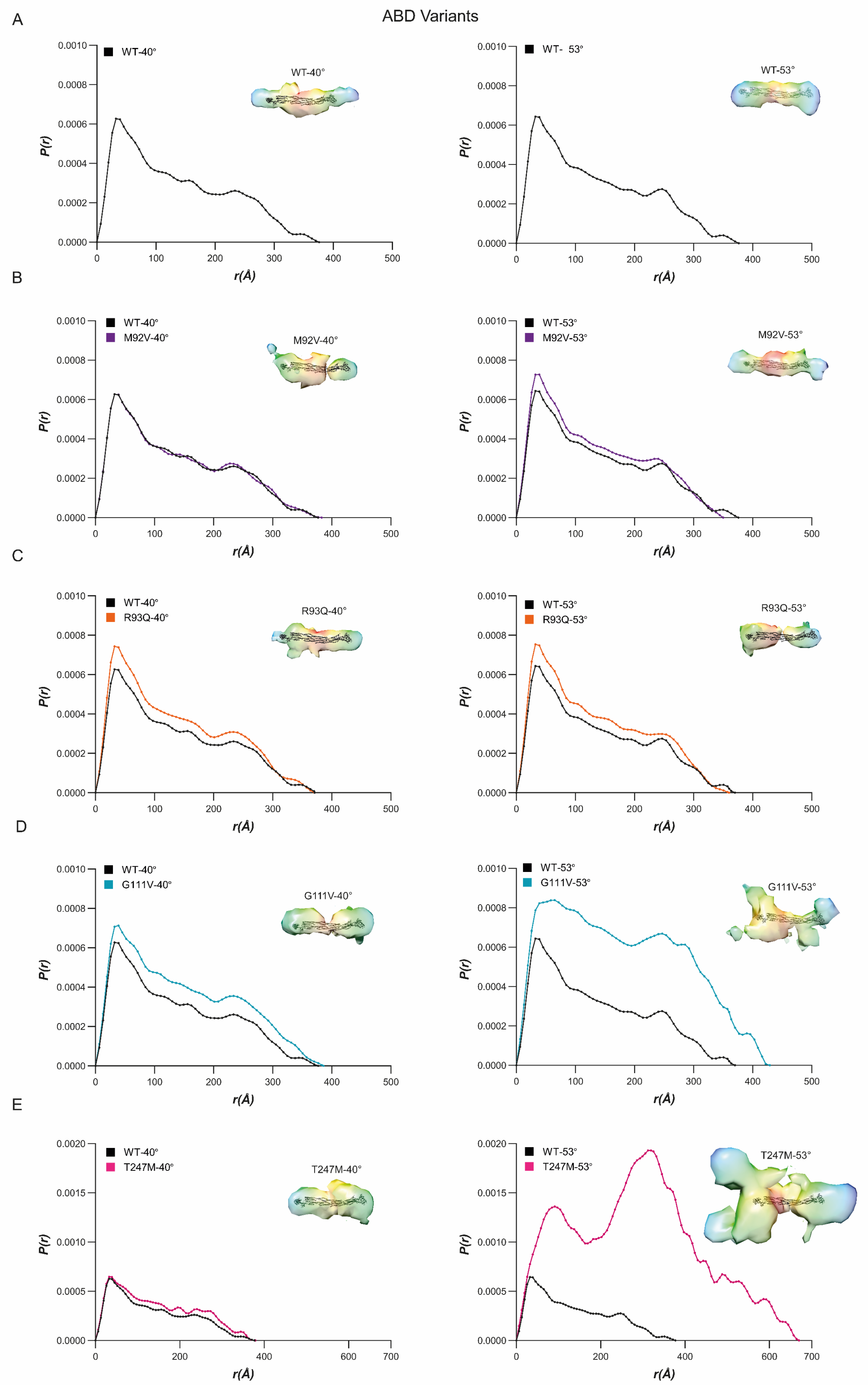

Figure S17: Pair-distance *P(r)* distribution function and DENSS models of WT and ABD variants at 40 and 53°C. (A) *P(r)* distance distribution of WT at 40°C (left panel) and 53°C (right panel), with upper right insets displaying SAXS electron density models generated using DENSS. (B, C) Variants M92V and R93Q reveal no changes in D_{max} and absence of aggregation at both 40 and 53°C. (D, E) G111V and T247M variants display no changes at 40°C (left panel) but increased D_{max} values and evidence of aggregation in DENSS models at 53°C (right panel). Analysed data are from one independent experimental run at each measured temperature.

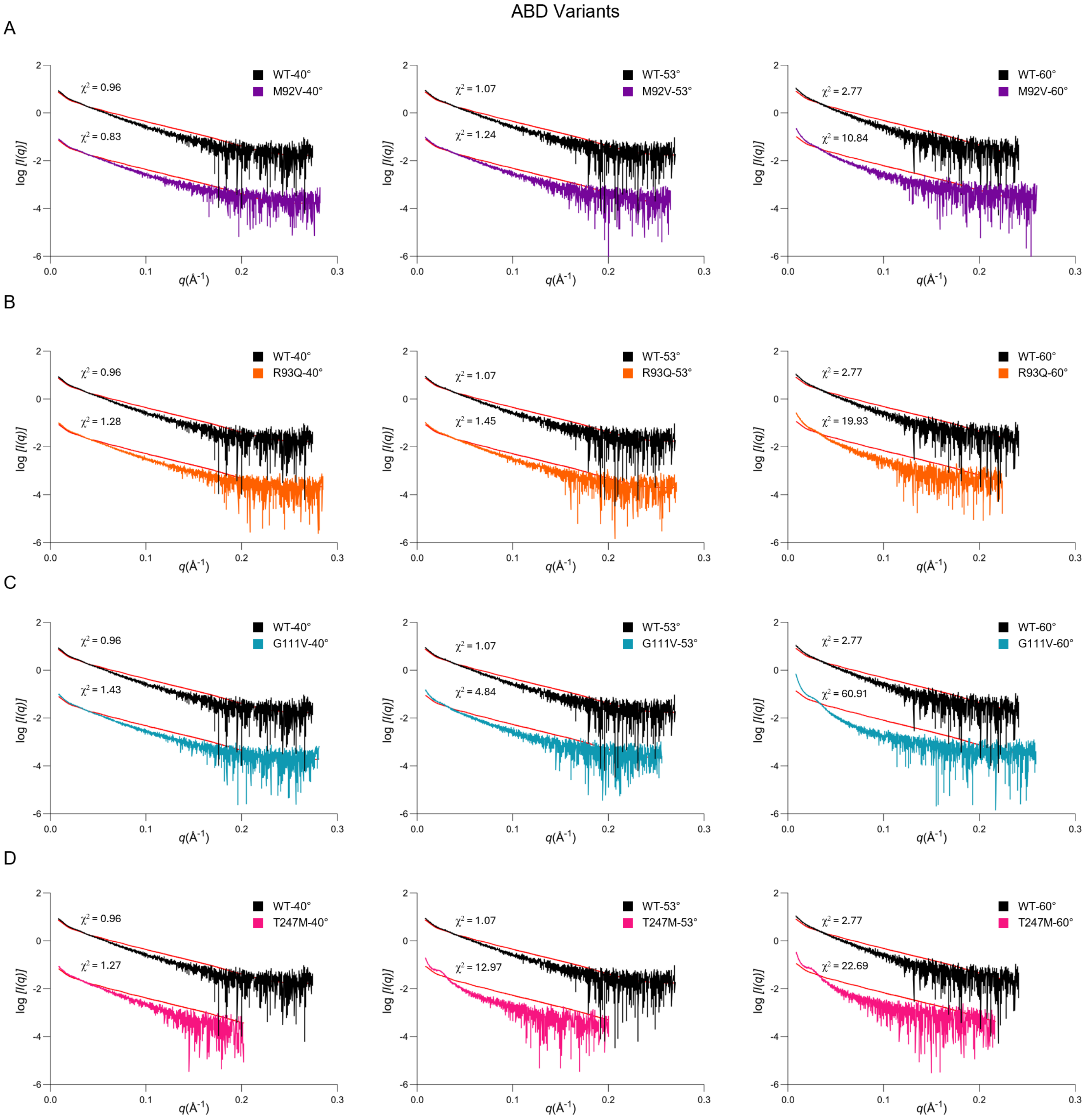

Figure S18: Fitting of fast SAXS profile computation algorithm (FoXS/Fast X-ray Scattering) of ACTN2 X-ray crystal structure (PDB: 4D1E) (in red) to experimental SAXS data of WT and ACTN2-ABD variants. (A, B) FoXS SAXS fit data of M92V and R93Q variants at 40°C and 53°C compared to WT (left and middle panels), while data at 60°C shows a poor fit at low *q* (right panel). (C, D) FoXS SAXS fit data of G111V and T247M variants at 40°C (left panel), and data at 53 and 60°C demonstrates a poor fit at low *q* (middle and right panels). Analysed data are from one independent experimental run at each measured temperature.

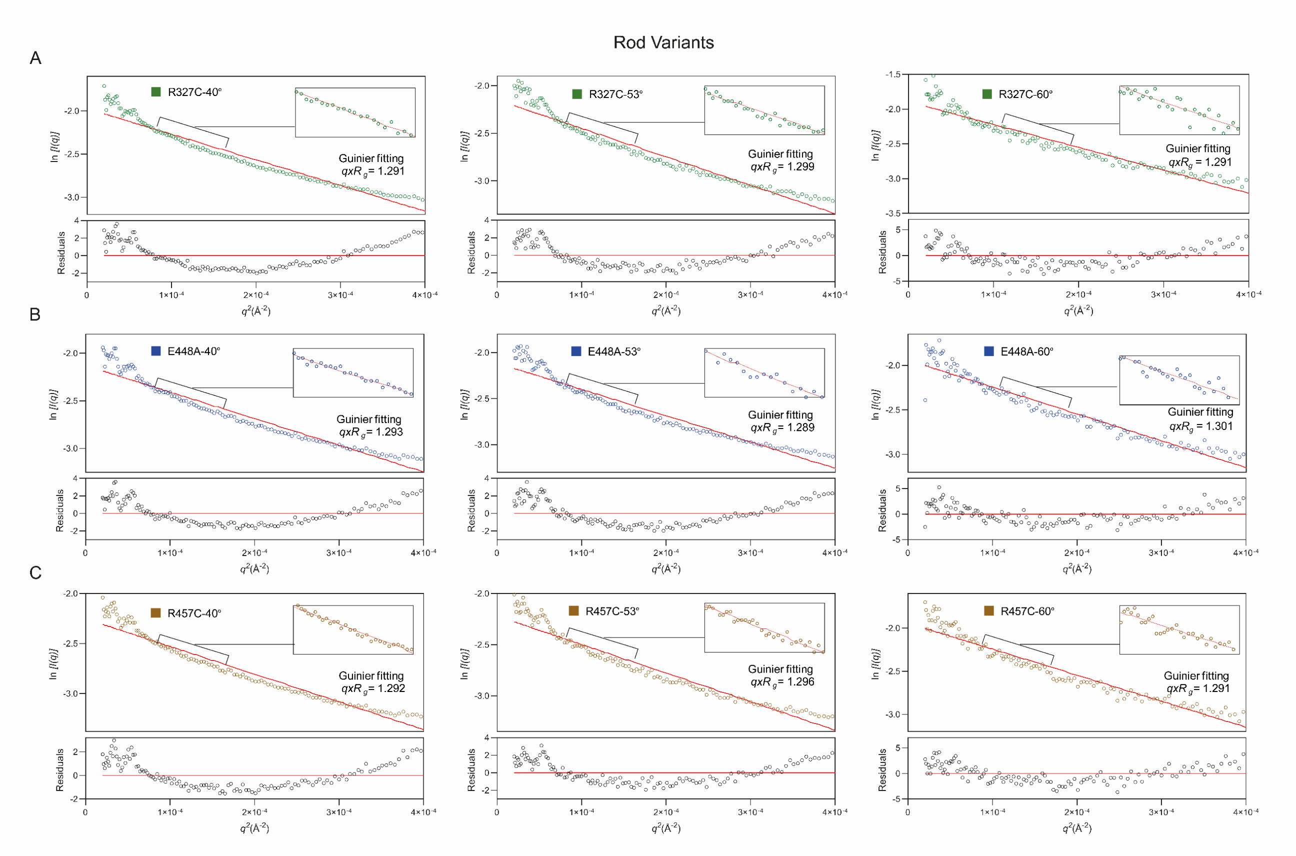

Figure S19: *ln[I(q)]* vs. *q²* plot with upper right insets showing Guinier fitting with linear fit region highlighted in red for rod-domain variants. (A, B, C) Data fitting of R327C, E448A, and R457C with Guinier approximation of ($q{\cdot}Rg < 1.3$) at 40, 53, and 60°C (left, middle, and right panels, respectively). Analysed data are from one independent experimental run at each measured temperature.

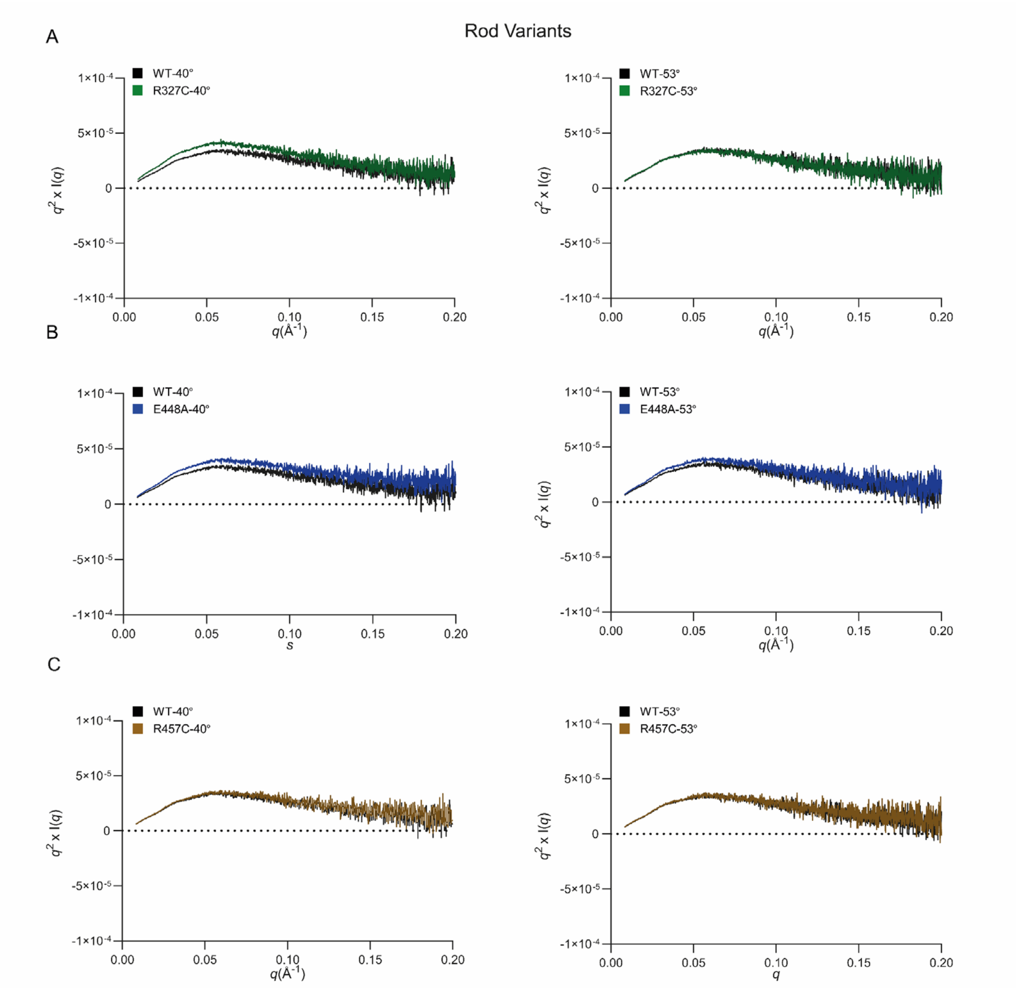

Figure S20: Kratky analysis of ACTN2 rod variants using batch-mode SAXS. (A, B, C) Kratky analysis of R327C, E448A, and R457C with plotted range representing q^2. *I(q)* versus scattering vector *(q)* at 40°C (left panels) and 53°C (right panels) showing no shifts compared to ACTN2-WT (in black). Analysed data are from one independent experimental run at each measured temperature.

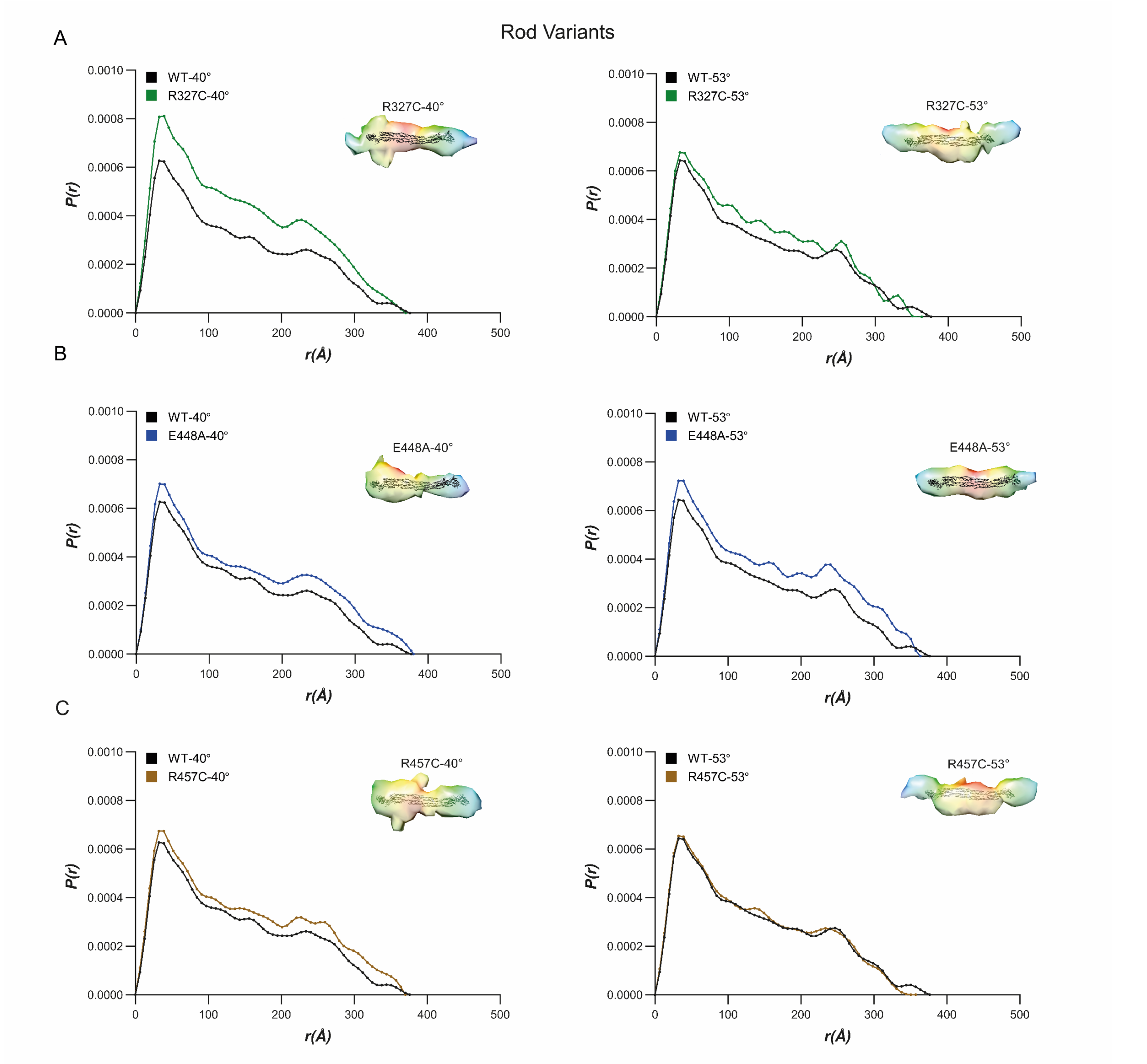

Figure S21: Assessment of pair-distance distribution function *P(r)* and SAXS DENSS models of rod-domain variants at 40 and 53°C. (A, B, C) *P(r)* distance distribution of R327C, E448A and R457C variants shows no change in D_{max} and DENSS models at 40°C (left panel) and 53°C (right panel) compared to WT (in black). Analysed data are from one independent experimental run at each measured temperature.

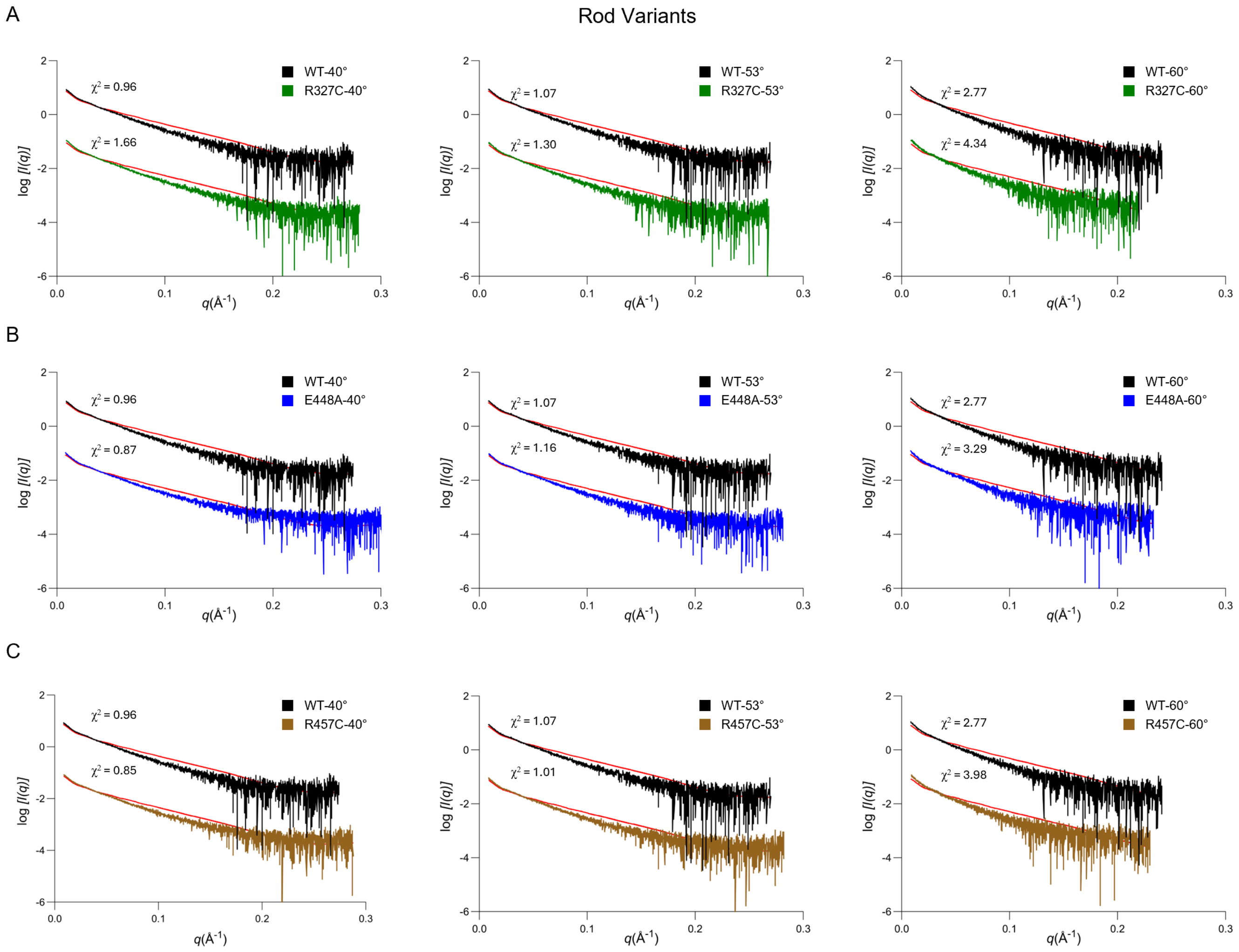

Figure S22: Fitting of ACTN2 experimental data (PDB: 4D1E) (in red) to experimental SAXS data of WT and rod-domain ACTN2 variants using FoXS SAXS. (A, B, C) FoXS SAXS fit data of R327C, E448A, and R457C variants at 40, 53, and 60°C are comparable to WT (left, middle and right panels, respectively). Analysed data are from one independent experimental run at each measured temperature.

Supplementary References:

1. Stenson, P.D., et al., *The Human Gene Mutation Database (HGMD(®)): optimizing its use in a clinical diagnostic or research setting.* Hum Genet, 2020. **139**(10): p. 1197-1207.
2. Gudmundsson, S., et al., *Variant interpretation using population databases: Lessons from gnomAD.* Hum Mutat, 2022. **43**(8): p. 1012-1030.
3. Sim, N.L., et al., *SIFT web server: predicting effects of amino acid substitutions on proteins.* Nucleic Acids Res, 2012. **40**(Web Server issue): p. W452-7.
4. Adzhubei, I., D.M. Jordan, and S.R. Sunyaev, *Predicting functional effect of human missense mutations using PolyPhen-2.* Curr Protoc Hum Genet, 2013. **Chapter 7**: p. Unit7.20.
5. Noureddine, M., et al., *Structural and functional insights into α-actinin isoforms and their implications in cardiovascular disease.* J Gen Physiol, 2025. **157**(2).
6. Landrum, M.J., et al., *ClinVar: improving access to variant interpretations and supporting evidence.* Nucleic Acids Res, 2018. **46**(D1): p. D1062-d1067.
7. Gehmlich, K., et al., *Decreased interactions of mutant muscle LIM protein (MLP) with N-RAP and alpha-actinin and their implication for hypertrophic cardiomyopathy.* Cell Tissue Res, 2004. **317**(2): p. 129-36.
8. Kelley, L.A., et al., *The Phyre2 web portal for protein modeling, prediction and analysis.* Nature Protocols, 2015. **10**(6): p. 845-858.
9. Ribeiro Ede, A., Jr., et al., *The structure and regulation of human muscle α-actinin.* Cell, 2014. **159**(6): p. 1447-60.
10. Fealey, M.E., et al., *Dynamics of Dystrophin's Actin-Binding Domain.* Biophys J, 2018. **115**(3): p. 445-454.
11. de Vries, S.J., M. van Dijk, and A.M.J.J. Bonvin, *The HADDOCK web server for data-driven biomolecular docking.* Nature Protocols, 2010. **5**(5): p. 883-897.
12. Kumari, A., et al., *Structural insights into actin filament recognition by commonly used cellular actin markers.* Embo j, 2020. **39**(14): p. e104006.
13. Agirre, J., et al., *The CCP4 suite: integrative software for macromolecular crystallography.* Acta Crystallogr D Struct Biol, 2023. **79**(Pt 6): p. 449-461.
14. Walter, T.S., et al., *Lysine methylation as a routine rescue strategy for protein crystallization.* Structure, 2006. **14**(11): p. 1617-22.
15. Vagin, A. and A. Teplyakov, *Molecular replacement with MOLREP.* Acta Crystallogr D Biol Crystallogr, 2010. **66**(Pt 1): p. 22-5.
16. Afonine, P.V., et al., *Towards automated crystallographic structure refinement with phenix.refine.* Acta Crystallogr D Biol Crystallogr, 2012. **68**(Pt 4): p. 352-67.
17. Emsley, P., et al., *Features and development of Coot.* Acta Crystallogr D Biol Crystallogr, 2010. **66**(Pt 4): p. 486-501.
18. Krissinel, E. and K. Henrick, *Secondary-structure matching (SSM), a new tool for fast protein structure alignment in three dimensions.* Acta Crystallogr D Biol Crystallogr, 2004. **60**(Pt 12 Pt 1): p. 2256-68.
19. Pardee, J.D. and J.A. Spudich, *Purification of muscle actin.* Methods Enzymol, 1982. **85 Pt B**: p. 164-81.
20. Innis, R.B., et al., *Consensus nomenclature for in vivo imaging of reversibly binding radioligands.* J Cereb Blood Flow Metab, 2007. **27**(9): p. 1533-9.
21. Cowieson, N.P., et al., *Beamline B21: high-throughput small-angle X-ray scattering at Diamond Light Source.* J Synchrotron Radiat, 2020. **27**(Pt 5): p. 1438-1446.
22. Panjkovich, A. and D.I. Svergun, *CHROMIXS: automatic and interactive analysis of chromatography-coupled small-angle X-ray scattering data.* Bioinformatics, 2018. **34**(11): p. 1944-1946.
23. Basham, M., et al., *Data Analysis WorkbeNch (DAWN).* J Synchrotron Radiat, 2015. **22**(3): p. 853-8.
24. Rambo, R.P. and J.A. Tainer, *Characterizing flexible and intrinsically unstructured biological macromolecules by SAS using the Porod-Debye law.* Biopolymers, 2011. **95**(8): p. 559-71.
25. Grant, T.D., et al., *The accurate assessment of small-angle X-ray scattering data.* Acta Crystallogr D Biol Crystallogr, 2015. **71**(Pt 1): p. 45-56.

26. Grant, T.D., *Ab initio electron density determination directly from solution scattering data.* Nat Methods, 2018. **15**(3): p. 191-193.
27. Manalastas-Cantos, K., et al., *ATSAS 3.0: expanded functionality and new tools for small-angle scattering data analysis.* J Appl Crystallogr, 2021. **54**(Pt 1): p. 343-355.
28. Schneidman-Duhovny, D., et al., *Accurate SAXS profile computation and its assessment by contrast variation experiments.* Biophys J, 2013. **105**(4): p. 962-74.
29. Walsh, R., et al., *Reassessment of Mendelian gene pathogenicity using 7,855 cardiomyopathy cases and 60,706 reference samples.* Genet Med, 2017. **19**(2): p. 192-203.
30. Marschall, C., A. Moscu-Gregor, and H.G. Klein, *Variant panorama in 1,385 index patients and sensitivity of expanded next-generation sequencing panels in arrhythmogenic disorders.* Cardiovasc Diagn Ther, 2019. **9**(Suppl 2): p. S292-s298.
31. Theis, J.L., et al., *Echocardiographic-determined septal morphology in Z-disc hypertrophic cardiomyopathy.* Biochem Biophys Res Commun, 2006. **351**(4): p. 896-902.
32. Kim, H.Y., et al., *Genotype-Related Clinical Characteristics and Myocardial Fibrosis and their Association with Prognosis in Hypertrophic Cardiomyopathy.* J Clin Med, 2020. **9**(6).
33. Prondzynski, M., et al., *Disease modeling of a mutation in α-actinin 2 guides clinical therapy in hypertrophic cardiomyopathy.* EMBO Mol Med, 2019. **11**(12): p. e11115.
34. Micheu, M.M., et al., *Yield of Rare Variants Detected by Targeted Next-Generation Sequencing in a Cohort of Romanian Index Patients with Hypertrophic Cardiomyopathy.* Diagnostics (Basel), 2020. **10**(12).
35. Burns, C., et al., *Multiple Gene Variants in Hypertrophic Cardiomyopathy in the Era of Next-Generation Sequencing.* Circ Cardiovasc Genet, 2017. **10**(4).
36. Chung, H., et al., *Effect of sarcomere and mitochondria-related mutations on myocardial fibrosis in patients with hypertrophic cardiomyopathy.* J Cardiovasc Magn Reson, 2021. **23**(1): p. 18.
37. Lu, C., et al., *Molecular analysis of inherited cardiomyopathy using next generation semiconductor sequencing technologies.* J Transl Med, 2018. **16**(1): p. 241.
38. Chiu, C., et al., *Mutations in alpha-actinin-2 cause hypertrophic cardiomyopathy: a genome-wide analysis.* J Am Coll Cardiol, 2010. **55**(11): p. 1127-35.
39. Filik, J., et al., *Processing two-dimensional X-ray diffraction and small-angle scattering data in DAWN 2.* J Appl Crystallogr, 2017. **50**(Pt 3): p. 959-966.
40. Whitten, A.E., S. Cai, and J. Trewhella, *MULCh: modules for the analysis of small-angle neutron contrast variation data from biomolecular assemblies.* Journal of Applied Crystallography, 2008. **41**(1): p. 222-226.
41. Madeira, F., et al., *The EMBL-EBI Job Dispatcher sequence analysis tools framework in 2024.* Nucleic Acids Res, 2024. **52**(W1): p. W521-w525.
42. Robert, X., C. Guillon, and P. Gouet, *FoldScript: a web server for the efficient analysis of AI-generated 3D protein models.* Nucleic Acids Research, 2025. **53**(W1): p. W277-W282.
