# Supplementary material for: Comprehensive biophysical and structural profiling of alpha-actinin-2 variants reveals mechanistic diversity in hypertrophic cardiomyopathy
Source: Nat Commun. 2026 Jul 21;17:6127. doi: 10.1038/s41467-026-75392-z (PMC13388997; doi:10.1038/s41467-026-75392-z)
Supplement: Supplementary file 2 — Reporting Summary [file 41467_2026_75392_MOESM2_ESM.pdf]

nature portfolio

Corresponding author(s): Maya Noureddine
Fiyaz Mohammed
Katja Gehmlich

Last updated by author(s): May 18, 2026

Reporting Summary

Nature Portfolio wishes to improve the reproducibility of the work that we publish. This form provides structure for consistency and transparency in reporting. For further information on Nature Portfolio policies, see our Editorial Policies and the Editorial Policy Checklist.

Statistics

For all statistical analyses, confirm that the following items are present in the figure legend, table legend, main text, or Methods section.

n/a	Confirmed	
☐	☒	The exact sample size (n) for each experimental group/condition, given as a discrete number and unit of measurement
☐	☒	A statement on whether measurements were taken from distinct samples or whether the same sample was measured repeatedly
☐	☒	The statistical test(s) used AND whether they are one- or two-sided<br>*Only common tests should be described solely by name; describe more complex techniques in the Methods section.*
☒	☐	A description of all covariates tested
☐	☒	A description of any assumptions or corrections, such as tests of normality and adjustment for multiple comparisons
☐	☒	A full description of the statistical parameters including central tendency (e.g. means) or other basic estimates (e.g. regression coefficient) AND variation (e.g. standard deviation) or associated estimates of uncertainty (e.g. confidence intervals)
☐	☒	For null hypothesis testing, the test statistic (e.g. F, t, r) with confidence intervals, effect sizes, degrees of freedom and P value noted<br>*Give P values as exact values whenever suitable.*
☒	☐	For Bayesian analysis, information on the choice of priors and Markov chain Monte Carlo settings
☒	☐	For hierarchical and complex designs, identification of the appropriate level for tests and full reporting of outcomes
☒	☐	Estimates of effect sizes (e.g. Cohen's d, Pearson's r), indicating how they were calculated

Our web collection on statistics for biologists contains articles on many of the points above.

Software and code

Policy information about availability of computer code

Data collection

N/A

Data analysis

The below open source software were used for data analysis:
1) Structural Modelling:
1.1)Protein homology/analogy recognition server (Phyre2)
1.2)High Ambiguity Driven protein-protein DOCKing (HADDOCK)
1.3)CONTACT program within the CCP4 crystallographic software suite
1.4) PyMOL (Schrödinger)

2) Crystal structure refinement:
2.1) PHENIX
2.2) COOT

3) SEC-SAXS and batch-mode SAXS
3.1) ScÅtter software (v. IV.d, BIOISIS)
3.2) CHROMIXS
3.3) DENsity from Solution Scattering (DENSS)
3.4) PRIMUS
3.5) Fast SAXS Profile Computation algorithm (FoXS)
3.6) MULCh ModULes for the analysis of small-angle neutron Contrast variation data from bio-molecular assemblies
3.7) DAWN

3.8) Chimera (v. 1.19)

4) Protein quantification:
4.1) Image Lab v. 6.1, Bio-Rad
4.2) Image J software (v.1.54)

5) Statistical analysis
5.1) GraphPad Prism (v.10.5.0)

For manuscripts utilizing custom algorithms or software that are central to the research but not yet described in published literature, software must be made available to editors and reviewers. We strongly encourage code deposition in a community repository (e.g. GitHub). See the Nature Portfolio guidelines for submitting code & software for further information.

Data

Policy information about availability of data

All manuscripts must include a data availability statement. This statement should provide the following information, where applicable:

- Accession codes, unique identifiers, or web links for publicly available datasets
- A description of any restrictions on data availability
- For clinical datasets or third party data, please ensure that the statement adheres to our policy

The experimental data on which this manuscript is based is made freely available through the Open Science Framework (OSF) public data repository (with no access restrictions) with source data files including data underlying plots and uncropped SDS-PAGE gels under [DOI: 10.17605/OSF.IO/9TXMK] [48]. SAXS data are deposited in the Small Angle Scattering Biological Data Bank (SASBDB) under the following link [https://www.sasbdb.org/] and [reference IDs]: [SEC-SAXS Experiments: ACTN2-WT (SASDZJ4/K4), M92V (SASDZR5/Q5), R93Q (SASDZP5/N5), G111V (SASDZM5/L5), T247M (SASDZK5/J5), R327C (SASDZH5/G5), E448A (SASDZF5/D5), and R457C (SASDZE5/C5), with numbers corresponding to Peak 1/Peak 2]; [Batch-mode SAXS Experiments: ACTN2-WT (SASDZB5/A5/95), M92V (SASDZ85/75/65), R93Q (SASDZ55/45/35), G111V (SASDZ25/Z4/Y4), T247M (SASDZX4/W4/V4), R327C (SASDZU4/T4/S4), E448A (SASDZR4/Q4/P4), and R457C (SASDZN4/M4/L4), with numbers corresponding to dimer peak at 40/53/60°C]. The X-ray crystallography structures have been deposited in RCSB Protein Data Bank under accession codes: 9SIR [https://doi.org/10.2210/pdb9SIR/pdb] (Human Muscle Alpha-Actinin-2 Mutant R327C); 9SIS [https://doi.org/10.2210/pdb9SIR/pdb] (Human Muscle Alpha-Actinin-2 Mutant R457C). The previously published PDB files referred to in this study include: 4D1E [https://doi.org/10.2210/pdb4D1E/pdb], 6M5G [https://doi.org/10.2210/pdb6M5G/pdb], and 5A38 [https://doi.org/10.2210/pdb5A38/pdb].

Research involving human participants, their data, or biological material

Policy information about studies with human participants or human data. See also policy information about sex, gender (identity/presentation), and sexual orientation and race, ethnicity and racism.

Reporting on sex and gender	N/A
Reporting on race, ethnicity, or other socially relevant groupings	N/A
Population characteristics	N/A
Recruitment	N/A
Ethics oversight	N/A

Note that full information on the approval of the study protocol must also be provided in the manuscript.

Field-specific reporting

Please select the one below that is the best fit for your research. If you are not sure, read the appropriate sections before making your selection.

☒ Life sciences ☐ Behavioural & social sciences ☐ Ecological, evolutionary & environmental sciences

For a reference copy of the document with all sections, see nature.com/documents/nr-reporting-summary-flat.pdf

Life sciences study design

All studies must disclose on these points even when the disclosure is negative.

Sample size

Sample size was determined using three independent replicates for some experiments, while other experiment involved one independent replicate but with multiple experiments reporting same conclusion. No statistical methods were used to predetermine sample size.
Sample size for each experiment are reported in the corresponding figures and table legends. Replicates refer to measurements performed per individual sample or variant. Details for each experiment are as follows:
(1) Solubility assay; three independent replicates were performed, except for three variants (E448K, R759T, R796C) with two independent replicates (total n=18; WT and 17 variants).
(2) Differential Scanning Fluorimetry; two independent replicates were performed (total n=18; WT and 17 variants).
(3) Mass photometry (high salt incubation); one independent replicate was performed (total n=3; WT and 2 variants).

(4) X-ray crystallography; data collected from multiple crystals were analysed (total n=2; 2 variants).
(5) Actin-binding assay; multiple independent co-sedimentation runs were conducted at increasing concentrations of ACTN2 (total n=6; WT and 5 variants). Another series for WT, G111V, and T247M were also repeated with multiple independent co-sedemintation runs.
(6) Reciprocal actin-binding assay; three to four independent co-sedimentation runs were performed at increasing concentrations of actin (total n=3; WT and 2 variants).
(7) Thermolysin assay; one independent run is reported, with two additional runs performed for molar ratio optimization (total n=10; WT and 9 variants).
(8) SEC-MALS and SEC-SAXS; one independent experiment was performed for each technique. These complementary approaches yielded consistent results, supporting overall confidence in the findings (SEC-MALS: total n=10; WT and 9 variants); (SEC-SAXS: n=8; WT and 7 variants).
(9) Batch-mode SAXS; one independent measurement was performed at each of six temperatures (n=8; WT and 7 variants).
Given the large number of variants investigated, it is not feasible to perform multiple SAXS measurements for all samples. This limitation reflects constraints in beamline availability and the resource-intensive nature of these experiments.

Data exclusions

No data were excluded for any of experiments, with the exception apart of the actin-binding assays. In a revised set of experiments, higher concentrations of ACTN2 (up to 18 uM) were tested; however these measurements exhibited increased noise due to non-specific binding and absorption to plastic surfaces. Consequently, 12 uM was selected as the maximum concentration for all variants investigated. This concentration is consistent with the highest concentration used for ACTN2-WT in the initial submission.

Replication

All attempts for replication were successful. Replication strategies for each experiment are outlined below:
-We have performed three independent replicates for Solubility assay (except for E448K, R759T, R796C with two independent replicates) and two replicates for Differential Scanning Fluorimetry.
-We have performed one independent replicate for mass photometry using high salt incubation, and collected data from multiple crystals for X-ray crystallography analysis.
-We have employed two complementary techniques for actin-binding assay.
(1) Actin-binding assay with increasing ACTN2 concentration: we have performed multiple independent co-sedimentation runs at increasing concentrations of ACTN2. Another series for WT, G111V, and T247M were also repeated with multiple independent co-sedimentation runs.
(2) Reciprocal actin-binding assay with increasing actin concentration, we have performed three to four independent co-sedimentation runs at increasing concentrations of actin.
-We have reported one independent run for the thermolysin assay; two additional runs were conducted for optimization trials prior to data collection.
- We have used complementary techniques (SEC-MALS and SEC-SAXS) to show congruent results. One independent run was performed for each approach as they produced consistent results that strengthened confidence in the aggregation findings. Thus, replication was achieved through the use of independent methods confirming the same findings.
- We collected and analysed size data points for the batch-mode SAXS with one independent run at each of the six measured temperatures (40, 47, 53, 55, 57, and at 60 ° C).

Randomization

Randomisation of samples was not possible. All experiments included the control sample (ACTN2-WT) which was compared with the ACTN2 variants. The initial experiments included all 17 ACTN2 variants in relevant to WT. Selection of variants for further experiments was described in the manuscript's results section. First selection process involved the use of variants which showed slight, medium or extreme changes in melting temperatures, and the selected variants are nine. Additional selection was done after assessing aggregation with two variants not showing aggregation, thus the remaining experiments were focused on seven variants.

Blinding

Blinding was not feasible and is of limited relevance for these types of biochemical experiments for several reasons. The first author (M. Noureddine, MN) was solely responsible for data generation and analysis, with most experiments conducted at a different institution from the host laboratory, where no additional colleagues were available to enable blinded procedures. Furthermore, because MN carried out all experimental steps, from protein expression (or sample generation) through data collection and analysis, it was necessary to retain knowledge of sample identity for technical and logistical reasons. Nevertheless, given the large number of samples included in many experiments, individual variants were not readily distinguishable during the analysis stage, thereby reducing the potential for bias.

Reporting for specific materials, systems and methods

We require information from authors about some types of materials, experimental systems and methods used in many studies. Here, indicate whether each material, system or method listed is relevant to your study. If you are not sure if a list item applies to your research, read the appropriate section before selecting a response.

Materials & experimental systems

n/a	Involved in the study
☒	☐ Antibodies
☒	☐ Eukaryotic cell lines
☒	☐ Palaeontology and archaeology
☐	☒ Animals and other organisms
☒	☐ Clinical data
☒	☐ Dual use research of concern
☒	☐ Plants

Methods

n/a	Involved in the study
☒	☐ ChIP-seq
☒	☐ Flow cytometry
☒	☐ MRI-based neuroimaging

Animals and other research organisms

Policy information about studies involving animals; ARRIVE guidelines recommended for reporting animal research, and Sex and Gender in Research

Laboratory animals	Research organisms [DH5α and BL21-CodonPlus(DE3)-RP Escherichia. coli (E.coli) Competent Cells]
Wild animals	N/A
Reporting on sex	N/A
Field-collected samples	Bacterial cultures were performed according to manufacturer's instructions
Ethics oversight	N/A

Note that full information on the approval of the study protocol must also be provided in the manuscript.

Plants

Seed stocks	N/A
Novel plant genotypes	N/A
Authentication	N/A
