# Supplementary material for: Comprehensive biophysical and structural profiling of alpha-actinin-2 variants reveals mechanistic diversity in hypertrophic cardiomyopathy
Source: Nat Commun. 2026 Jul 21;17:6127. doi: 10.1038/s41467-026-75392-z (PMC13388997; doi:10.1038/s41467-026-75392-z)
Supplement: Supplementary file 3 — Transparent Peer Review file [file 41467_2026_75392_MOESM3_ESM.pdf]

nature portfolio

Peer Review File

Comprehensive Biophysical and Structural Profiling of Alpha-actinin-2 Variants Reveals Mechanistic Diversity in Hypertrophic Cardiomyopathy

Corresponding Author: Professor Katja Gehmlich

This file contains all reviewer reports in order by version, followed by all author rebuttals in order by version.

Version 0:

Reviewer comments:

Reviewer #1

(Remarks to the Author)
General Comment
The manuscript would benefit from more cautious interpretation, particularly with respect to disease relevance, to avoid overextending conclusions beyond what is directly supported by the structural data. Otherwise, the mechanistic insight into the behavior of ACTN2 is well done and combined with the other techniques is the start of a good pipeline. Some clarifications/revisions are needed:

Summary
1. SEC-SAXS frame selection and buffer subtraction
For the SEC-SAXS experiments (Figure 4), it is unclear which frames were used for buffer subtraction and which frames were averaged under the elution peaks. This information is essential for evaluating the data quality. In addition, do the Rg values vary across the elution peak, or are they constant? Clarification is needed.
2. Missing 1D SEC-SAXS scattering profiles
The manuscript does not include 1D SAXS profiles derived from the SEC-SAXS experiments. These profiles are shown for the batch SAXS data (Figure 5), but equivalent SEC-SAXS profiles are missing and should be provided.
3. Missing Kratky Analysis
A large fraction of the figures is devoted to Guinier fitting. This level of emphasis may not be necessary and could be reduced, e.g. including the Guinier fits as an inset. Doing so would free space for additional analyses, such as Kratky plots, which would be valuable for assessing the foldedness and flexibility of each construct. Although that analysis will reveal rod-like behavior, it would be interesting to observe changes across the variants.
4. DENSS models of aggregate peaks
Including DENSS reconstructions for the aggregate peaks in the main text does not appear necessary. In particular, overlays between aggregate models and the dimeric protein could be misleading and may be better suited for the Supplementary Information, if included at all.
5. Interpretation of the first elution peak (Line 321)
On Line 321, the authors state that the first elution peak corresponds to an aggregated species, yet a Guinier region is reportedly used to calculate an Rg. This would be inconsistent, as aggregated species do not exhibit a clean Guinier regime. Moreover, Figure 4C does not show a Guinier fit for this peak, making it difficult to assess the validity of this claim. If a linear Guinier region exists, the species would more likely be monodisperse and well-defined rather than aggregated.
6. Overinterpretation of clinical relevance
The manuscript places strong emphasis on clinical relevance, which seems overstated given the data presented. This work is primarily a structural and mechanistic study, and the connection to clinical implications does not need to be emphasized to the same extent—particularly since the clinical significance of some variants listed in the table is not yet well established.
7. Missing citation
○ BioXTAS RAW citation is missing.
8. FOXS fits
Could the authors please explain why they think the fits of PDB structure to SAXS profile in figure S12 is good? There seems to be a large mismatch at low-mid q in the fit.

Reviewer #2

(Remarks to the Author)
The authors investigate the structural and functional consequences of 17 HCM-linked missense variants in the ACTN2 gene encoding alpha-actinin-2, using a series of in vitro approaches with the full-length alpha-actinin-2 proteins. Five of the variants localize to the ABD, 10 to the rod domain composed of spectrin repeats (SR) domains, and 2 to the C-terminal CaM domain. All the variants showed modest to strong reduced solubility when expressed in bacteria, suggestive of possible protein destabilization, misfolding or aggregation. A predicted model of the alpha-actinin-2 ABD bound to actin was generated using HADDOCK software. From this model, most ABD variants were predicted to disrupt actin binding. Further, using a previously generated crystal structure of the alpha-actinin-2 dimer, SR domain located variants were predicted to disrupt salt bridges between monomers or to destabilize SR structure. Differential scanning fluorimetry denaturation studies showed that all ABD variants, except S147L, caused a moderate to extreme decrease in full-length protein stability. Similarly, 4 of 10 SR variants caused slight to moderate decreases in thermal stability. The authors performed additional studies on the variants, primarily those that decreased stability. Actin binding assays on the ABD variants suggest small changes in Kd and/or Bmax for all variants except one (M92V). Crystal structures were generated for R327C and R457C SR variants, predicted to disrupt dimer formation. The resulting structure did not show a defect in dimer formation and instead overlapped well with the wild-type structure. Thus, the structures seem to rule out extreme disruption to dimerization. Thermolysis proteolytic digests showed that most of the ABD and SR variants are more susceptible to digestion, or show different degradation patterns. This supports that many of the variants alter protein conformation. Further SEC coupled to MALS and SAXS support that many of the variants for aggregates or high mass oligomers, relative to wild-type.

Overall, this is an extensive in vitro characterization of a large number of HCM linked variants. Many of the variants cause thermal destabilization that appears linked to altered actin binding (for ABD variants) and altered protein conformation, makes variants prone to aggregation/high mass oligo formation. The work supports altered actin binding and aggregation as pathogenic molecular mechanisms.

There are some concerns with some data and there are ways that paper could be strengthened, as listed below.

Structural modeling
A structural model of the alpha-actinin-2 ABD bound to actin was generated using HADDOCK, with restraints based on actin binding residues that authors identified based a low resolution (16 angstrom) Galkin, 2010 cryo-EM structure of alpha-actinin-3 ABD bound to actin. The model also incorporated actin binding residue information from NMR data showing two different synthetic peptides from the related dyrstrophin ABD1 bind actin. It is unclear why more recent, much higher resolution cryo-EM structures of closely related tandem CH ABD proteins (filamin A, utrophin, beta-spectrin) bound to actin were not incorporated into their modeling or structural analyses.
How well does the authors' model resemble the experimentally determine filamin A, utrophin or beta-spectrin-actin stuctures? To validate their model, the authors should provide some information on how their predicted model compares to experimentally determined tandem CH ABD-actin structures. All of these tandem CH ABDs likely bind actin by a similar mechanism. How well do the models align? Are the surfaces on actin and the ABD involved in binding similar? Are the amino acids in alpha-actinin-2 that are impacted by cardiomyopathy missense variants conserved in the related tandem CH ABDs, and do these residues have similar ABD or actin contacts?

Are any of the seventeen variants conserved in the related proteins, and are any of these known to be mutation sites? The authors' models does not explain how M92V impact actin binding. If M92 is conserved in filamin A, utrophin or beta-spectrin, then the position of the homologous M92 residues in the related structures might better explain the impact of M92V on alpha-actinin-2 actin binding. Incorporating structural information from the related ABD-actin cryo-EM structures may broaden the impact of the article.

Actin binding assays
The binding data has significant scatter that might reflect the elevated background and nonuniform background observed in many of the raw co-sedimentation gels. The reported Kd and Bmax values should include a measure of error (standard deviation or confidence interval associated with the fit). The authors should state how many times the co-sedimentation assays were performed, and if the fitted Kd and Bmax values represent average values from multiple curve fits from replicate binding assays, or a single curve fit to binding data combined from multiple experiments. Without a measure of error/confidence, strong conclusions cannot be made about the Kd and Bmax values of WT vs variants.

In particular, for T247M, the authors conclude that the variant causes no change in Kd, but an increase in Bmax. However, the T247M binding data (compared to other variants) lacks higher alpha-actinin-2 concentration data, and the binding curve does not approach a plateau. Thus, the T247M data is insufficient to accurately fit Bmax. To conclude higher Bmax, the authors should extend the binding curve with additional data points using higher amounts of alpha-actinin.

As the authors reference, T247M and G111V ABD variants have been described previously. However, the current study contradicts prior studies on the effect of the variants on actin binding. The prior equilibrium binding studies reported that T247M and G111V increase and decrease, respectively, actin binding affinity. In the current work, the authors conclude the variants have the opposite effects. These differences are difficult to reconcile, and the authors point to the use of full-length protein (this study) versus isolated ABD. This could be the explanation, but why the ABD vs full-length proteins would behave differently is not clear.

An additional concern with the co-sed data is that even in the absence of actin, some alpha-actinin protein accumulate in the pellet. This may reflect the propensity of variants to aggregate. The authors set up the actin binding assays by using a fixed amount of F-actin (3 uM) and varying amount of alpha-actinin (0.25-10 uM). Thus, I expect that increasing amount of alpha-actinin to be present in the pellet, with increasing alpha-actinin concentration, independent of actin. This would confound the actin binding assay interpretation. A better approach may be to set up the binding assays with a fixed amount of alpha-actinin (~1uM), and increasing amount of F-actin. In this way, the amount of non-specific alpha-actinin in the pellet would be constant across binding reactions. If the cosed data for WT, T247M and G111V look similar to the original actin binding data presented by the authors, then there would be no need to repeat the binding reactions for all variants.

Thermolysis experiments
For ABD variants, such as T247M, it would be interesting perform thermolysin experiments in presence and absence of F-actin. For example, destabilizing variants such as T247M may promote opening of CH1-CH2 interface, exposing hydrophobic surfaces that would otherwise be buried. These hydrophobic surfaces may be buried upon actin binding, thereby stabilizing the variant protein. Similarly, destabilizing SR variants may be stabilized – some SR variants in alpha-actinin related proteins, have been shown to increase actin binding. This thermolysin experiment performed with and without actin could be very informative given the high abundance of actin in cells. The destabilizing effect of the variants may be “neutralized” in the presence of actin.

Reviewer #3

(Remarks to the Author)
This is a relatively comprehensive investigation into the effects of disease-causing mutations in striated muscle alpha-actinin-2 (ACTN2), using analyses of purified protein. The majority of these mutations have been implicated in hypertrophic cardiac myopathy, where they are found as heterozygous mutations (autosomal dominant). The authors investigate selected mutations throughout the molecule; 5 in the actin binding (CH domains), 10 in the rod (responsible for anti-parallel dimer formation) and 2 in the C-terminal calmodulin binding region (CaM). They use a combination of assays, including actin binding, stability assays, SEC MALS, SEC-SAXS, SAXS and molecular modelling, although not all mutants are tested in all assays. They solve the crystal structure for two of the rod domain mutants, showing that they have little effect.
The findings will be of interest to the striated muscle field, and those interested in hypertrophic cardiomyopathy.
There is little experimental work on the characterisation of these mutants in the literature. Predominantly, the effects of mutations are predicted from the known structure of ACTN2. One studu investigated the effects of 2 mutations (G111V and A119T) using isolated CH domains from ACTN2 (Haywood et al, ref 10 in this report); and a second investigated A119T, M228T and T247M in isolated CH domains (Atang et al., 2023: ref 11 in this report). A third study investigated differentiated human iPSCs expressing an ACTN2 mutant (Prondzynski et al., 2019: ref 7 in this report). Ref 10 additionally investigated wild type and mutant versions of full length ACTN2 tagged with mEos in adult cardiomyocytes, showing that the mutants were prone to aggregate, and showed less dynamic behaviour than WT ACTN2 in live cells. Thus, the current work represents a comprehensive analysis of many mutatations, on the behaviour of the isolated protein, not reported before.

Overall, the work supports the conclusions and claims, with some exceptions.
Results:
The experiments to demonstrate the increased aggregation propensity (Table 2, 3) and decreased thermal stability (Table 1) and so on seem to be reasonable.
However, the statistics are not clear. For example, in Table 4, statistics are provided for solubility yield, but nothing else. This is mainly because it is not always clear how many times the experiments were repeated. The solubility experiments comprise at least 3 replicates (Figure 1) and thus have reasonable statistics.
The structural analysis (section 2.4) was somewhat hard to follow.
From the methods, it seems that HADDOCK was used to model the actin-ACTN2 interface (there is a low resolution CryoEM structure of the CH1-actin interface (ACTN3), from Ed Egelman’s group (Galkin et al., NSMB, 2010: 3LUE)). It is possibly worth mentioning how their HADDOCK model compares to this. Alphafold is quite good at modelling interfaces now – did they also try this?
In the methods section, it says PHYRE was used to assess mutations (this is a protein fold recognition server); PYMOL used to assess these, and CONTACT used to assess stabilising residues in the ACTN2 dimer. However, these different approaches were not spelt out in the results section, and it is not clear what approach was used when and which structures from the PDB (or models) were used. This needs to be spelt out.
It is a shame that there are no molecular dynamics simulations (e.g. using AMBER), which could reveal the underlying thermal instability, and give more insight into all of the mutations.
The actin binding experiments (Section 2.5) only seem to have been done once. There are no representative gels for supernatant and pellet, and the individual measurements shown on the graphs seem highly variable (e.g. see Fig. 3C). The data for T247M seems to end at about 3 micromolar free ACTN2 concentration. The description of the methods for this is very minimal. They do not describe how binding affinity and binding capacity was calculated (no equation, no reference).
Fig 3H shows no effect of high salt and shaking on the mass of the constructs (for 2 of the mutants) but the sizes of the peaks are reduced for the mutants – why is this?
The methods for mass photometry are really minimal (section 4.7), and include a description of the high salt/shaking (which is not mass photometry). Normally, the histograms for the data are plotted together with the fits. I’m also surprised that higher molecular weights equivalent to aggregates of different sizes were not seen (Fig. 3H). Why does the X-axis start at a negative mass value?

Section 2.6: "R327C and R457C variants reveal no impact on dimerization".
– I'm not sure that obtaining a crystal structure is the way most people would investigate dimerization! Perhaps this title needs rewording to be more accurate.
Discussion:
The overall central finding that the mutants are less thermally stable does agree with previous observations and seems reasonable. However, in the discussion, I'm not sure their data is secure enough to contrast their measurements of binding to actin (1 repeat) with those in reference 10 (5 repeats). It's not entirely clear why the results would be different for the actin binding domain alone, compared to full length protein – could they explain this?
The comment regarding the need for 'complementary approaches' (page 12, line 492) – what does this mean? It is a shame that they did not use any cell-based assays to analyse full length ACTN2 mutations in this work (as in ref 10). Is this what they mean?
The comment that "Methodologically, bacterial expression systems pose greater challenges for expressing full length large proteins, such as the giant cardiac protein titin or myosin heavy chain beta (β-MHC)" is unclear. (Page 12, line 494). It's not just the size that is important. The only expression system for full length myosin heavy chain is cultured skeletal muscle cells (extensive work from Don Winkelmann's group) – as these are the only cells that generate correctly folded protein.

Methods:
The methods section is very short, and it is often unclear how one would repeat the work.

Version 1:

Reviewer comments:

Reviewer #1

(Remarks to the Author)
Thank you to the authors for addressing all comments. The manuscript reads well now and data clearly supports the conclusions.

Reviewer #2

(Remarks to the Author)
The authors did an excellent job addressing all of the concerns I raised in the original review. The added actin binding assays now resolve discrepancies with early works. The thermolysin experiments in the presence of actin showing stabilization of T247M provide important mechanistic insight, supporting increased actin binding as a driver of pathogenesis. The alignments with ACTN2 related disease proteins broaden impact and make it easier the reader to these findings on ACTN2 to studies of the equivalent mutations in the related spectrin family proteins. I think this work is of strong significance to the field and is suitable for publication in Nature Communications.

Reviewer #3

(Remarks to the Author)
The authors have performed additional experiments and have clarified explanations. They have responded well to the comments I had on the original version, and I do not have any further comments to make.

Open Access This Peer Review File is licensed under a Creative Commons Attribution 4.0 International License, which permits use, sharing, adaptation, distribution and reproduction in any medium or format, as long as you give appropriate credit to the original author(s) and the source, provide a link to the Creative Commons license, and indicate if changes were made.
In cases where reviewers are anonymous, credit should be given to 'Anonymous Referee' and the source.
The images or other third party material in this Peer Review File are included in the article's Creative Commons license, unless indicated otherwise in a credit line to the material. If material is not included in the article's Creative Commons license and your intended use is not permitted by statutory regulation or exceeds the permitted use, you will need to obtain permission directly from the copyright holder.
To view a copy of this license, visit https://creativecommons.org/licenses/by/4.0/

Response to Reviewers

Please note, line numbers in the response to reviewers refer to “tracked changes” documents of main manuscript and supplementary material provided as pdf documents.

Reviewer 1

The manuscript would benefit from more cautious interpretation, particularly with respect to disease relevance, to avoid overextending conclusions beyond what is directly supported by the structural data. Otherwise, the mechanistic insight into the behavior of ACTN2 is well done and combined with the other techniques is the start of a good pipeline.

Some clarifications/revisions are needed:

1.1. SEC-SAXS frame selection and buffer subtraction: For the SEC-SAXS experiments (Figure 4), it is unclear which frames were used for buffer subtraction and which frames were averaged under the elution peaks. This information is essential for evaluating the data quality. In addition, do the Rg values vary across the elution peak, or are they constant? Clarification is needed.

Our response:
We thank the reviewer for this important point. We have added a new Table in the Supplementary Materials (Table S5) summarising the number of frames used for buffer subtraction, as well as those selected and averaged for each elution peak. The corresponding methodology section has been revised accordingly (lines 366-369 in Supplementary Materials). As for the Rg values, these were constant across the elution peaks. To clarify this, we have included an additional figure for WT and all analysed variants (Figure S10). Data were plotted using CHROMIXS, and the new figure shows the selected background frames, sample frames and the Rg values across each elution peak. This is further described in the Figure S10 legend (lines 532-535 in Supplementary Materials).

1.2. Missing 1D SEC-SAXS scattering profiles: The manuscript does not include 1D SAXS profiles derived from the SEC-SAXS experiments. These profiles are shown for the batch SAXS data (Figure 5), but equivalent SEC-SAXS profiles are missing and should be provided.

Our response:
The manuscript has been revised to include the 1D SAXS profiles derived from the SEC-SAXS experiments (new Figs. S11 and S12). Each plot presents an overlay of the WT and variant scattering profiles for the first (P1) and second (P2) elution peaks. T247M-P1 shows the most pronounced effects (lines 435, 436).

1.3. Missing Kratky Analysis: A large fraction of the figures is devoted to Guinier fitting. This level of emphasis may not be necessary and could be reduced, e.g. including the Guinier fits as an inset. Doing so would free space for additional analyses, such as Kratky plots, which would be valuable for assessing the foldedness and flexibility of each construct. Although that analysis will reveal rod-like behavior, it would be interesting to observe changes across the variants.

Our response:

We appreciate the reviewer’s suggestion. We have revised the figures by moving the Guinier fits at 60°C to the Supplementary Figures (Figs. S15 and S19), which now present the fits for

all three temperatures (at 40, 53, and 60°C). We used the space made available to include Kratky plots at 60°C, as suggested (Fig. 6 and Fig. S14). The Kratky plots at 40 and 53°C have also been generated and added to the Supplementary Materials (new Figs. S16 and S20). The Kratky analysis indicates that all ACTN2 samples retain the characteristic profile expected for ACTN2, consistent with the overall architecture inferred from the SAXS data. The Kratky profiles are highly similar across all variants compared to WT, indicating that the variants do not measurably affect the overall foldedness of the constructs except for G111V and T247M at 53 and 60°C (lines 475-477, Figs. 6, Figs. S14, S16, S20).

1.4. DENSS models of aggregate peaks: Including DENSS reconstructions for the aggregate peaks in the main text does not appear necessary. In particular, overlays between aggregate models and the dimeric protein could be misleading and may be better suited for the Supplementary Information, if included at all.

Our response:

As requested by the reviewer, the DENSS models corresponding to the aggregated peaks have been removed from the SEC-SAXS profile figures. The DENSS models relating to the dimer peaks have been compiled into a separate figure and included in the Supplementary Materials (new Fig. S13). This change avoids potential misinterpretation arising from overlays between aggregate reconstructions and the dimeric protein while retaining the relevant structural information for reference.

1.5. Interpretation of the first elution peak (Line 321): On Line 321, the authors state that the first elution peak corresponds to an aggregated species, yet a Guinier region is reportedly used to calculate an Rg. This would be inconsistent, as aggregated species do not exhibit a clean Guinier regime. Moreover, Figure 4C does not show a Guinier fit for this peak, making it difficult to assess the validity of this claim. If a linear Guinier region exists, the species would more likely be monodisperse and well-defined rather than aggregated.

Our response:

Guinier fits for both the first (P1) and second (P2) elution peaks from the SEC-SAXS profiles have been included in the Supplementary Material (new Figs. S11 and S12). For the WT-P1 peak, the Guinier fit satisfies the Guinier approximation ($q.R_g$ <1.3), and an R_g*(Guinier)* value is therefore reported in Table 2. In contrast, the P1 peaks of the ACTN2 variants exhibit deviations from this fit (illustrated in Figs. S11, S12), and consequently no R_g*(Guinier)* values were reported, which is reflected by the blank entries in Table 2. The results section has been revised accordingly to clarify this point and avoid further ambiguity (lines 430, 431).

1.6. Overinterpretation of clinical relevance: The manuscript places strong emphasis on clinical relevance, which seems overstated given the data presented. This work is primarily a structural and mechanistic study, and the connection to clinical implications does not need to be emphasized to the same extent—particularly since the clinical significance of some variants listed in the table is not yet well established.

Our response:

Our conclusions and discussion points have been revised to avoid any overextension or overinterpretation of the data. Statements relating to clinical relevance have been moderated (lines 105, 107, 266). References to disease relevance have been adjusted to reflect the

current level of evidence and to acknowledge that the clinical significance of some variants remains to be fully established.

1.7. Missing citation: BioXTAS RAW citation is missing.

Our response:

BioXTAS RAW software was not used in any part of this study. Citations for the software packages used for data processing and analysis such as CHROMIXS and PRIMUS have now been included in the revised detailed methodology section (lines 369, 395 in Supplementary Materials).

1.8. FOXS fits: Could the authors please explain why they think the fits of PDB structure to SAXS profile in figure S12 is good? There seems to be a large mismatch at low-mid q in the fit.

Our response:

We have refined the wording of our analysis and omitted the term ‘good fit’ to avoid overstating the quality of model agreement. As noted by the reviewer, the FOXS fits show a lower agreement in the mid-to-high q range compared with the low-q range. We have therefore revised the text to indicate that, at 40°C, these experimental SAXS profile fits are broadly consistent with the overall size and shape of the ACTN2 crystal structure (lines 466-467). At 60°C, this fit becomes poorer at low-q range, particularly for the ABD variants (lines 483-484 and Figs. S18, S22).

General comment:

Please note that we have deposited SAXS data on the Small Angle Scattering Biological Data Bank (SASBDB) [pending approval] with draft IDs (7593-7608 for SEC-SAXS data, and 7609-7632 for SAXS batch-mode data), we will provide the access number(s) once the submission has been validated.

Please note, line numbers in the response to reviewers refer to "tracked changes" documents of main manuscript and supplementary material provided as pdf documents.

Reviewer 2:

The authors investigate the structural and functional consequences of 17 HCM-linked missense variants in the ACTN2 gene encoding alpha-actinin-2, using a series of in vitro approaches with the full-length alpha- actinin-2 proteins. Five of the variants localize to the ABD, 10 to the rod domain composed of spectrin repeats (SR) domains, and 2 to the C-terminal CaM domain. All the variants showed modest to strong reduced solubility when expressed in bacteria, suggestive of possible protein destabilization, misfolding or aggregation. A predicted model of the alpha-actinin-2 ABD bound to actin was generated using HADDOCK software. From this model, most ABD variants were predicted to disrupt actin binding. Further, using a previously generated crystal structure of the alpha-actinin-2 dimer, SR domain located variants were predicted to disrupt salt bridges between monomers or to destabilize SR structure. Differential scanning fluorimetry denaturation studies showed that all ABD variants, except S147L, caused a moderate to extreme decrease in full-length protein stability. Similarly, 4 of 10 SR variants caused slight to moderate decreases in thermal stability. The authors performed additional studies on the variants, primarily those that decreased stability. Actin binding assays on the ABD variants suggest small changes in Kd and/or Bmax for all variants except one (M92V). Crystal structures were generated for R327C and R457C SR variants, predicted to disrupt dimer formation. The resulting structure did not show a defect in dimer formation and instead overlapped well with the wild-type structure. Thus, the structures seem to rule out extreme disruption to dimerization. Thermolysis proteolytic digests showed that most of the ABD and SR variants are more susceptible to digestion, or show different degradation patterns. This supports that many of the variants alter protein conformation. Further SEC coupled to MALS and SAXS support that many of the variants for aggregates or high mass oligomers, relative to wild-type.

Overall, this is an extensive in vitro characterization of a large number of HCM linked variants. Many of the variants cause thermal destabilization that appears linked to altered actin binding (for ABD variants) and altered protein conformation, makes variants prone to aggregation/high mass oligo formation. The work supports altered actin binding and aggregation as pathogenic molecular mechanisms.

There are some concerns with some data and there are ways that paper could be strengthened, as listed below.

<u>Structural modeling</u>

2.1. A structural model of the alpha-actinin-2 ABD bound to actin was generated using HADDOCK, with restraints based on actin binding residues that authors identified based a low resolution (16 angstrom) Galkin, 2010 cryo-EM structure of alpha-actinin-3 ABD bound to actin. The model also incorporated actin binding residue information from NMR data showing two different synthetic peptides from the related dyrstrophin ABD1 bind actin. It is unclear why more recent, much higher resolution cryo-EM structures of closely related tandem CH ABD proteins (filamin A, utrophin, beta-spectrin) bound to actin were not incorporated into their modeling or structural analyses.

Our response:

The original HADDOCK-derived models of the ACTN2-ABD/actin complex were generated using restraints derived from the low resolution cryo-EM ACTN3-ABD/actin complex structure (15 Å), as ACTN3 represented the closest structural homologue to ACTN2 currently available in the PDB. Our modelling also incorporated actin-binding residue information derived from NMR studies showing that two synthetic peptides from dystrophin ABD1 interact with actin (PDB ID: 3LUE). In response to the reviewer's suggestion, we evaluated higher-resolution cryo-EM structures of related ABD-encompassing proteins bound to actin. Sequence comparison showed substantial similarity (~60%) between Utrophin-ABD and ACTN2-ABD, allowing us to incorporate restraints from the higher-resolution (3.6 Å) Utrophin/actin complex (PDB ID: 6M5G) into our modelling (new Fig. S3). Importantly, the updated HADDOCK-derived ACTN2/actin complex model, including those containing ACTN2 variants, closely resemble the experimentally determined Utrophin/actin complex, as well as the Filamin A/actin and β-III spectrin/actin complexes. These updated models have now replaced the original models in the revised manuscript. Consequently, the molecular rationale for several variants have been revised (lines 165-238, Fig. 2, Fig. S4), and the incorporated comparative structural analysis strengthens both the mechanistic interpretation and the impact of the study.

2.2. How well does the authors' model resemble the experimentally determine filamin A, utrophin or beta- spectrin-actin stuctures? To validate their model, the authors should provide some information on how their predicted model compares to experimentally determined tandem CH ABD-actin structures. All of these tandem CH ABDs likely bind actin by a similar mechanism. How well do the models align? Are the surfaces on actin and the ABD involved in binding similar? Are the amino acids in alpha-actinin-2 that are impacted by cardiomyopathy missense variants conserved in the related tandem CH ABDs, and do these residues have similar ABD or actin contacts?

Our response:

As noted in our response to point 2.1, our original docking profiles differed from those observed in experimentally determined Utrophin/actin, Filamin A/actin and β-III spectrin/actin complexes. However, our updated models, produce docking poses that more closely resemble the experimentally determined ABD-containing protein/actin structures. These revised models have been used in the subsequent analyses assessing the impact of HCM-associated variants on ACTN2-ABD actin binding. Importantly, residues contributing to the three principal actin-binding sites identified in Utrophin-CH1 domain are conserved in ACTN2-CH1 (new Figs. S2, S3), suggesting that these proteins are likely to adopt similar actin-binding interfaces and supports the structural and functional relevance of the revised ACTN2/actin model.

The reviewer also raises an important point regarding the evolutionary conservation of amino acids in ACTN2-ABD that are affected by cardiomyopathy-associated missense variants across related Utrophin, Filamin A and β-III spectrin ABDs. To address this, we performed sequence alignments comparing the ABDs of these proteins with the ACTN2-ABD. These analyses indicate that R93Q variant is conserved across all three homologous proteins. In contrast, T247 is conserved in Filamin A and β-III spectrin, M92 and S147 are conserved only in β-III-spectrin, and G111 is not conserved in any of the related proteins (new Fig. S2). These findings suggest that although the overall actin-binding mechanism is likely to be conserved across tandem CH domain-encompassing proteins, certain residues affected by cardiomyopathy-associated variants may contribute to structural stability, regulatory interactions, or functional properties that are specific to ACTN2. We have included a few sentences in the results (lines 166-173) and discussion (lines 599-602) to reflect these points.

2.3. Are any of the seventeen variants conserved in the related proteins, and are any of these known to be mutation sites? The authors' models does not explain how M92V impact actin binding. If M92 is conserved in filamin A, utrophin or beta-spectrin, then the position of the homologous M92 residues in the related structures might better explain the impact of M92V on alpha-actinin-2 actin binding. Incorporating structural information from the related ABD-actin cryo-EM structures may broaden the impact of the article.

Our response:

This is an interesting point raised by the reviewer. Our response to point 2.2 addresses the levels of conservation for the ABD variants between ACTN2 and related Utrophin, Filamin A and β-III spectrin proteins. With respect to rod domain variants, Filamin A lacks a comparable rod domain, hence our conservation analysis primarily focused on Utrophin and β-III spectrin. Of the ACTN2 rod domain variants, two (E448A/K) are conserved in both proteins, three (R327C, R353W, R457C) are conserved in β-III spectrin, one (I653T) is conserved in Utrophin, while the remaining four are not conserved in either protein (new Fig. S2). These findings indicate that although the overall role of the ACTN2 rod domain, particularly in maintaining structural stability and mediating dimerization, is likely to be conserved across Utrophin and β-III spectrin proteins, several residues affected by cardiomyopathy-associated variants appear to be less conserved. This suggests that these residues may contribute to structural stability, regulatory interactions, or functional properties that are specific to ACTN2.

In addition, we have assessed whether any of the ACTN2 seventeen variants correspond to known mutation sites in the related proteins. Using the human gene mutation database (HGMD), three β-III spectrin variants (R351P, R480W, R633Q), linked to the inherited neurodegenerative disease, spinocerebellar ataxia, were found to correspond to the ACTN2 variants (R327C, R457C, E628G). This has also been added as a discussion point (lines 602-605). No other ACTN2 variants were found in Utrophin or Filamin A.

In addition, as noted in our response to point 2.2, the M92V variant is conserved in Filamin A, supporting its potential functional importance. Following the reviewer's constructive suggestion, we repeated the structural modelling using restraints derived from the higher-resolution cryo-EM Utrophin/actin complex structure. Importantly, our revised models provide additional mechanistic insight into the pathogenicity of the M92V variant that could not be adequately explained by our previous model. Specifically, the updated model positions M92 near the actin-binding interface, supporting a direct structural basis for how the M92V substitution may perturb actin interaction (Fig. S3B and S4A). This comparative and structural analysis has now been incorporated into the revised manuscript, which we believe strengthens the mechanistic interpretation and broadens the impact of the study (lines 198-202: and Fig. S4A).

Actin binding assays

2.4. The binding data has significant scatter that might reflect the elevated background and nonuniform background observed in many of the raw co-sedimentation gels. The reported Kd and Bmax values should include a measure of error (standard deviation or confidence interval associated with the fit). The authors should state how many times the co-sedimentation assays were performed, and if the fitted Kd and Bmax values represent average values from multiple curve fits from replicate binding assays, or a single curve fit to binding data combined from multiple experiments. Without a measure of error/confidence, strong conclusions cannot be made about the Kd and Bmax values of WT vs variants.

Our response:

Based on the reviewer's excellent suggestions we have now performed more actin binding assays (see below) and re-analysed the existing datasets.

1) Number of repeats:

While experiments were performed in one session for five variants, each datapoint is an independent co-sedimentation run. To clarify, a single curve fit was performed for variants M92V, R93Q, and S147L variants.

For WT, and the key variants (G111V and T247M) we have performed further actin binding experiments:

a) We have repeated the original actin binding experiments with a wider range of concentrations (new Fig. 4A, B), and a single curve fit was used.

b) We have performed the reciprocal binding experiments (new Fig. 4C, D). This valuable suggestion from the reviewer allowed us to unmask altered binding behaviour of G111V in this assay.

2) Data variability:

We appreciate the reviewer's comment regarding the variability observed in the co-sedimentation data. The background signal observed in the raw co-sedimentation gels was corrected using Image lab (Bio-Rad) by measuring the background intensity in each well and subtracting it from the corresponding protein signal. This correction process has been explicitly described (lines 306-307 in Supplementary Materials). An example of this background correction is also provided in Figure A1 in the Appendix of this response letter (page 16). The same subtraction method has also been applied consistently to the newly generated gels.

To address the reviewer's concern regarding uncertainty in the reported binding parameters (Kd and Bmax), the confidence intervals have now been included. An R^2 value for each fit has been added to the results table to indicate the quality of the curve fits for the ACTN2 variants (see Table S4).

Regarding the number of experiments performed, the co-sedimentation experiments were carried out using independent replicates across a range of ACTN2 concentrations. Additional replicates have now been included, particularly for WT, G111V and T247M. These revisions are reflected in the updated figure (new Figure. 4A, B, Table S4), where each point represents a single independent co-sedimentation, and this has been clarified in the corresponding figure legends (line 938). The sigmoidal data were fitted using the Hill equation, consistent with approaches previously cited (Merza et.al [1]). For the reciprocal binding experiments for G111V and T247M, three to four independent replicates were performed at each actin concentration, and these data are presented as fraction of ACTN2 bound versus actin concentration (lines 944- 945 and new Fig. 4C, D, Table S4). For transparency, we now also show representative gels with total, supernatant, and pellet fractions of these experiments (new Fig. S6).

Finally, our conclusions regarding the calculated K_d and B_{max} values have been moderated in the revised manuscript to avoid overinterpretation of the binding differences between WT and variant proteins (lines 547-558).

2.5. In particular, for T247M, the authors conclude that the variant causes no change in Kd, but an increase in Bmax. However, the T247M binding data (compared to other variants) lacks higher alpha-actinin-2 concentration data, and the binding curve does not approach a plateau. Thus, the T247M data is insufficient to accurately fit Bmax. To conclude higher Bmax, the authors should extend the binding curve with additional data points using higher amounts of alpha-actinin.

Our response:

As suggested by the reviewer, additional data points were collected using a higher concentration of ACTN2 (up to 12 μM for T247M, compared with 8μM used previously). Equivalent measurements at 12 μM were also performed for WT to allow an appropriate comparison. The extended binding curve for T247M now reaches plateau (new Fig. 4B), allowing for a more accurate determination of the binding parameters.

For further clarification, the reciprocal actin-binding experiment for T247M was performed using increasing concentrations of actin, and the analysis of the updated data confirms that the Kd and Bmax for T247M are increased relative to WT (new Fig. 4C, D). These revisions have been incorporated into the results section to clarify the interpretation (lines 350-351).

2.6. As the authors reference, T247M and G111V ABD variants have been described previously. However, the current study contradicts prior studies on the effect of the variants on actin binding. The prior equilibrium binding studies reported that T247M and G111V increase and decrease, respectively, actin binding affinity. In the current work, the authors conclude the variants have the opposite effects. These differences are difficult to reconcile, and the authors point to the use of full-length protein (this study) versus isolated ABD. This could be the explanation, but why the ABD vs full-length proteins would behave differently is not clear.

Our response:

Previous literature using the ABD only construct reported that T247M reaches binding saturation across the full range of actin concentrations tested (Atang et al [2]). In our assays using full-length protein, we observed an increase in both the K_d and B_{max} values for T247M. Notably, our reciprocal experiment further reveals that T247M increase actin binding affinity, which is consistent with the saturation behaviour reported previously. For the G111V variant, earlier work using the isolated ABD reported decreased actin affinity (Haywood et al [3]). In our reciprocal binding experiment using full-length protein, we see an observable decrease in K_d and a decrease in B_{max}, indicating reduced actin binding relative to WT. Taken together, these findings indicate that the effects of T247M and G111V on actin binding are consistent between isolated ABD and full-length ACTN2 constructs.

2.7. An additional concern with the co-sed data is that even in the absence of actin, some alpha-actinin protein accumulate in the pellet. This may reflect the propensity of variants to aggregate. The authors set up the actin binding assays by using a fixed amount of F-actin (3 uM) and varying amount of alpha-actinin (0.25- 10 uM). Thus, I expect that increasing amount of alpha-actinin to be present in the pellet, with increasing alpha-actinin concentration, independent of actin. This would confound the actin binding assay interpretation. A better approach may be to set up the binding assays with a fixed amount of alpha-actinin (~1uM), and increasing amount of F-actin. In this way, the amount of non-specific alpha-actinin in the pellet would be constant across binding reactions. If the cosed data for WT, T247M and G111V look similar to the original actin binding data presented by the authors, then there would be no need to repeat the binding reactions for all variants.

Our response:

In response to this comment, the protein was first spun down using 5 ml ultracentrifuge tubes before starting the assay, and the ACTN2-containing supernatant was then used for the binding experiments. This has now been clarified in the detailed methodology section (lines 278-282 in Supplementary Materials). To account for the remaining ACTN2 protein observed in the pellet, we performed parallel control experiments consisting of ACTN2 alone. The band

intensity from these controls was then subtracted from the pelleted ACTN2 measured in the presence of actin (see the detailed calculation description in the Appendix of this letter, pages 17-18). Additional details of the calculations have also been incorporated into the detailed methodology (lines 305-311 in Supplementary Materials).

We thank the reviewer for the excellent suggestion of a reciprocal binding experiment, which has now been performed for the WT, G111V, and T247M variants using a fixed ACTN2 concentration and increasing actin concentrations (0-30μM) (new Fig. 4C, D, Table S4). The results from these experiments recapitulate the trends observed in our original binding assays, supporting the conclusions drawn from the initial dataset. As the reciprocal experiments yielded consistent results for the tested variants, we believe additional repetition across all variants is not necessary.

<u>Thermolysis experiments</u>

2.8. For ABD variants, such as T247M, it would be interesting perform thermolysin experiments in presence and absence of F-actin. For example, destabilizing variants such as T247M may promote opening of CH1-CH2 interface, exposing hydrophobic surfaces that would otherwise be buried. These hydrophobic surfaces maybe buried upon actin binding, thereby stabilizing the variant protein. Similarly, destabilizing SR variants may be stabilized – some SR variants in alpha-actinin related proteins, have been shown to increase actin binding. This thermolysin experiment performed with and without actin could be very informative given the high abundance of actin in cells. The destabilizing effect of the variants may be “neutralized” in the presence of actin.

<u>Our response:</u>

We thank the reviewer for this insightful and well-reasoned suggestion. We have performed the proposed thermolysis digestion experiments in the presence and absence of actin, and these have been incorporated into the revised manuscript. Briefly, actin alone remained stable under the experimental conditions (new Fig. 4E). The digestion profiles of WT and G111V were comparable in the presence or absence of actin (new Fig. 4F, G). In contrast, the T247M exhibited a clear actin-dependent stabilisation. When incubated alone, T247M was largely digested within 5 minutes. However, in complex with actin, T247M remained stable for up to 30 mins of thermolysin treatment and increased stability even after two hours compared to T247M digested in the absence of actin (new Fig. 4F, G). These findings support the reviewer’s hypothesis that actin binding stabilises the T247M variant, likely through engagement of the CH1–CH2 interface and burial of exposed hydrophobic surfaces, thereby partially neutralizing its destabilising effect. We believe this experiment significantly strengthens the mechanistic interpretation of the T247M variant (lines 362-376 and Fig. 4E-G).

As for the second point raised by the reviewer, variants within spectrin repeat (SR) domains have indeed been shown in related cytoskeletal proteins, such as β-III spectrin and Utrophin, to influence actin binding through long-range structural effects. Although the SR variants studied here are located within the ACTN2 central rod domain and do not directly participate in the canonical actin-binding interface, it is possible that they could modulate actin binding indirectly through allosteric effects on protein stability, domain orientation, or dimer architecture. A detailed investigation of these potential long-range structural effects would require additional biochemical and structural analyses beyond the scope of the present study. We agree that this represents an interesting avenue for future work and have now addressed this possibility in the discussion (lines 644-655).

Please note, line numbers in the response to reviewers refer to "tracked changes" documents of main manuscript and supplementary material provided as pdf documents.

Reviewer 3:

This is a relatively comprehensive investigation into the effects of disease-causing mutations in striated muscle alpha-actinin-2 (ACTN2), using analyses of purified protein. The majority of these mutations have been implicated in hypertrophic cardiac myopathy, where they are found as heterozygous mutations (autosomal dominant). The authors investigate selected mutations throughout the molecule; 5 in the actin binding (CH domains), 10 in the rod (responsible for anti-parallel dimer formation) and 2 in the C-terminal calmodulin binding region (CaM). They use a combination of assays, including actin binding, stability assays, SEC MALS, SEC-SAXS, SAXS and molecular modelling, although not all mutants are tested in all assays. They solve the crystal structure for two of the rod domain mutants, showing that they have little effect.

The findings will be of interest to the striated muscle field, and those interested in hypertrophic cardiomyopathy.

There is little experimental work on the characterisation of these mutants in the literature. Predominantly, the effects of mutations are predicted from the known structure of ACTN2. One studu investigated the effects of 2 mutations (G111V and A119T) using isolated CH domains from ACTN2 (Haywood et al, ref 10 in this report); and a second investigated A119T, M228T and T247M in isolated CH domains (Atang et al., 2023: ref 11 in this report). A third study investigated differentiated human iPSCs expressing an ACTN2 mutant (Prondzynski et al., 2019: ref 7 in this report). Ref 10 additionally investigated wild type and mutant versions of full length ACTN2 tagged with mEos in adult cardiomyocytes, showing that the mutants were prone to aggregate, and showed less dynamic behaviour than WT ACTN2 in live cells. Thus, the current work represents a comprehensive analysis of many mutatations, on the behaviour of the isolated protein, not reported before.

Overall, the work supports the conclusions and claims, with some exceptions. Results:

3.1. The experiments to demonstrate the increased aggregation propensity (Table 2, 3) and decreased thermal stability (Table 1) and so on seem to be reasonable. However, the statistics are not clear. For example, in Table 4, statistics are provided for solubility yield, but nothing else. This is mainly because it is not always clear how many times the experiments were repeated. The solubility experiments comprise at least 3 replicates (Figure 1) and thus have reasonable statistics.

Our response:

We have now updated all figure legends to clearly indicate the number of independent replicates performed. Specifically, these now state that the solubility assay was performed with three independent replicates, thermal analysis with two independent replicates, the actin co-sedimentation assay with multiple co-sedimentation runs performed at different ACTN2 concentrations, the reciprocal actin-binding assay with three to four independent co-sedimentation runs at different actin concentrations. The reported thermolysin data represent one independent replicate; however, in total two digestion experiments per variant were performed using different ACTN2:thermolysin molar ratios during assay optimisation.

For the SEC-MALS and SEC-SAXS experiments demonstrating aggregation, one independent replicate was analysed. The agreement between complementary techniques strengthens confidence in the observed aggregation behaviour. Similarly, the batch-mode SAXS analysis was performed using one independent replicate, with six data points collected

and analysed at each temperature. The number of repeats for these experiments have been reflected in relevant figure legends.

3.2. The structural analysis (section 2.4) was somewhat hard to follow.

Our response:
We apologise for the lack of clarity in the original structural analysis and have reorganized Section 2.4 into clearly defined sub-sections with explanatory subheadings to improve accessibility, particularly for non-specialist readers.

3.3. From the methods, it seems that HADDOCK was used to model the actin-ACTN2 interface (there is a low resolution CryoEM structure of the CH1-actin interface (ACTN3), from Ed Egelman's group (Galkin et al., NSMB, 2010: 3LUE)). It is possibly worth mentioning how their HADDOCK model compares to this. Alphafold is quite good at modelling interfaces now – did they also try this?

Our response:
As noted in our earlier response to Reviewer 2 (Point 2.1), our original ACTN2/actin docking models were broadly consistent with the low-resolution (15 Å) cryo-EM structure of the ACTN3-actin complex (PDB ID: 3LUE). This similarity is expected, as spatial restraints derived from this structure were incorporated during docking. To improve model accuracy, we have now repeated the HADDOCK docking using restraints derived from the higher-resolution cryo-EM Utrophin/actin complex structure (PDB ID 6M5G) (new Fig. S3). The resulting docking solutions show strong agreement with higher-resolution structures of the CH1 domains of Filamin A, Utrophin, and β-III spectrin bound to actin. We therefore consider these updated models to provide a more reliable structural framework for interpreting the effects of ACTN2 variants on actin binding (Fig. S4). This interpretation is further supported by the conservation of key actin-contacting residues within the CH1 domains of Utrophin, Filamin A, and β-III spectrin, which are also conserved in ACTN2, suggesting a similar binding mode (new Figs. S2 and S3).

We also explored AlphaFold-based predictions of the ACTN2–actin interface. However, the resulting models showed substantial divergence from experimentally determined high-resolution ABD/actin cryo-EM structures. For this reason, we elected not to use AlphaFold-derived models in our structural interpretation of ACTN2 ABD variants and their effects on actin binding.

3.4. In the methods section, it says PHYRE was used to assess mutations (this is a protein fold recognition server); PYMOL used to assess these, and CONTACT used to assess stabilising residues in the ACTN2 dimer. However, these different approaches were not spelt out in the results section, and it is not clear what approach was used when and which structures from the PDB (or models) were used. This needs to be spelt out.

Our response:

We apologise for the insufficient detail in the original manuscript, which may have limited clarity, and have now substantially revised the Methods and Results sections to provide a clearer description of the modelling workflow and the specific purpose of each tool (lines 146-155, 189-194 in Supplementary Materials). Briefly, PHYRE2 was used to generate homology-based structural models of ACTN2 variants. The CONTACT tool was used to identify molecular interactions in the ACTN2 wild-type and variant structures. PyMOL was subsequently used for structural visualisation, mapping of variants, and qualitative assessment of residue interactions within the protein structure. Additionally, the relevant Protein Data Bank entries

used for HADDOCK modelling and analysis are now explicitly stated (lines 159-186 in Supplementary Materials). We hope these revisions improve transparency and reproducibility and address the reviewer's concerns.

3.5. It is a shame that there are no molecular dynamics simulations (e.g. using AMBER), which could reveal the underlying thermal instability, and give more insight into all of the mutations.

Our response:

We thank the reviewer for this helpful suggestion and agree that molecular dynamics (MD) simulations could provide additional insight into the dynamic behaviour and potential thermal stability effects of ACTN2 variants. While MD approaches, have the potential to provide valuable information on protein dynamics, meaningful interpretation would likely require extensive simulations and careful validation, particularly given the size and complexity of ACTN2/actin assemblies. We therefore feel that such analyses fall beyond the scope of the present study but represent a worthwhile direction for future investigation (lines 655-658).

3.6. The actin binding experiments (Section 2.5) only seem to have been done once. There are no representative gels for supernatant and pellet and the individual measurements shown on the graphs seem highly variable (e.g. see Fig. 3C). The data for T247M seems to end at about 3 micromolar free ACTN2 concentration. The description of the methods for this is very minimal. They do not describe how binding affinity and binding capacity was calculated (no equation, no reference).

Our response:

We thank the reviewer for these comments and have revised the manuscript accordingly. Additional replicates have now been performed for the actin-binding experiments for the G111V and T247M variants (see our response to Reviewer 2, point 2.7). The updated graphs now include multiple data points at different ACTN2 concentrations, with each point representing an independent replicate (new Fig. 4A, B). Following the suggestion of reviewer 2, we also conducted reciprocal actin-binding experiments for WT and G111V and T247M variants using fixed ACNT2 concentration and increasing actin concentrations (new Fig. 4C, D). Three to four independent replicates were performed at each actin concentration. The figure legends have been updated to clearly indicate the number of repeats performed (lines 938, 944-945).

Representative gels for WT, T247M and G111V showing total, supernatant and pellet fractions at the different ACTN2 concentrations are now included. Equivalent representative gels have also been incorporated for the reciprocal actin-binding experiments (Figs. 4 A, C and Fig. S6). In addition, the concentration range used for T247M has been increased to 12 μM (compared to 8 μM used previously) allowing a more complete characterisation of the binding curve.

Finally, the methods section has been expanded to incorporate a detailed description of how binding affinity and binding capacity were calculated, including the equations used and appropriate references (lines 305-311 in Supplementary Materials).

3.7. Fig 3H shows no effect of high salt and shaking on the mass of the constructs (for 2 of the mutants) but the sizes of the peaks are reduced for the mutants – why is this? The methods for mass photometry are really minimal (section 4.7), and include a description of the high salt/shaking (which is not mass photometry). Normally, the histograms for the data are plotted together with the fits. I'm also surprised that higher molecular weights equivalent to aggregates of different sizes were not seen (Fig. 3H). Why does the X-axis start at a negative mass value?

Our response:

The approximate molecular mass of the constructs remained unchanged with between the 0 and 48 hour measurements based on the mass photometry estimates. While a reduction in peak amplitude was observed for some variants, the measured molecular mass distribution remained similar. The reduced peak height likely reflects differences in particle counts between measurements, which can arise from factors such as minor surface adsorption to the glass coverslip or normal variability in particle landing events during mass photometry measurements. We apologise for the minimal detail provided for the mass photometry experiments and have accordingly revised the methods section to include the acquisition parameters as well as further information on the high-salt incubation conditions (lines 213-225 in Supplementary Materials).

The figures have also been updated to include both the histograms and the corresponding Gaussian fits for all samples at both time points. In addition, the x-axis has been adjusted to start at zero for clarity (new Fig. 3A).

Regarding the absence of larger aggregates, the samples were purified by size-exclusion chromatography prior to analysis, and only fractions corresponding to the second elution peak (ACTN2 dimer) were used for mass photometry experiments. This step enriched the dominant dimeric species and reduced the presence of high-order aggregates, and this clarification has now been added to detailed methodology (lines 213-214 in Supplementary Materials). A small number of high-mass events are still detectable as minor peaks in the distribution but are not emphasised in the figure due to their very low abundance.

3.8. Section 2.6: “R327C and R457C variants reveal no impact on dimerization”.– I’m not sure that obtaining a crystal structure is the way most people would investigate dimerization!

Our response:
We agree that crystallography alone is not typically used as the primary method to assess dimerisation. We have therefore reorganised this section to first present the mass photometry data, which directly assesses the oligomeric state of the constructs under the applied chemical and mechanical stress conditions. We attempted crystallisation of several ACTN2 rod domain constructs harbouring the selected variants. Of these, only two yielded diffraction-grade crystals, enabling structure determination of ACTN2-R327C and ACTN2-R457C. The resulting structures show that that canonical ACTN2 rod-domain dimer is preserved in these variants, suggesting that their pathogenic effects may arise from mechanisms other than disruption of dimerisation. We have revised our discussion section for further clarification (lines 537-543).

3.9. Discussion: The overall central finding that the mutants are less thermally stable does agree with previous observations and seems reasonable. However, in the discussion, I’m not sure their data is secure enough to contrast their measurements of binding to actin (1 repeat) with those in reference 10 (5 repeats). It’s not entirely clear why the results would be different for the actin binding domain alone, compared to full length protein – could they explain this?

Our response:

To strengthen our actin-binding data, we performed additional repeats for WT, and key variants (G111V and T247M). We also conducted reciprocal actin-binding assays with three to four independent replicates. These data demonstrate that the effects of T247M and G111V on actin binding are consistent between isolated ABD and full-length ACTN2 constructs.

3.10. The comment regarding the need for ‘complementary approaches’ (page 12, line 492) – what does this mean? It is a shame that they did not use any cell-based assays to analyse full length ACTN2 mutations in this work (as in ref 10). Is this what they mean?

Our response:
In the revised manuscript, we have expanded the discussion to elaborate on the complementary experimental strategies that could further develop this work. These include structural approaches such as cryo-EM analysis of the full-length ACTN2/actin complex and additional crystallographic studies of ABD variants (lines 651-655). We also agree that cell-based assays would provide important functional insights. While our present study focuses on the biophysical and structural characterization of ACTN2 variants, we now explicitly discuss the use of cellular systems (e.g. neonatal rat cardiomyocytes transfection assays) as an important future direction to assess the functional implications of these variants in a physiological context (lines 633-636). Moreover, given the large number of variants analysed in this study, our aim was to establish a structural and biophysical framework to identify variants with the greatest potential structural impact. Such prioritisation could guide future cell-based studies toward variants most likely to produce functional effects (for example, the ABD variants M92V, R93Q, G111V, and T247M).

3.11. The comment that “Methodologically, bacterial expression systems pose greater challenges for expressing full length large proteins, such as the giant cardiac protein titin or myosin heavy chain beta (β-MHC)” is unclear. (Page 12, line 494). It’s not just the size that is important. The only expression system for full length myosin heavy chain is cultured skeletal muscle cells (extensive work from Don Winkelmann’s group) – as these are the only cells that generate correctly folded protein.

Our response:
We thank the reviewer for this helpful clarification. We agree that the challenges associated with expressing large sarcomeric proteins such as β-MHC are not solely related to protein size but also to the requirement for appropriate post-translational modifications and chaperones to ensure correct folding, modification and assembly. We have therefore revised the discussion section to clarify this point and referenced work on β-MHC from Don Winkelmann’s group in relation to this (lines 627–628).

3.12. Methods: The methods section is very short, and it is often unclear how one would repeat the work.

Our response:

The methods section in the main text and the detailed methodology provided in the Supplementary Materials have been expanded to include additional information on experimental procedures. We believe these additions provide sufficient detail to allow reproducibility of the work (lines 740-759 in main text and lines 148-194, 213-225, 276-334 in Supplementary Materials).

APPENDIX

Step 1: Selection of background area for each well
(Band 1: background; Band 2: sample protein)

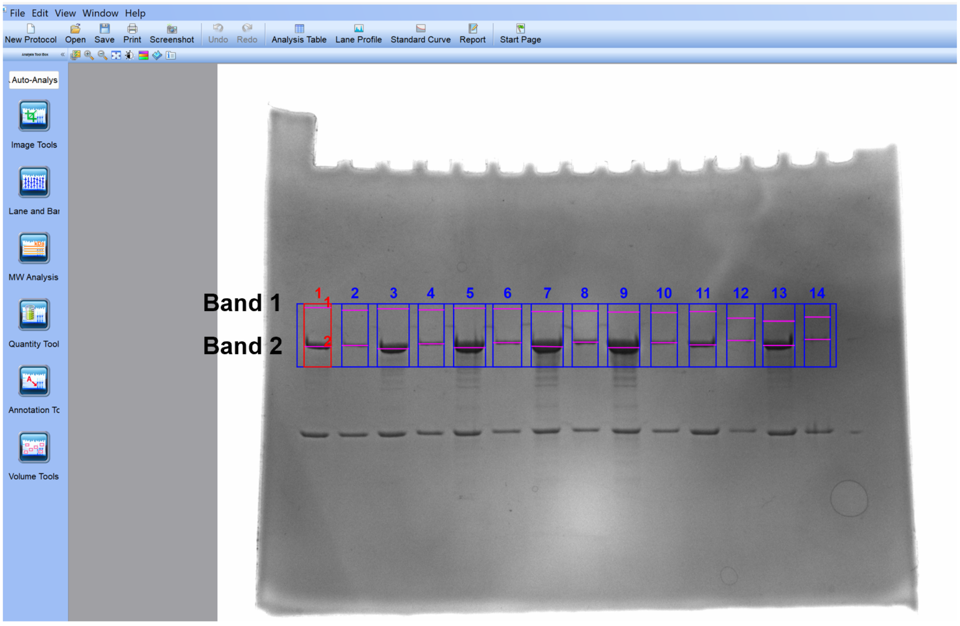

Step 2: Intensity detection for bands 1 and 2

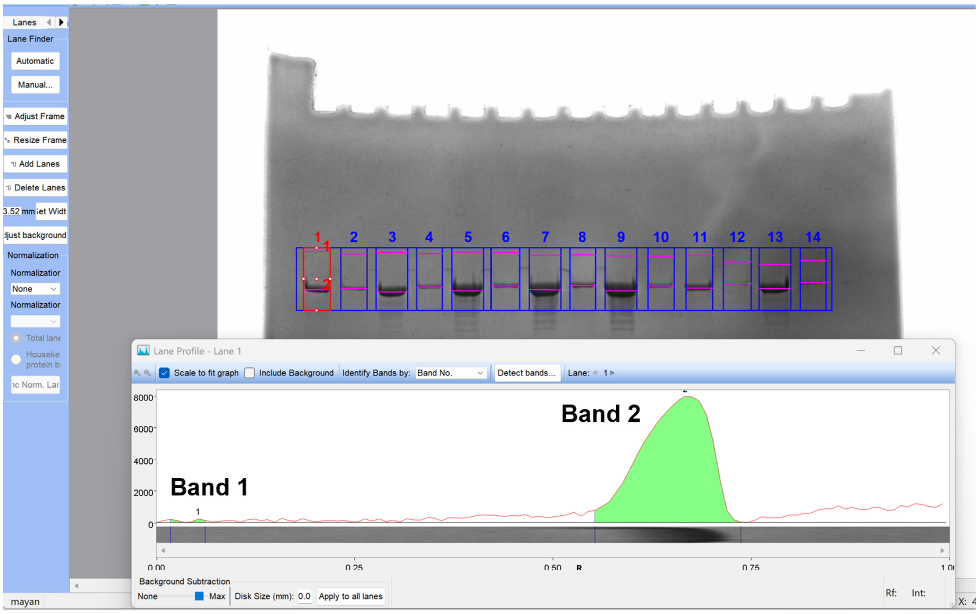

Step 3: Final sample band intensity = Band 2 intensity – Band 1 intensity (calculations are performed using excel)

Figure A1: Figure illustrating the background subtraction method used to account for elevated background, in which the band intensity of each sample is normalised to the background of its respective well to ensure accurate measurements.

Detailed Calculations for Actin-sedimentation Assays

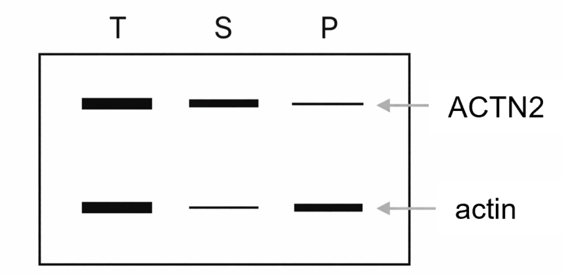

For the actin co-sedimentation assay calculations, it is assumed that "Total = Supernatant + Pellet"; therefore the Supernatant (or free protein) can be inferred by subtracting the pellet (bound protein) from the total. As a result, the supernatant fraction does not need to be analysed. To maintain consistency in SDS PAGE gel analysis, total and pellet fractions were run on the same gel wherever possible to minimize variability in Coomassie staining. Due to pipetting error, differential staining thresholds and background adjustment, the absolute raw densitometry values of the supernatant and pellet do not reliably add up to the total, and this discrepancy cannot be accurately accounted for when plotting bound versus free protein.

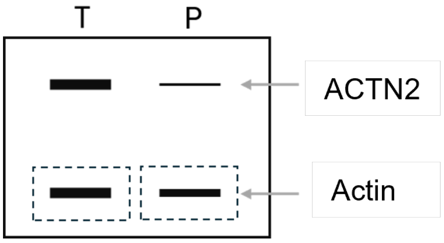

Densitometry is first performed on the actin band to calculate the relative concentration of actin recovered i.e. in this case 3 µM of actin was used and 2.5 µM was recovered in the pellet therefore ACTN2 densitometry was increased by 20% (or a factor of 1.2).

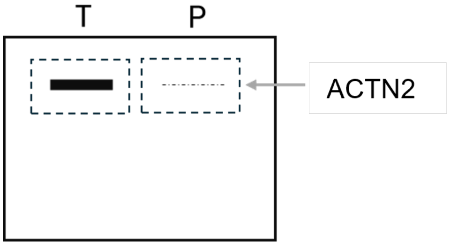

A separate actin free control was performed for each ACTN2 concentration and variant to assess the extent of actin independent sedimentation at each data point, this was factored into the final densitometry adjustments. For example, at 3 µM ACTN2, 0.15 µM was recovered in the pellet, and the corresponding densitometry from the actin sedimentation assay was therefore reduced by 5% (or a factor of -0.05).

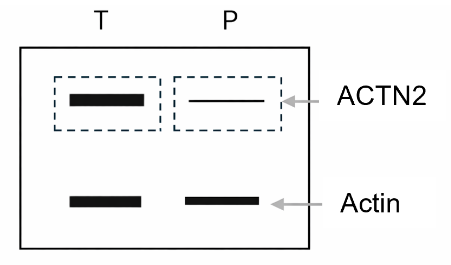

Finally, ACTN2 densitometry of ACTN2 values compared and adjusted by +20% and -5% to determine the bound concentration. For example, with a total ACTN2 concentration of 3 µM, and an initial recovery of 1 µM in the pellet, the adjusted bound concentration was 1.15 µM. Accordingly, the corresponding free (supernatant) concentration was calculated as 1.85 µM.

In principle, the same values could be derived by independently assessing the total and supernatant fractions to infer the pellet fraction. However, in practice, the adjusted supernatant and pellet values rarely add up to the known total due to cumulative errors arising from pipetting variability, background correction and other factors inherent to the analysis.

When plotting the bound versus free concentration, both axes represent variables, as previously reported by our group and others [1, 5]). Consequently, repeated measurements do not yield identical x-values with varying y-values, precluding the use of simple statistical tests such as T-test or ANOVA. Instead, data points corresponding to each fixed input concentration would cluster and vary along both axes, resulting in a single fitted curve with increased variability and, consequently, a lower R^2 value.

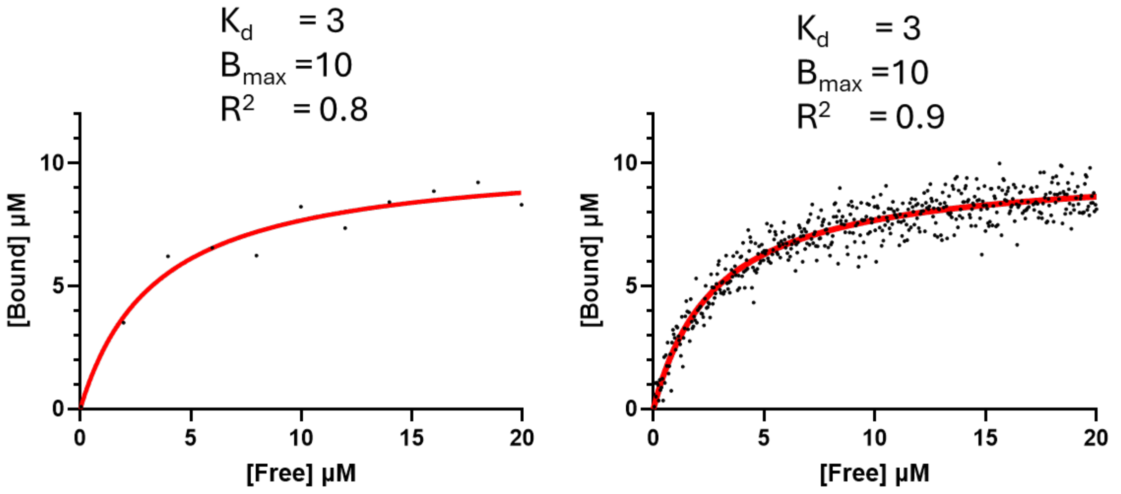

To reflect this and avoid overstating our conclusions, we have refrained from inferring statistically significant changes in K_d and/or B_{max} in the results and discussion sections. Additionally, we have included confidence intervals for all reported K_d and B_{max} values, as well as R^2 values for the corresponding line-fits.

References:

1. Mirza, M., et al., *The effect of mutations in alpha-tropomyosin (E40K and E54K) that cause familial dilated cardiomyopathy on the regulatory mechanism of cardiac muscle thin filaments*. J Biol Chem, 2007. **282**(18): p. 13487-97.
2. Atang, A.E., et al., *Cardiomyopathy-associated variants alter the structure and function of the α-actinin-2 actin-binding domain*. Biochem Biophys Res Commun, 2023. **670**: p. 12-18.
3. Haywood, N.J., et al., *Hypertrophic cardiomyopathy mutations in the calponin-homology domain of ACTN2 affect actin binding and cardiomyocyte Z-disc incorporation*. Biochem J, 2016. **473**(16): p. 2485-93.
4. Rybakova, I.N. and J.M. Ervasti, *Identification of Spectrin-like Repeats Required for High Affinity Utrophin-Actin Interaction**. Journal of Biological Chemistry, 2005. **280**(24): p. 23018-23023.
5. Heier, J.A., D.J. Dickinson, and A.V. Kwiatkowski, *Measuring Protein Binding to F-actin by Co-sedimentation*. J Vis Exp, 2017(123).
